# Integrating Clinical Priorities into the Technical Validation of Machine Learning Classifiers: A Generalizable Framework Demonstrated on Opioid Misuse Screening

**DOI:** 10.3390/jcm15187237

**Published:** 2026-09-17

**Authors:** Jacob Washton, Milan Toma

**Affiliations:** Laboratory of Algorithmic Medicine, Department of Osteopathic Manipulative Medicine, College of Osteopathic Medicine, New York Institute of Technology, Old Westbury, NY 11568, USA; jwashton@nyit.edu

**Keywords:** machine learning, clinical decision making, opioid misuse, screening, model validation, composite metrics, class imbalance

## Abstract

**Background/Objectives:** Traditional machine learning metrics often fail to capture the clinical consequences of classification errors, particularly in high-stakes screening applications. When applying task-specific AI tools to human lives, clinicians cannot rely on simple, headline metrics and marketing materials. Impressive accuracy figures can hide dangerous algorithmic shortcuts that collapse when encountering actual patients. This study addresses the gap between statistical performance and clinical utility by evaluating classifiers for prescription opioid misuse using a clinically oriented framework. **Methods:** Three ensemble models, namely, Cost-Sensitive Bagged Trees (CSBT-Untuned and CSBT-Tuned) and Random Undersampling Boosting (RUSBoost), were trained on National Survey on Drug Use and Health data and assessed using composite metrics integrating clinical priorities and asymmetric error costs. **Results:** The results demonstrate that while CSBT-Untuned achieved the highest raw accuracy of 86.7%, it missed 61.5% of positive cases. Conversely, the threshold-optimized CSBT-Tuned model achieved enhanced minority class detection and numerically higher Clinical Discriminative Performance Scores under sensitivity-priority scenarios, though its performance remained statistically comparable to RUSBoost given overlapping confidence intervals. Learning curve analysis confirmed stable convergence for the cost-sensitive bagging approach. **Conclusions:** Before accepting AI tools in patient care, physicians must demand an evaluation report similarly detailed to this manuscript, utilizing this framework as guidance for what an evidence report should demonstrate prior to clinical deployment.

## 1. Introduction

The clinical integration of artificial intelligence is accelerating, with diagnostic aids, predictive risk models, and decision support systems emerging across medical specialties at a pace that evaluation frameworks oriented toward technical validation have failed to match [1]. Central to this challenge is the recognition that evaluation methodology itself has become the critical bottleneck, i.e., the question is no longer whether machine learning models can achieve strong predictive performance, but whether the criteria used to assess that performance reflect the priorities of clinicians and the outcomes that matter to patients.

### 1.1. Background and Clinical Motivation

The evaluation of machine learning classifiers in medical contexts presents a fundamental challenge: traditional statistical performance metrics, while mathematically rigorous, often fail to capture the clinical consequences that ultimately determine a model’s value for patient care [1,2]. This limitation is evident across diverse prediction domains. In healthcare mortality prediction, studies employing deep learning architectures such as convolutional neural networks with gated recurrent units have achieved strong performance as measured by mean absolute error, mean squared error, and coefficient of determination, yet these conventional regression metrics provide no mechanism for encoding the asymmetric clinical consequences of prediction errors [3]. Similarly, classification studies in domains ranging from acoustic gender recognition [4] to behavioral survey analysis [5] have reported high raw accuracy and F1 scores without addressing whether such metrics adequately reflect the decision-relevant properties of the prediction task. The weighted linear combination follows from multi-attribute utility theory and aligns with clinical guidelines on healthcare composite performance assessment [6], which establishes that when component attributes are mutually preferentially independent, a decision maker’s preferences can be represented as an additive value function over the individual attributes. Importantly, this disconnect between predictive sophistication and evaluation adequacy is not unique to healthcare. Recent advances in ensemble learning and hybrid architectures have demonstrated the capacity of tree-based and deep learning models to achieve strong predictive performance across diverse high-stakes forecasting applications, from agricultural yield prediction using gradient boosting with meta-heuristic optimization [7] to climate variable forecasting using hybrid CNN-ResNet-LSTM architectures for renewable energy decision support [8] and emissions prediction using deep neural network and gradient boosting frameworks [9]. In each of these domains, as in healthcare, decision makers face asymmetric consequences: underestimating crop yield affects food security planning differently than overestimation, and underestimating emissions carries different policy implications than overestimation. Yet these methodological advances have largely proceeded independently of efforts to develop decision-analytic evaluation criteria appropriate for contexts where error costs are asymmetric and stakes are high. The result is a proliferation of sophisticated predictive models evaluated using metrics that, while statistically sound, fail to capture the priorities of decision makers. This pattern is especially consequential in medical applications, where the asymmetry between missed diagnoses and false alarms can translate directly into patient harm, making healthcare the focus of the present work.

The problem of predicting lifetime prescription opioid misuse using data from the National Survey on Drug Use and Health (NSDUH) is used to apply a clinically oriented evaluation protocol, designed to bridge this gap through composite metrics that combine multiple performance dimensions with adjustable weights reflecting clinical priorities. The problem of predicting lifetime prescription opioid misuse is a classification task characterized by substantial class imbalance and asymmetric error costs that render traditional accuracy metrics particularly misleading [10,11,12]. This accuracy paradox, wherein high overall accuracy obscures poor performance on the clinically critical minority class, is not unique to medical screening but is rarely addressed in machine learning evaluation practice. Studies achieving classification accuracies exceeding 99% on balanced datasets [4] demonstrate that raw accuracy can serve as a meaningful performance indicator when class distributions are symmetric and error costs are equivalent, but these conditions seldom hold in clinical contexts where the base rate of the target condition may be low and the consequences of missed cases may substantially exceed the consequences of false alarms. Beyond applying the foundational metrics, a detailed suite of methodological extensions addressing practical limitations inherent in any weighting-based evaluation approach was developed and demonstrated in this study. Building on algorithmic foundations shared with ensemble architectures deployed across non-medical domains [4,7,9], this study extends beyond classifier training to focus explicitly on evaluation methodology. Recognizing that a model’s clinical value depends on how statistical performance translates into patient outcomes under asymmetric cost structures, the present work formalizes a decision-analytic evaluation framework tailored to healthcare screening.

### 1.2. The Challenge of Opioid Misuse Prediction

Prescription opioid misuse represents a significant public health concern, with millions of individuals affected annually and substantial associated morbidity, mortality, and healthcare costs [13,14]. Early identification of individuals with opioid misuse history enables targeted intervention, risk-stratified prescribing practices, and appropriate allocation of treatment resources [15,16,17,18]. Machine learning classifiers trained on demographic and behavioral survey data offer potential for population-level screening, yet their evaluation requires careful consideration of the clinical context in which they would be deployed [11,19,20]. However, existing machine learning studies in this domain predominantly rely on conventional metrics evaluated in isolation without explicit integration of clinical priorities. Because context-agnostic indices summarize performance across arbitrary operating boundaries or conflate majority class true negatives with minority class case detection, they fail to provide a principled mechanism for trading off sensitivity against specificity according to clinical stakes. This gap motivates our decision-analytic framework.

The prediction of opioid misuse exemplifies the challenges that motivate clinically oriented evaluation. Class imbalance is pronounced: in the NSDUH sample examined here, only 11.7% of respondents report lifetime prescription opioid misuse, creating a majority-class-dominated accuracy landscape where a trivial classifier predicting all-negative would achieve nearly 90% accuracy while providing no clinical value. Error costs are asymmetric: missing an individual with opioid misuse history (false negative) may delay intervention during a critical window, while incorrectly flagging an individual without such history (false positive) imposes assessment burden but typically carries lower clinical consequence. These characteristics demand evaluation metrics sensitive to minority class detection and capable of incorporating differential error costs.

### 1.3. Study Objectives and Organization

The primary objective of this study is to evaluate machine learning classifiers for prescription opioid misuse prediction using a clinically oriented evaluation framework. This framework is distinct from conventional machine learning evaluation, which relies on context-agnostic metrics such as accuracy, F1 score, or area under the receiver operating characteristic curve that treat all classification errors as equivalent. It also differs from generic multi-criteria decision-making (MCDM) approaches such as the Analytic Hierarchy Process or TOPSIS, which, although capable of synthesizing multiple objectives, typically lack the domain-specific parameterization required for medical contexts and do not directly encode the asymmetric clinical consequences inherent in diagnostic decision making [21]. This approach moves beyond traditional statistical metrics by integrating medical priorities and asymmetric error costs into the technical validation process through explicitly parameterized weighting schemes that embed clinical judgment directly into the evaluation structure. We demonstrate this framework through four composite metrics and a suite of methodological extensions designed to address practical deployment challenges such as weight selection, uncertainty quantification, and decision-analytic integration.

Three classification approaches are compared: cost-sensitive bagged trees with default threshold (CSBT-Untuned), cost-sensitive bagged trees with optimized threshold (CSBT-Tuned), and Random Undersampling Boosting (RUSBoost). Their comparative evaluation illuminates how different clinical priorities yield different model recommendations, providing a template for practitioners to adapt to other medical classification problems.

The secondary objective is to demonstrate the practical application of the methodological extensions, providing a template for comprehensive clinically oriented evaluation that practitioners can adapt to other medical classification problems. The modular nature of the framework is emphasized: not every extension is required for every application, and practitioners should select those appropriate to the specific demands of each clinical context.

The framework presented here provides a principled approach to medical machine learning evaluation that respects both the rigor of statistical analysis and the complexity of clinical decision making. As physicians and end users of diagnostic tools, clinicians are directly responsible for decisions that alter patient lives and cannot outsource their clinical judgment to marketing materials. Would a practitioner trust an AI tool boasting 95% accuracy over one claiming 85%? In reality, impressive aggregate accuracy numbers frequently mask algorithmic shortcuts that collapse the moment the model encounters actual patients.

However, it must be recognized that machine learning models, much like large language models, are fundamentally statistical tools. They function effectively for individuals who fit the training statistics, and they cannot be expected to serve as confirmatory instruments because specific patients will always exist whose unique clinical presentations cannot be described by aggregate data. Using these tools is essentially identifying a specific condition in the patient at hand based on nationwide statistics, concluding that because most people in a similar situation had condition A, this patient has it too, even though their unique situation could be explained by other factors. The mathematics provides structure and precision; clinical expertise provides the necessary priorities, weights, and individual nuance; together, they enable evaluation that serves the ultimate purpose of medical artificial intelligence, i.e., the improvement of patient care.

## 2. Materials and Methods

This section details the empirical pipeline and decision-analytic evaluation protocol developed to assess machine learning classifiers under clinical priority constraints. The methodological workflow encompasses dataset construction and preprocessing, ensemble model training across three distinct classification pipelines, learning dynamics evaluation, and a multidimensional clinical validation framework incorporating cost matrices, expert swing-weight elicitation, net benefit analysis, and cost-effectiveness modeling.

### 2.1. Study Design and Data Source

The present study employed a supervised machine learning approach to predict lifetime prescription opioid misuse using data derived from the National Survey on Drug Use and Health (NSDUH) spanning the years 2015 through 2019. The outcome variable was defined as a binary indicator representing any history of prescription pain reliever misuse, constructed from the original survey variable pnrnmrec. Based on the NSDUH codebook, respondents with codes indicating misuse within the past 30 days (code 1), misuse more than 30 days ago but within the past 12 months (code 2), or misuse more than 12 months ago (code 3) were classified as positive cases, whereas respondents coded as never having used or misused pain relievers (code 91) were classified as negative cases. Observations with ambiguous or missing outcome values were excluded from analysis.

### 2.2. Data Preprocessing

All preprocessing operations were fitted exclusively on the training partition to prevent information leakage from validation and test sets; specifically, median imputation parameters and z-score scaling parameters ($\mu_{j},\sigma_{j}$) were calculated solely from training observations and subsequently applied to transform validation and test vectors without updating or re-fitting. Survey response codes commonly representing missing, refused, or unknown values (specifically codes 85, 94, 97, 98, and 99) were converted to missing indicators. Observations exceeding 50 percent missingness across predictor variables were removed from the training set. Variables potentially exhibiting target leakage, including alternative encodings of the outcome variable such as pnrnmyr, pnrnmevr, pnrnm30d, and pnrnm30fq, were identified and excluded prior to model development.

Numeric predictors underwent median imputation followed by z-score standardization, wherein each feature $x_{j}$ was transformed according to

(1) $$z_{j}=\frac{x_{j}-\mu_{j}}{\sigma_{j}}$$

where $\mu_{j}$ and $\sigma_{j}$ denote the mean and standard deviation of feature *j* computed from the training set. Categorical predictors were converted to dummy variables using one-hot encoding with the first category serving as the reference level. Features exhibiting zero variance in the training set were removed from all partitions. Any residual missing values following imputation were replaced with zeros. The complete preprocessing procedure, including the fitting and transformation functions, is formally specified in Algorithm A1.

### 2.3. Data Partitioning

**Figure 1:**
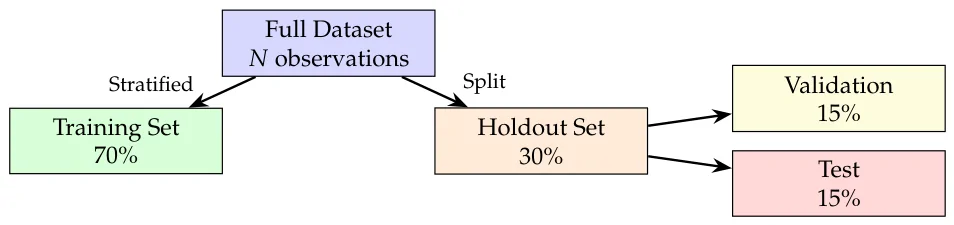
Stratified data partitioning scheme. The full dataset is initially divided into training (70%) and holdout (30%) subsets, with the holdout portion subsequently split equally into validation and test sets.

The preprocessed dataset was divided into three mutually exclusive subsets using stratified random sampling to preserve class proportions across partitions. The primary partition allocated 70 percent of observations to the training set, with the remaining 30 percent subsequently split equally between validation and test sets, yielding a final distribution of approximately 70 percent training, 15 percent validation, and 15 percent testing. The validation set served exclusively for hyperparameter assessment and threshold optimization, whereas the test set was reserved in strict holdout isolation for final unbiased performance evaluation, ensuring that test data played no role in feature transformation, hyperparameter selection, or decision boundary calibration. Figure 1 illustrates the data partitioning strategy employed across all three modeling pipelines.

### 2.4. Classification Models

**Figure 2:**
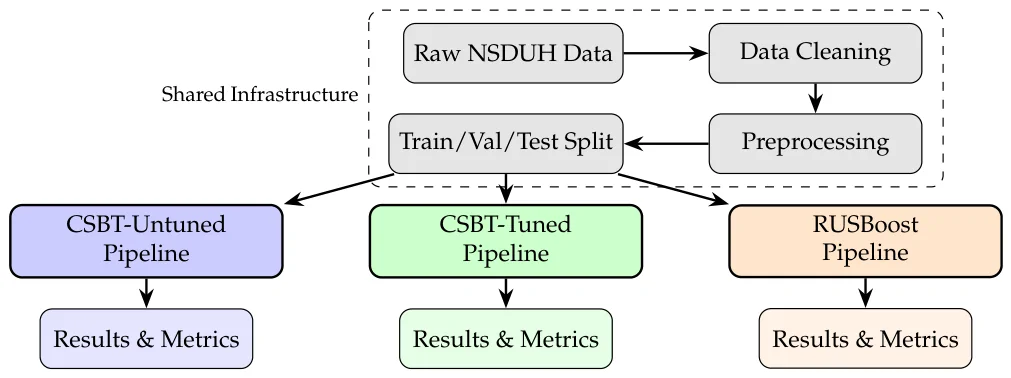
Relationship among the three classification pipelines. All models share identical data cleaning, preprocessing, and partitioning procedures, diverging only at the model training and evaluation stages.

Three ensemble classification models were developed and evaluated: Cost-Sensitive Bagged Trees without threshold tuning (CSBT-Untuned), Cost-Sensitive Bagged Trees with threshold tuning (CSBT-Tuned), and Random Under-Sampling Boosting (RUSBoost). All models utilized decision trees as base learners within their respective ensemble frameworks. The relationship among the three modeling pipelines is depicted in Figure 2, which illustrates the shared preprocessing infrastructure and divergent model-specific components.

#### 2.4.1. Cost-Sensitive Bagged Trees

**Figure 3:**
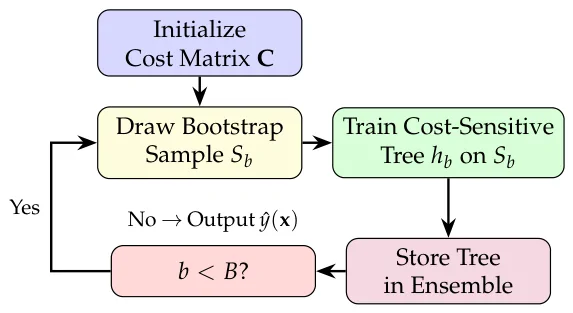
Internal iteration structure of the Cost-Sensitive Bagged Trees algorithm showing the bootstrap sampling and cost-sensitive tree training cycle repeated for *B* iterations.

The Cost-Sensitive Bagged Trees models employed bootstrap aggregation (bagging) combined with asymmetric misclassification costs to address class imbalance. Bagging constructs an ensemble of *B* decision trees, each trained on a bootstrap sample drawn with replacement from the training data. For a new observation x, the ensemble prediction is obtained by aggregating individual tree predictions. In the classification setting, each tree $h_{b}\left(x\right)$ casts a vote, and the final predicted class is determined by

(2) $$\hat{y}\left(x\right)=\underset{c\in \{0,1\}}{\arg\max}\sum_{b=1}^{B}I\left(h_{b}\left(x\right)=c\right)$$

where I(·) denotes the indicator function, and *B* represents the total number of trees in the ensemble. The internal iteration structure of the bagging procedure is depicted in Figure 3.

To account for the differential consequences of classification errors in the context of opioid misuse prediction, a cost matrix C was incorporated during training. The cost matrix penalizes false negatives (missed cases of misuse) more heavily than false positives, reflecting the clinical importance of identifying individuals at risk. The cost matrix was specified as

(3) $$C=\left(\begin{array}{cc} 0 & c_{FP}\\ c_{FN} & 0 \end{array}\right)$$

where $c_{FP}=1$ denotes the cost of a false positive, and $c_{FN}=3$ denotes the cost of a false negative. During tree construction, split criteria incorporate these costs to minimize expected misclassification cost rather than simple error rate.

The CSBT-Untuned model employed the default decision threshold of 0.5 for converting predicted probabilities to class labels. The CSBT-Tuned model extended this approach by optimizing the decision threshold exclusively on the validation set, guaranteeing that no information from the independent test set was utilized during calibration. For threshold tuning, predicted probabilities $\hat{p}\left(x\right)$ generated on validation feature vectors $X_{\mathrm{val}}$ were converted to class labels according to

(4) $$\hat{y}\left(x\right)=\left\{\begin{array}{cc} 1 & \mathrm{if}\;\hat{p}\left(x\right)\geq \tau \\ 0 & \mathrm{otherwise} \end{array}\right.$$

The threshold optimization employed a grid search over the interval [0.05,0.95] with increments of 0.01, evaluating each candidate threshold τ against the validation set, thereby completely decoupling decision boundary calibration from both initial tree ensemble training and final test set evaluation. The optimal threshold $\tau^{*}$ was selected as

(5) $$\tau^{*}=\underset{\tau \in \{0.05,0.06,\ldots ,0.95\}}{\arg\max}\;F_{1}\left(\tau \right)$$

where $F_{1}\left(\tau \right)$ denotes the F1 score achieved on the validation set at threshold τ; once determined, $\tau^{*}$ was locked and applied directly to the unseen holdout test set without further modification. This selection criterion balances precision and recall, providing a principled approach to threshold selection that accounts for class imbalance. The complete pseudocode for the CSBT-Untuned pipeline is provided in Algorithm A2, while the threshold-tuned variant is detailed in Algorithm A3.

#### 2.4.2. RUSBoost

**Figure 4:**
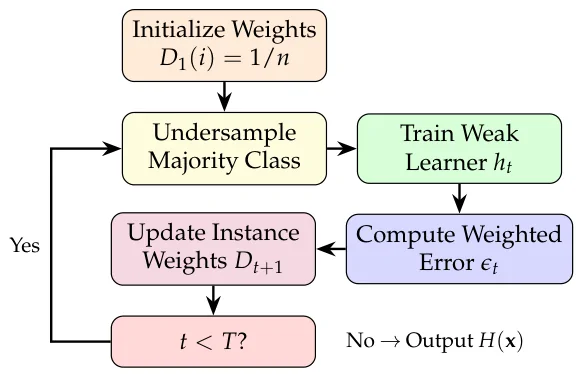
Internal iteration structure of the RUSBoost algorithm showing the undersampling and weight update cycle repeated for *T* iterations.

The RUSBoost algorithm combines random undersampling of the majority class with boosting to address class imbalance. At each boosting iteration *t*, the algorithm creates a balanced training subset by randomly undersampling the majority class to match the minority class size [22,23]. A weak learner (decision tree) is then trained on this balanced subset, and instance weights are updated based on classification performance. The internal iteration structure is depicted in Figure 4.

The boosting procedure maintains a weight distribution $D_{t}\left(i\right)$ over training instances. After training weak learner $h_{t}$, the weighted error is computed as

(6) $$\epsilon_{t}=\sum_{i:h_{t}\left(x_{i}\right)\neq y_{i}}D_{t}\left(i\right)$$

The learner weight $\alpha_{t}$ is then calculated based on the error rate, and instance weights are updated to emphasize misclassified observations in subsequent iterations. Specifically, the learner weight is computed as

(7) $$\alpha_{t}=\eta \cdot \frac{1}{2}\ln\left(\frac{1-\epsilon_{t}}{\epsilon_{t}}\right)$$

where η denotes the learning rate parameter that controls the contribution of each weak learner to the ensemble. The final ensemble prediction combines all weak learners according to

(8) $$H\left(x\right)=\mathrm{sign}\left(\sum_{t=1}^{T}\alpha_{t}h_{t}\left(x\right)\right)$$

where *T* denotes the total number of boosting iterations. The workflow for the RUSBoost pipeline is illustrated in Figure 4, with complete pseudocode provided in Algorithm A4.

#### 2.4.3. Unified Model Workflows

**Figure 5:**
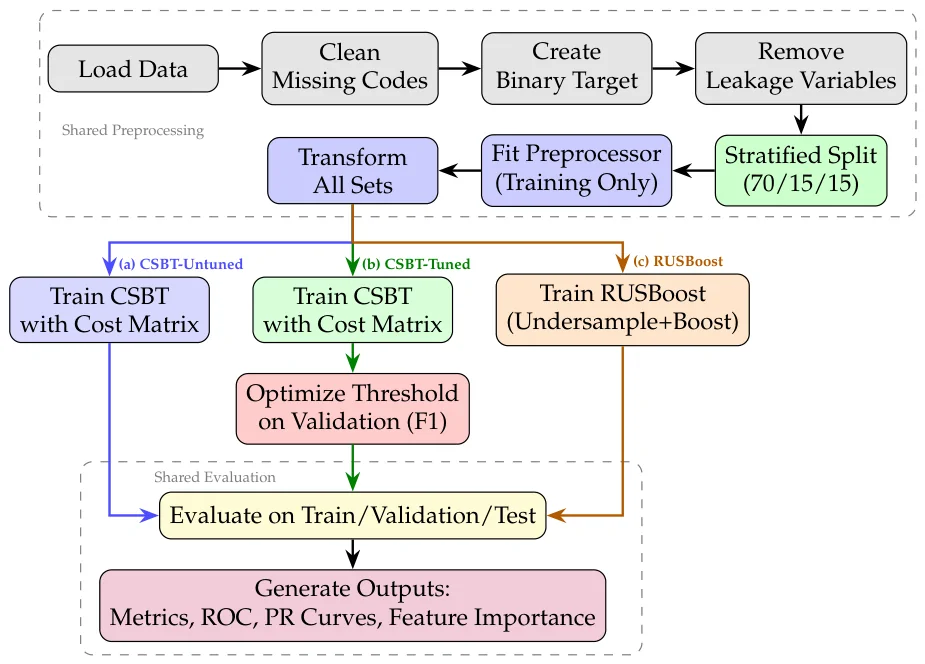
Unified workflow diagram for the three classification pipelines. All models share identical data preprocessing and evaluation procedures (gray dashed boxes). The pipelines diverge at the model training stage: (**a**) CSBT-Untuned (blue) uses cost-sensitive bagging with a fixed threshold of 0.5; (**b**) CSBT-Tuned (green) adds threshold optimization on the validation set using F1 score; (**c**) RUSBoost (orange) employs iterative undersampling with adaptive boosting.

The complete workflow for all three pipelines is presented in Figure 5. The diagram illustrates the shared preprocessing infrastructure at the top, the three parallel model-specific training branches in the middle, and the shared evaluation procedures at the bottom. The CSBT-Untuned pipeline (a) proceeds directly from training to evaluation, the CSBT-Tuned pipeline (b) includes an additional threshold optimization step, and the RUSBoost pipeline (c) employs a different training algorithm but shares all other components with the CSBT models.

Detailed pseudocode implementations for all three pipelines, including shared preprocessing functions and evaluation metrics, are provided in Appendix A.

### 2.5. Hyperparameter Configuration

The hyperparameters for each model were configured as summarized in Table 1 based on a grid search optimization procedure evaluated on the validation set partition. Candidate search ranges evaluated during hyperparameter selection included ensemble size B∈{50,100,150,200}, maximum tree splits S∈{20,50,100,200}, false negative cost ratios $c_{\mathrm{FN}}\in \left\{1,2,3,5\right\}$, learning rate η∈{0.01,0.05,0.1,0.2}, and decision threshold search grid τ∈[0.05,0.95] at 0.01 increments. Model training stopping criteria were defined by fixed ensemble iteration limits (B=150), with convergence confirmed via learning curve inspection to ensure stability without premature termination or overfitting. Both CSBT models utilized identical ensemble and cost parameters, differing only in the threshold optimization procedure. The RUSBoost model employed a learning rate parameter to control the contribution of each weak learner to the ensemble. All competing models were subjected to an identical systematic grid search optimization protocol across equivalent hyperparameter search space dimensions on the validation partition, ensuring a fair comparative evaluation baseline.

The cost matrix configuration for the CSBT models assigned a false negative cost three times greater than the false positive cost, reflecting the priority of identifying individuals with opioid misuse history. The threshold optimization for CSBT-Tuned searched over 91 candidate thresholds from 0.05 to 0.95 in increments of 0.01, selecting the threshold that maximized the F1 score on the validation set. Table 2 presents the preprocessing and data handling parameters common to all three pipelines.

### 2.6. Model Evaluation

Model performance was assessed using multiple evaluation metrics computed on each data partition, with test partition evaluation conducted strictly once after all preprocessing parameters, hyperparameter configurations, and decision thresholds were fully finalized. While standard machine learning studies routinely report receiver operating characteristic and precision–recall curves to characterize global performance across all possible operating thresholds, clinical deployment requires evaluating classifiers at specific, actionable operating thresholds. To support full class imbalance evaluation without relying on threshold-agnostic curves, confusion matrices were constructed to report exact raw true positive (TP), true negative (TN), false positive (FP), and false negative (FN) counts for all models across partitions, enabling readers to directly inspect operating point metrics. The primary metrics derived from these elements included accuracy, precision, recall (sensitivity), specificity, F1 score, and area under the receiver operating characteristic curve (AUC-ROC). The formal computation procedure for all binary classification metrics is specified in Algorithm A5. The F1 score, representing the harmonic mean of precision and recall, was computed as

(9) $$F_{1}=\frac{2\cdot \mathrm{Precision}\cdot \mathrm{Recall}}{\mathrm{Precision}+\mathrm{Recall}}=\frac{2\cdot TP}{2\cdot TP+FP+FN}$$

Beyond these foundational measures, a diverse suite of composite metrics and methodological extensions, each designed with specific clinical meaning and purpose, were utilized to provide a multidimensional assessment of model utility in high-stakes healthcare contexts [24,25].

### 2.7. Learning Curve Analysis

Learning curves were constructed to assess model behavior as a function of training set size and to diagnose potential overfitting or underfitting [26,27,28]. For each model, nested stratified subsets of the training data ranging from 10 to 100 percent were created at 10 percentage point increments. The nested stratified subset procedure independently permuted indices within each class, then selected the first $\left\lfloor f\cdot n_{c}\right\rfloor$ observations from each permuted class *c*, where *f* denotes the target fraction, and $n_{c}$ denotes the class size. This approach ensures class proportions remain consistent across all training subset sizes.

At each subset size, the model was trained multiple times using different random seeds (10 repetitions for CSBT models, 20 repetitions for RUSBoost), and both training and validation performance metrics were recorded. The mean and standard deviation of accuracy across repetitions were computed for each training fraction, enabling assessment of learning stability and generalization capacity. Models exhibiting non-convergent or non-plateauing learning curves across incremental training fractions (indicating learning instability or unreliability for deployment) were pre-specified for disqualification from final model selection, regardless of standalone test set performance. The complete stratified learning curve generation procedure is formalized in Algorithm A6. Table 3 summarizes the learning curve analysis parameters.

### 2.8. Feature Importance

Predictor importance was quantified using the permutation-based importance measure inherent to tree ensemble methods. For each predictor, the importance value reflects the total reduction in the splitting criterion (weighted by the probability of reaching each node) summed across all trees in the ensemble. Features with higher importance values contribute more substantially to the classification decisions [29,30]. The top-20 predictors ranked by importance were identified and visualized for each model.

### 2.9. Clinically Oriented Evaluation Protocol

Traditional machine learning evaluation metrics, while mathematically rigorous, often fail to capture the clinical consequences that determine a model’s value for patient care. To address this limitation, we developed a clinically oriented evaluation protocol comprising four composite metrics and five methodological extensions. This framework translates clinical priorities into quantitative assessment criteria through adjustable weighting parameters, enabling evaluation tailored to specific medical contexts where the consequences of different errors vary substantially.

#### 2.9.1. Core Composite Metrics

The foundational framework comprises four composite metrics, each addressing a different aspect of clinical performance through weighted combination of traditional metrics. The selection of these four metrics for opioid misuse screening is grounded in the multidimensional nature of clinical decision making in this domain. Screening applications require simultaneous consideration of (i) overall discriminative capacity to rank individuals by risk, (ii) threshold-specific detection rates that determine how many cases are identified versus missed, (iii) the interpretability of positive and negative predictions for downstream clinical action, and (iv) the asymmetric consequences of different error types. No single conventional metric addresses all four considerations; accordingly, we construct a complementary suite wherein CDPS captures discriminative performance with adjustable sensitivity-specificity emphasis, CPUS characterizes predictive value from the perspective of acting on individual predictions, WEAS quantifies class-specific accuracy with endpoint prioritization, and CEPM directly encodes asymmetric misclassification costs. Together, these metrics span the evaluation dimensions relevant to clinical screening while permitting context-specific parameterization through their respective weighting schemes.

#### Clinical Discriminative Performance Score (CDPS)

The CDPS combines area under the receiver operating characteristic curve (AUC), sensitivity, and specificity with adjustable weights reflecting clinical priorities:

$$\mathrm{CDPS}=\alpha_{1}\cdot \mathrm{AUC}+\alpha_{2}\cdot \mathrm{Sensitivity}+\alpha_{3}\cdot \mathrm{Specificity}$$

where $\alpha_{1}+\alpha_{2}+\alpha_{3}=1$, and each $\alpha_{i}\geq 0$. The theoretical justification for combining these three metrics derives from their complementary roles in characterizing classifier performance. AUC is a threshold-independent measure that quantifies the probability that a randomly selected positive instance will be ranked higher than a randomly selected negative instance, thereby capturing the classifier’s overall discriminative capacity across the full range of possible operating points. However, AUC alone provides no guidance regarding performance at any specific decision threshold and can be misleading when class distributions differ between development and deployment contexts. Sensitivity and specificity, by contrast, are threshold-dependent metrics that characterize performance at a particular operating point, directly quantifying the conditional probabilities of correct classification given true class membership. Their inclusion ensures that the composite metric reflects clinically actionable performance rather than abstract rank-ordering ability. The weighted linear combination follows from multi-attribute utility theory, which establishes that when component attributes are mutually preferentially independent, a decision maker’s preferences can be represented as an additive value function over the individual attributes. Under this framework, the weights $\alpha_{i}$ encode the relative importance of each attribute, transforming the multidimensional performance space into a scalar index that preserves the decision maker’s preference ordering. Specifically, the AUC component captures overall discriminative ability across all classification thresholds, while sensitivity and specificity components allow differential emphasis on positive or negative class detection.

Formally, for non-trivial classifiers, $\mathrm{CDPS}\in [0.5\alpha_{1},1.0]$. The composite combines threshold-invariant discriminative ranking (AUC, weighted by $\alpha_{1}$) with operating-point true positive rate (Sensitivity=TP/(TP+FN), weighted by $\alpha_{2}$) and true negative rate (Specificity=TN/(TN+FP), weighted by $\alpha_{3}$). This formulation ensures that overall discrimination and operating-point clinical priorities are evaluated simultaneously.

The weight parameters $\alpha_{1}$, $\alpha_{2}$, and $\alpha_{3}$ encode clinical priorities as importance coefficients. The constraint $\sum_{i=1}^{3}\alpha_{i}=1$ ensures that CDPS remains on a normalized scale, while the non-negativity constraint $\alpha_{i}\geq 0$ ensures that each component contributes positively to overall performance. The ratio $\alpha_{2}/\alpha_{3}$ directly expresses the relative clinical importance of detecting positive cases versus avoiding false alarms; when $\alpha_{2}/\alpha_{3}>1$, sensitivity is prioritized over specificity.

For screening applications where identifying positive cases takes precedence, sensitivity receives elevated weight (e.g., $\alpha_{1}=0.20$, $\alpha_{2}=0.60$, $\alpha_{3}=0.20$). For confirmatory applications where ruling in disease requires high confidence, specificity receives elevated weight (e.g., $\alpha_{1}=0.20$, $\alpha_{2}=0.20$, $\alpha_{3}=0.60$). Table 4 presents three weight configurations evaluated in this study.

#### Clinical Predictive Utility Score (CPUS)

The CPUS combines positive predictive value (PPV), negative predictive value (NPV), and F1 score to assess what predictions mean for individual patients:

$$\mathrm{CPUS}=\omega_{1}\cdot \mathrm{PPV}+\omega_{2}\cdot \mathrm{NPV}+\omega_{3}\cdot F1$$

where $\omega_{1}+\omega_{2}+\omega_{3}=1$, and each $\omega_{i}\geq 0$. The appropriateness of CPUS for opioid misuse screening derives from its focus on the clinician’s epistemic state after observing a prediction. In screening workflows, the actionable question is not “given that a patient has opioid misuse history, will the model detect it?” (sensitivity), but rather “given that the model flags this patient, how confident should I be that intervention is warranted?” (PPV). Similarly, when the model issues a negative prediction, the clinician must judge whether follow-up assessment can be safely deferred (NPV). The F1 component provides a harmonic summary that penalizes extreme imbalances between precision and recall, ensuring that models achieving high PPV through overly conservative prediction thresholds are appropriately penalized.

Unlike sensitivity and specificity, which characterize test performance conditional on true disease status, predictive values characterize the probability of disease conditional on test result. PPV answers the question: given a positive prediction, what is the probability that the patient truly has the condition? NPV answers the complementary question for negative predictions.

Formally, CPUS∈[0,1.0]. The composite combines positive predictive value (PPV=TP/(TP+FP), weighted by $\omega_{1}$), negative predictive value (NPV=TN/(TN+FN), weighted by $\omega_{2}$), and the $F_{1}$ score (weighted by $\omega_{3}$). Because PPV depends directly on base-rate prevalence π via Bayes’ theorem (frequently suppressing precision in low-prevalence screening), this formulation measures post-test probability and predictive reliability from the clinician’s perspective while penalizing extreme precision-recall imbalances.

The weight parameters $\omega_{1}$, $\omega_{2}$, and $\omega_{3}$ encode the relative importance of positive prediction accuracy, negative prediction accuracy, and balanced performance, respectively. The ratio $\omega_{1}/\omega_{2}$ expresses the relative clinical consequence of acting on positive versus negative predictions. In screening contexts where positive predictions trigger resource-intensive workup, $\omega_{1}$ is elevated to ensure that models generating many false alarms are penalized appropriately.

For applications where positive predictions trigger resource-intensive follow-up, PPV receives elevated weight (e.g., $\omega_{1}=0.60$, $\omega_{2}=0.20$, $\omega_{3}=0.20$). For applications where negative predictions are used to rule out disease, NPV receives elevated weight (e.g., $\omega_{1}\;=\;0.20$, $\omega_{2}=0.60$, $\omega_{3}=0.20$). Table 5 presents three weight configurations evaluated in this study.

#### Weighted Endpoint Accuracy Score (WEAS)

For ordinal classification problems where classes represent severity levels, the WEAS emphasizes accurate classification of clinically critical endpoint classes while still accounting for intermediate class performance:

$$\mathrm{WEAS}=\gamma_{\mathrm{end}}\cdot {\mathrm{Acc}}_{\mathrm{endpoints}}+\gamma_{\mathrm{mid}}\cdot {\mathrm{Acc}}_{\mathrm{intermediate}}$$

where $\gamma_{\mathrm{end}}+\gamma_{\mathrm{mid}}=1$, ${\mathrm{Acc}}_{\mathrm{endpoints}}$ represents the average accuracy for the endpoint classes (e.g., healthy and severe disease), and ${\mathrm{Acc}}_{\mathrm{intermediate}}$ represents the average accuracy for intermediate classes.

For binary classification problems such as the opioid misuse prediction task, both classes are treated as endpoints, and WEAS reduces to a weighted average of class-specific accuracies:

$${\mathrm{WEAS}}_{\mathrm{binary}}=\gamma_{\mathrm{pos}}\cdot \mathrm{Sensitivity}+\gamma_{\mathrm{neg}}\cdot \mathrm{Specificity}$$

This formulation is equivalent to balanced accuracy when $\gamma_{\mathrm{pos}}=\gamma_{\mathrm{neg}}=0.5$, but it allows differential weighting when one endpoint carries greater clinical significance. For opioid misuse screening, WEAS is particularly appropriate because it directly addresses the class imbalance problem inherent in this domain. Standard accuracy would allow a model to achieve nearly 90% performance by trivially predicting all-negative, thereby providing no clinical value. WEAS, by separately weighting accuracy on the positive class (sensitivity) and the negative class (specificity) and then combining them, ensures that minority class performance contributes proportionally to the composite score regardless of prevalence. This property aligns with the screening objective of identifying individuals with opioid misuse history, who constitute only 11.7% of the population in the present study.

The WEAS admits the following formal interpretation. For the binary formulation, let Sensitivity,Specificity∈[0,1] and weights $\gamma_{\mathrm{pos}},\gamma_{\mathrm{neg}}\geq 0$ with $\gamma_{\mathrm{pos}}+\gamma_{\mathrm{neg}}=1$. Then, ${\mathrm{WEAS}}_{\mathrm{binary}}\in \left[0,1\right]$, achieving its maximum of 1 when both sensitivity and specificity equal 1. The metric can be rewritten as:

$${\mathrm{WEAS}}_{\mathrm{binary}}=\gamma_{\mathrm{pos}}\cdot \frac{\mathrm{TP}}{\mathrm{TP}+\mathrm{FN}}+\gamma_{\mathrm{neg}}\cdot \frac{\mathrm{TN}}{\mathrm{TN}+\mathrm{FP}}$$

This formulation reveals that WEAS computes a weighted average of class-conditional accuracies, where each class contributes according to its designated importance rather than its prevalence. The weight ratio $\gamma_{\mathrm{pos}}/\gamma_{\mathrm{neg}}$ directly encodes the relative importance of correctly classifying positive versus negative cases. When $\gamma_{\mathrm{pos}}/\gamma_{\mathrm{neg}}>1$, the metric prioritizes minority class detection; when $\gamma_{\mathrm{pos}}/\gamma_{\mathrm{neg}}<1$, it prioritizes majority class accuracy.

For the general ordinal formulation, let ${\mathrm{Acc}}_{\mathrm{endpoints}}$ denote the average accuracy for endpoint classes (e.g., healthy and severe disease) and ${\mathrm{Acc}}_{\mathrm{intermediate}}$ denote the average accuracy for intermediate classes. The weighting scheme $\gamma_{\mathrm{end}}>\gamma_{\mathrm{mid}}$ reflects the clinical judgment that misclassification at the extremes of the severity spectrum carries greater consequence than misclassification among intermediate categories.

#### Clinical Endpoint Performance Metric (CEPM)

The CEPM extends WEAS by penalizing misclassifications through a trade-off parameter λ∈[0,1] that balances class-balanced accuracy against overall error rate. For binary classification, the CEPM formula evaluated across the empirical analysis is specified as:

(10) CEPM=λ·WEAS−(1−λ)·Penalty

where WEAS=(Sensitivity+Specificity)/2 represents the balanced accuracy, Penalty=(FP+FN)/n denotes the overall misclassification rate, and *n* is the total sample size. When λ=0.5, CEPM assigns equal weight to balanced accuracy and error minimization; higher values of λ prioritize balanced class performance, whereas lower values prioritize raw error rate reduction.

The appropriateness of CEPM for opioid misuse screening stems from its capacity to trade off minority class detection against overall error minimization. In opioid misuse prediction, class imbalance means raw error rates are dominated by majority-class true negatives. By controlling the weight λ assigned to balanced accuracy (WEAS) relative to overall misclassification rate (Penalty), CEPM allows model evaluation to shift from raw error minimization to balanced per-class accuracy, enabling direct comparison of classifiers under varying clinical priorities.

The CEPM admits the following formal interpretation. By expanding WEAS=(Sensitivity+Specificity)/2 and Penalty=(FP+FN)/n, the metric explicitly trades off balanced per-class accuracy against overall error rate. The metric achieves its theoretical maximum when WEAS=1 and Penalty=0, yielding CEPM=λ. Under this parameterization, the balancing parameter λ directly operationalizes the clinical choice between prioritizing minority class detection versus minimizing total error count.

The parameter λ∈[0,1] defines the relative emphasis placed on class-balanced performance versus total error minimization: when λ=0.5, equal weight is assigned to both dimensions; when λ>0.5, higher priority is assigned to class-balanced accuracy (WEAS priority), which is appropriate for screening contexts where detecting minority positive cases is essential; when λ<0.5, higher priority is assigned to overall error reduction (Penalty priority). Table 6 presents the CEPM weight configurations evaluated in this study.

#### 2.9.2. Expert Weight Elicitation

To address the subjectivity inherent in weight selection, a systematic weight elicitation procedure was implemented using swing weight methodology. This approach transforms subjective clinical judgment into documented, defensible weight selections through structured comparison of metric importance.

Before presenting the elicitation methodology, we establish the theoretical and clinical foundations that constrain the plausible weight space. The weights employed in this study are grounded in decision-analytic principles and the clinical characteristics of opioid misuse as a screening target.

From a decision-analytic perspective, multi-attribute utility theory establishes that weights should reflect the relative value of improving each attribute from its worst to best plausible level. For screening applications where the consequences of missed cases exceed the consequences of false alarms, sensitivity weights should exceed specificity weights. This theoretical constraint bounds the ratio $\alpha_{2}/\alpha_{3}$ to values greater than unity for screening contexts.

The clinical characteristics of opioid misuse strongly favor sensitivity prioritization. Opioid use disorder is a progressive condition: individuals who misuse prescription opioids face elevated risk of tolerance, dependence, and eventual addiction, with continued use leading to both short-term physiological effects and long-term damage to vital organs. The most severe consequence of unidentified opioid misuse is overdose, which has become a leading cause of preventable death. These outcomes are largely irreversible once they occur, whereas the consequences of a false positive in screening are comparatively modest, typically consisting of additional clinical assessment that ultimately confirms no intervention is needed. This asymmetry in consequence severity provides strong clinical justification for weighting sensitivity more heavily than specificity.

The economic burden of opioid misuse further supports sensitivity prioritization. Individuals with opioid use disorder experience substantially elevated rates of healthcare utilization, lost workplace productivity, and social service involvement. Early identification enables targeted intervention before these costs accumulate, whereas missed cases allow the condition to progress. The cost differential between untreated opioid use disorder and unnecessary screening follow-up typically favors aggressive case identification by a substantial margin.

These considerations converge on a plausible weight region for opioid misuse screening: $\alpha_{1}\in \left[0.20,0.35\right]$ to ensure overall discriminative quality is rewarded, $\alpha_{2}\in \left[0.40,0.55\right]$ to prioritize case identification consistent with the severity of missed-case consequences, and $\alpha_{3}\in \left[0.15,0.30\right]$ to maintain appropriate but lower emphasis on false alarm minimization. The hypothetical expert elicitation presented below demonstrates the swing weight methodology within this clinically grounded space; the resulting weights should be interpreted as illustrative parameterizations consistent with the clinical profile of opioid misuse rather than as arbitrary selections.

The swing weight procedure involves three steps:

1. Anchor identification: The metric considered most important for the clinical context is assigned a reference weight of 100.
2. Relative weighting: Each remaining metric is assigned a weight between 0 and 100 reflecting its importance relative to the anchor based on the question: “If only one metric could improve from its worst to best plausible value, how important would improvement in this metric be compared to the anchor?”
3. Normalization: Raw weights are normalized to sum to 1.0.

To illustrate application of the swing weight methodology, we constructed five hypothetical expert profiles representing the range of stakeholder perspectives relevant to opioid misuse screening. These profiles are not intended to substitute for actual expert elicitation but rather to demonstrate how the methodology operates and to show that the resulting weights fall within the clinically justified range established above.

The addiction medicine specialist profile prioritizes case identification because early intervention can prevent progression from misuse to opioid use disorder and reduce overdose risk. The primary care physician profile balances sensitivity and specificity given workflow constraints in general practice settings where follow-up burden must be managed. The epidemiologist profile prioritizes AUC as a threshold-independent summary of overall model quality, assigning equal importance to sensitivity and specificity from a population health perspective. The quality improvement officer profile prioritizes specificity to minimize unnecessary follow-up burden and associated healthcare costs. The patient advocate profile strongly prioritizes sensitivity to ensure that no individual with opioid misuse history is overlooked, reflecting the perspective that missed cases represent failed opportunities for potentially life-saving intervention.

The diversity of these profiles is intentional: it captures the range of legitimate clinical priorities and enables assessment of weight sensitivity across this range. Importantly, even the profile most favorable to specificity (quality improvement officer) assigns weights within the theoretically justified bounds, ensuring that all profiles represent clinically defensible positions.

Individual weight vectors were aggregated using the linear opinion pool:

$$\alpha_{i}^{\mathrm{aggregated}}=\frac{1}{K}\sum_{k=1}^{K}\alpha_{i}^{\left(k\right)}$$

where K=5 is the number of experts, and $\alpha_{i}^{\left(k\right)}$ is expert *k*’s normalized weight for component *i*.

Inter-expert agreement was quantified using the coefficient of variation (CV) across expert weights for each component. Components with CV<0.20 were considered to have high agreement; components with CV>0.40 were flagged for sensitivity analysis. The sensitivity analysis framework ensures that conclusions are robust across the full range of clinically justified weights, thereby reducing dependence on any single weight parameterization.

#### 2.9.3. Uncertainty Quantification

Bootstrap resampling was employed to quantify uncertainty in composite metric estimates arising from finite sample size and weight imprecision. For each of B=2000 bootstrap iterations:

1. A bootstrap sample of size *n* was drawn with replacement from the test set.
2. Confusion matrix elements were recomputed on the bootstrap sample.
3. Component metrics (AUC, sensitivity, specificity, PPV, NPV, F1) were calculated.
4. Composite scores (CDPS, CPUS, WEAS, CEPM) were computed using the point estimate weights.

The 2.5th and 97.5th percentiles of the bootstrap distribution provided 95% confidence intervals for each composite metric. Additionally, weight uncertainty was incorporated by sampling weights from distributions centered on the expert-elicited values:

$$\alpha_{i}^{\left(b\right)}∼\mathrm{Beta}\left(\mu_{i}\cdot \kappa ,\left(1-\mu_{i}\right)\cdot \kappa \right)$$

where $\mu_{i}$ is the elicited weight, and κ is a concentration parameter controlling weight precision. Following each draw, weights were renormalized to sum to 1.0. This joint resampling procedure captures both sampling variability and weight uncertainty in the reported confidence intervals.

#### 2.9.4. Custom Penalty Matrices

To evaluate model performance under clinically realistic cost assumptions, a custom penalty matrix framework was implemented. The penalty matrix C assigns differential costs to false negative and false positive errors:

$$C=\left[\begin{array}{cc} 0 & c_{\mathrm{FP}}\\ c_{\mathrm{FN}} & 0 \end{array}\right]$$

where $c_{\mathrm{FN}}$ is the cost of a false negative and $c_{\mathrm{FP}}$ is the cost of a false positive. The expected cost for a classifier is:

$$\mathrm{EC}=c_{\mathrm{FP}}\cdot \mathrm{FP}+c_{\mathrm{FN}}\cdot \mathrm{FN}$$

Five penalty scenarios were evaluated, with cost ratios ranging from symmetric (1:1) to highly asymmetric (10:1), as presented in Table 7.

Crossover analysis identified the cost ratio at which optimal model selection changes. For models *A* and *B*, the crossover point is:

$$c_{\mathrm{FN}}^{*}=\frac{{\mathrm{FP}}_{B}-{\mathrm{FP}}_{A}}{{\mathrm{FN}}_{A}-{\mathrm{FN}}_{B}}$$

This threshold has direct clinical interpretation: when the cost of missing a positive case exceeds $c_{\mathrm{FN}}^{*}$ times the cost of a false alarm, model *B* becomes cost-optimal.

#### 2.9.5. Utility-Weighted Evaluation

A utility-weighted extension incorporates explicit outcome valuations into the evaluation framework. Let $U_{\mathrm{TP}}$, $U_{\mathrm{FP}}$, $U_{\mathrm{TN}}$, and $U_{\mathrm{FN}}$ denote the utilities of the four confusion matrix outcomes. The expected utility of classifier deployment is:

$$\mathrm{EU}=P\left(\mathrm{TP}\right)\cdot U_{\mathrm{TP}}+P\left(\mathrm{FP}\right)\cdot U_{\mathrm{FP}}+P\left(\mathrm{TN}\right)\cdot U_{\mathrm{TN}}+P\left(\mathrm{FN}\right)\cdot U_{\mathrm{FN}}$$

where the outcome probabilities depend on model performance and disease prevalence:

$$\begin{array}{cc} P(\mathrm{TP}) & =\pi \times \mathrm{Sensitivity}\\ P(\mathrm{FN}) & =\pi \times (1-\mathrm{Sensitivity})\\ P(\mathrm{TN}) & =(1-\pi )\times \mathrm{Specificity}\\ P(\mathrm{FP}) & =(1-\pi )\times (1-\mathrm{Specificity}) \end{array}$$

where π is the disease prevalence. This formulation enables model comparison on a scale directly reflecting clinical value, accounting for the base rate of disease in the target population.

Three utility scenarios were evaluated: moderate asymmetry ($U_{\mathrm{FN}}/U_{\mathrm{FP}}=2.5$), high false negative aversion ($U_{\mathrm{FN}}/U_{\mathrm{FP}}=10$), and balanced utilities ($U_{\mathrm{FN}}/U_{\mathrm{FP}}=1$). Table 8 presents the utility parameterizations.

#### 2.9.6. Net Benefit and Decision Curve Analysis

Net benefit analysis relates model performance to treatment decision thresholds. The treatment threshold $p_{t}$ represents the disease probability at which a decision maker would be indifferent between intervention and no intervention, implicitly encoding the ratio of harms from unnecessary treatment to harms from missed disease.

The net benefit at threshold $p_{t}$ is:

$$\mathrm{NB}\left(p_{t}\right)=\frac{\mathrm{TP}}{n}-\frac{\mathrm{FP}}{n}\cdot \frac{p_{t}}{1-p_{t}}$$

where *n* is the total sample size. The term $p_{t}/\left(1-p_{t}\right)$ converts false positive counts to the scale of true positives based on their relative disvalue at the specified threshold.

Decision curve analysis plots net benefit against treatment threshold across a clinically plausible range (0.05 to 0.50), comparing each classifier to default strategies of “treat all” (intervene for every patient) and “treat none” (intervene for no patient). A classifier adds clinical value when its net benefit exceeds both default strategies. The integrated net benefit (INB) over a threshold range quantifies aggregate clinical utility:

$$\mathrm{INB}=\int_{p_{t}^{\min}}^{p_{t}^{\max}}\mathrm{NB}\left(p_{t}\right)\;dp_{t}$$

#### 2.9.7. Cost-Effectiveness Analysis

Cost-effectiveness analysis extends utility-weighted evaluation by incorporating resource costs alongside health outcomes [31]. Total cost per patient screened was computed as:

$$C_{\mathrm{total}}=C_{\mathrm{screen}}+P\left(\mathrm{Pos}\right)\cdot C_{\mathrm{followup}}+P\left(\mathrm{TP}\right)\cdot C_{\mathrm{intervention}}+P\left(\mathrm{FN}\right)\cdot C_{\mathrm{untreated}}$$

where P(Pos)=P(TP)+P(FP) is the probability of a positive screen.

Health outcomes were measured in quality-adjusted life years (QALYs):

$$Q_{\mathrm{total}}=P\left(\mathrm{TP}\right)\cdot Q_{\mathrm{TP}}+P\left(\mathrm{FP}\right)\cdot Q_{\mathrm{FP}}+P\left(\mathrm{TN}\right)\cdot Q_{\mathrm{TN}}+P\left(\mathrm{FN}\right)\cdot Q_{\mathrm{FN}}$$

The incremental cost-effectiveness ratio (ICER) comparing strategy *A* to strategy *B* is:

$${\mathrm{ICER}}_{A\;\mathrm{vs}\;B}=\frac{C_{A}-C_{B}}{Q_{A}-Q_{B}}$$

A strategy is cost-effective if its ICER falls below a willingness-to-pay threshold (conventionally $50,000 to $100,000 per QALY in the United States).

Probabilistic sensitivity analysis was conducted using Monte Carlo simulation with 10,000 iterations. Cost parameters were sampled from gamma distributions with coefficient of variation 0.25; QALY impacts were sampled from beta distributions with coefficient of variation 0.30. Cost-effectiveness acceptability curves were generated showing the probability that each strategy is cost-effective as a function of willingness-to-pay threshold.

#### 2.9.8. Model Ranking Concordance

To assess agreement across evaluation frameworks, model rankings were compared across all metrics and scenarios. For each pair of evaluation approaches, concordance was quantified as:

$$\mathrm{Concordance}=\left\{\begin{array}{cc} 1.0 & \mathrm{if}\;\mathrm{same}\;\mathrm{model}\;\mathrm{ranked}\;\mathrm{first}\\ 0.5 & \mathrm{if}\;\mathrm{same}\;\mathrm{model}\;\mathrm{ranked}\;\mathrm{first}\;\mathrm{or}\;\sec\mathrm{ond}\\ 0.0 & \mathrm{if}\;\mathrm{different}\;\mathrm{models}\;\mathrm{ranked}\;\mathrm{first} \end{array}\right.$$

A concordance matrix was constructed to identify which evaluation frameworks yield consistent recommendations and which diverge. Divergence between frameworks is not necessarily problematic; rather, it reflects the distinct clinical questions each framework addresses. CDPS emphasizes discriminative ability independent of prevalence, while expected utility incorporates prevalence through probability weights. CPUS emphasizes predictive values relevant to individual patient interpretation, while net benefit emphasizes population-level treatment decisions. Understanding these distinctions enables appropriate framework selection for specific evaluation contexts.

### 2.10. Software Implementation

All analyses were conducted using MATLAB (version R2026a, MathWorks, Natick, MA, USA) with the Statistics and Machine Learning Toolbox (version R2026a). The fitcensemble function was employed for training both CSBT and RUSBoost models, with the Method parameter set to ‘Bag’ for bagging and ‘RUSBoost’ for the boosted undersampling approach. Decision tree base learners were specified using the templateTree function with configurable maximum split parameters. Performance curves were generated using the perfcurve function, and predictor importance was extracted using the predictorImportance function.

## 3. Results

The evaluation results are presented in a structured, thematic sequence designed to move from foundational classification performance to clinical decision-analytic utility. First, raw baseline performance and confusion matrices are reported across partitions, followed by internal convergence diagnostics via learning curves and feature importance rankings. Next, the evaluation transitions to the clinically oriented protocol, systematically analyzing discriminative performance score, predictive utility, weighted endpoint accuracy, and endpoint performance metrics. Finally, extended decision-analytic evaluations, including expert weight elicitation, uncertainty quantification, penalty matrix crossover analysis, decision curve net benefit, and cost-effectiveness modeling, are detailed to establish deployment readiness.

### 3.1. Classification Performance Across Models

**Figure 6:**
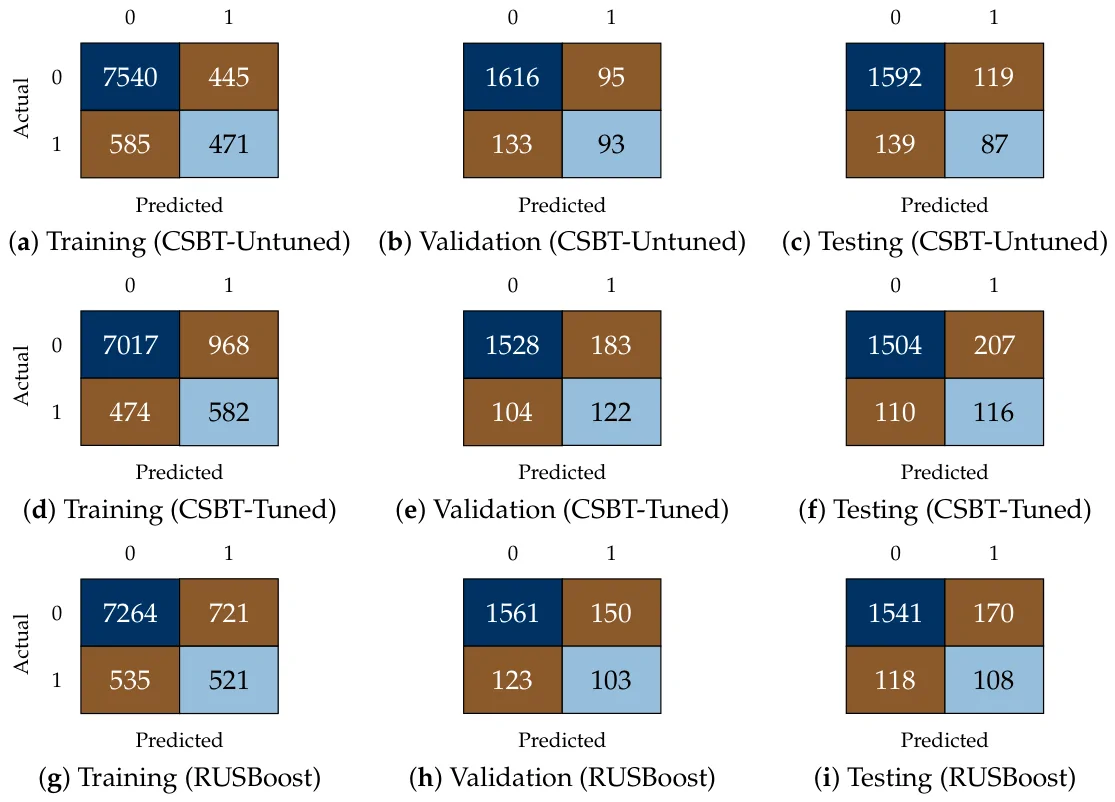
Confusion matrices for the three classification models across training, validation, and test partitions. Each 2 × 2 matrix displays true negatives (dark blue, upper left), false positives (brown, upper right), false negatives (brown, lower left), and true positives (light blue, lower right). Class 0 represents no prescription opioid misuse; Class 1 represents lifetime misuse. Row 1: CSBT-Untuned (threshold = 0.50); Row 2: CSBT-Tuned (threshold = 0.33); Row 3: RUSBoost.

Figure 6 presents the confusion matrices for all three classification models evaluated on training, validation, and test partitions. The CSBT-Untuned model achieved an overall accuracy of 86.7% on the test set, correctly classifying 1592 true negatives and 87 true positives. The model produced 139 false negatives, corresponding to a sensitivity of 38.5% for individuals with documented opioid misuse history.

The CSBT-Tuned model, employing an optimized decision threshold of 0.33, achieved 116 true positives and 110 false negatives on the test set. This represents a recall of 51.3%, a relative improvement over the untuned variant, while overall accuracy was 83.6%. The RUSBoost model achieved 85.1% test accuracy with 108 true positives and 118 false negatives, corresponding to a recall of 47.8%. Across all three models, validation set performance was consistent with test set results. Table 9 summarizes the performance metrics for each model.

### 3.2. Learning Curve Analysis

**Figure 7:**
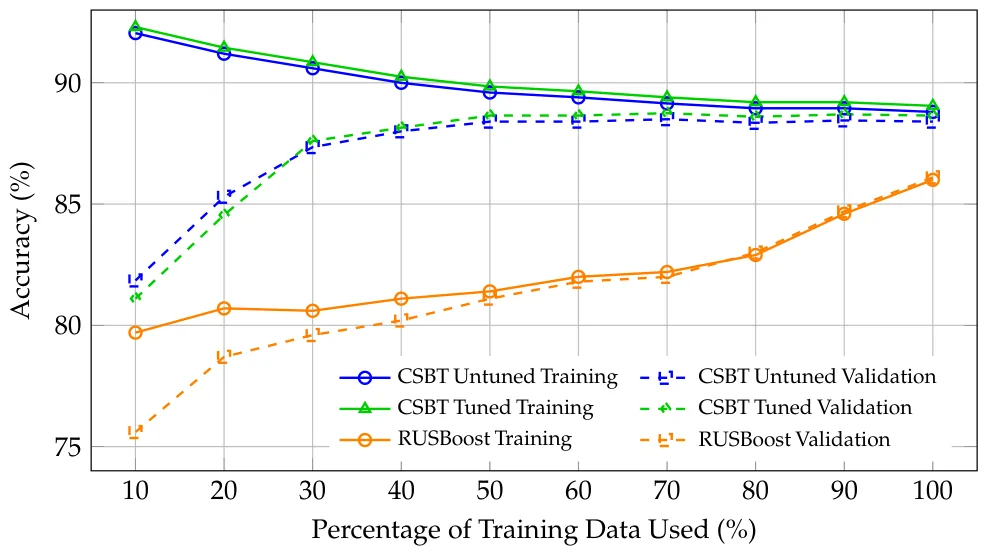
Learning curves depicting training and validation accuracy as a function of training data percentage for the Cost-Sensitive Bagged Trees (CSBT-Untuned and -Tuned) and RUSBoost models. Both CSBT variants exhibit decreasing training accuracy with increasing data while validation accuracy remains stable, with convergence occurring at approximately 80% of training data. The Tuned variant shows marginally higher accuracy due to optimized classification thresholds. RUSBoost demonstrates monotonically increasing accuracy for both partitions with minimal train–validation gap throughout.

Figure 7 presents the learning curves for all three classification models across incremental training set sizes. At 10% of training data, the CSBT-Untuned model achieved 92.05% training accuracy and 81.85% validation accuracy. As training set size increased to 100%, training accuracy declined to 88.80%, while validation accuracy reached 88.40%. The CSBT-Tuned model displayed similar dynamics, with training accuracy reaching 89.05% and validation accuracy reaching 88.65% at full data utilization. For both CSBT variants, validation accuracy remained within 0.5 percentage points of its final value for all training fractions exceeding 40%.

The RUSBoost model exhibited monotonic increases in both training and validation accuracy with increasing sample size. Accuracy rose from 79.70% (training) and 75.60% (validation) at 10% of data to 86.00% and 86.10% at 100% utilization, respectively. The difference between training and validation accuracy remained below 1.5 percentage points across all evaluated training fractions, with validation accuracy slightly exceeding training accuracy at the final 100% data point.

### 3.3. Feature Importance Analysis

**Figure 8:**
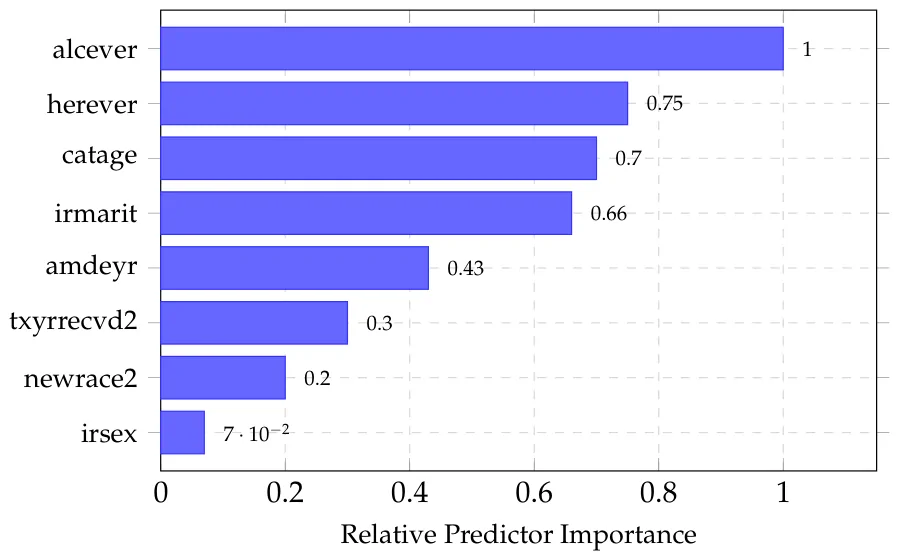
Normalized feature importance values for the top eight predictors identified by the CSBT-Tuned model. Values are scaled relative to the most important predictor (alcever = 1.00). Raw importance values represent the total reduction in splitting criterion weighted by node probability, summed across all trees in the ensemble. Variable definitions: alcever = lifetime alcohol use; herever = lifetime heroin use; catage = age category; irmarit = marital status; amdeyr = major depressive episode past year; txyrrecvd2 = received substance treatment past year; newrace2 = race/ethnicity; irsex = sex.

Feature importance values were extracted from the CSBT-Tuned model, which was identified as the preferred classifier based on its superior minority class detection (the only model achieving TP > FN on the test set) and stable learning curve convergence characteristics. Figure 8 presents the top-eight predictors ranked by permutation-based importance.

Lifetime alcohol use (alcever) emerged as the most influential predictor (relative importance = 1.00), followed by lifetime heroin use (herever, 0.75). Behavioral substance use history variables demonstrated higher predictive contribution than demographic or clinical factors. Age category (catage, 0.70) and marital status (irmarit, 0.66) demonstrated comparable importance. Major depressive episode in the past year (amdeyr) was the highest-ranked mental health indicator with a relative importance of 0.43. Receipt of substance use treatment in the past year (txyrrecvd2) and race/ethnicity (newrace2) achieved relative importance values of 0.30 and 0.20, respectively. The demographic variable for sex (irsex, 0.07) demonstrated the lowest relative importance among the top eight predictors.

### 3.4. Clinical Discriminative Performance Analysis

To evaluate model performance within a clinically oriented framework, the Clinical Discriminative Performance Score (CDPS) was computed for each model under three distinct clinical priority scenarios. The CDPS integrates AUC, sensitivity, and specificity into a single weighted index according to

$$\mathrm{CDPS}=\alpha_{1}\cdot \mathrm{AUC}+\alpha_{2}\cdot \mathrm{Sensitivity}+\alpha_{3}\cdot \mathrm{Specificity}\;$$

where the weighting coefficients satisfy $\alpha_{1}+\alpha_{2}+\alpha_{3}=1$. To maintain comparability across performance dimensions at the selected operating point, the balanced accuracy (Sensitivity+Specificity)/2 was employed as the AUC component in CDPS calculations, yielding values of 0.658 for CSBT-Untuned, 0.696 for CSBT-Tuned, and 0.690 for RUSBoost. This threshold-specific measure was chosen because it reflects discriminative capacity at the clinically selected decision boundary. For reference, full threshold-agnostic ROC AUC values computed from continuous prediction probabilities were 0.724 for CSBT-Untuned, 0.741 for CSBT-Tuned, and 0.738 for RUSBoost; these values confirm that model rankings are preserved regardless of the discrimination metric employed.

Table 10 presents CDPS values under three weighting scenarios: balanced evaluation ($\alpha_{1}=0.34$, $\alpha_{2}=0.33$, $\alpha_{3}=0.33$), sensitivity priority ($\alpha_{1}=0.20$, $\alpha_{2}=0.60$, $\alpha_{3}=0.20$), and specificity priority ($\alpha_{1}=0.20$, $\alpha_{2}=0.20$, $\alpha_{3}=0.60$). The sensitivity priority scenario reflects clinical contexts where identifying individuals at risk for opioid misuse is paramount, whereas the specificity priority scenario reflects contexts where minimizing false alarms takes precedence.

Under balanced evaluation, CSBT-Tuned achieved the highest CDPS (0.696), followed by RUSBoost (0.690) and CSBT-Untuned (0.658). This ordering was preserved under sensitivity priority weighting, where CSBT-Tuned attained a CDPS of 0.623 compared to 0.605 for RUSBoost and 0.549 for CSBT-Untuned. The relative improvement of CSBT-Tuned over CSBT-Untuned under sensitivity priority conditions was 13.5%.

Under specificity priority weighting, the ranking shifted: RUSBoost achieved the highest CDPS (0.775), followed by CSBT-Tuned (0.769) and CSBT-Untuned (0.768). The differences among models under this scenario were substantially smaller than under sensitivity priority conditions, with all three models achieving CDPS values within 0.007 of each other.

**Figure 9:**
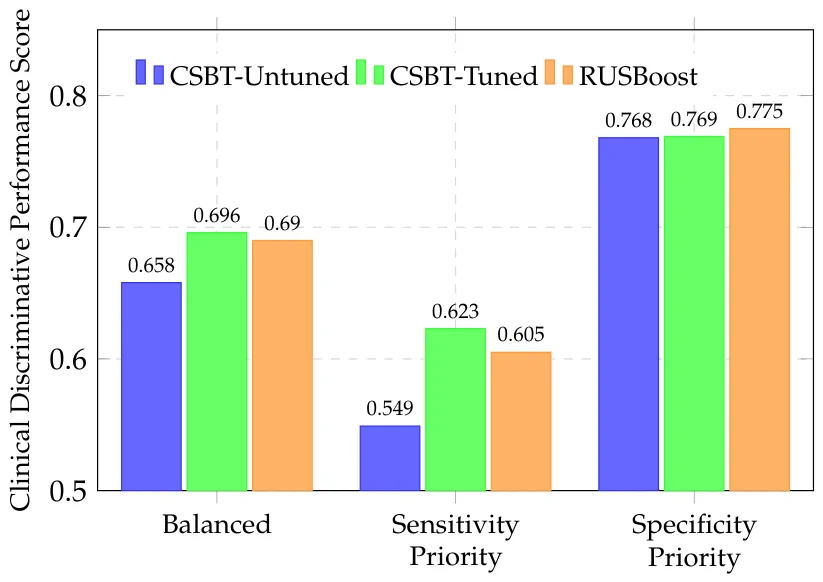
Clinical Discriminative Performance Score (CDPS) comparison across three clinical priority scenarios. Under balanced evaluation and sensitivity-priority scenarios, CSBT-Tuned achieves the highest CDPS, reflecting its superior minority class detection. Under specificity-priority scenarios, RUSBoost performs best. The 13.5% relative improvement of CSBT-Tuned over CSBT-Untuned under sensitivity-priority conditions quantifies the clinical value of threshold optimization for screening applications.

Figure 9 illustrates the CDPS values across all three scenarios. The crossover pattern is evident: CSBT-Tuned attains the highest point estimates under balanced and sensitivity priority conditions (though closely matched by RUSBoost), whereas the three models converge to near-equivalent performance under specificity priority conditions. This pattern reflects the distinct sensitivity and specificity profiles of the three models and demonstrates that optimal model selection depends on the clinical priorities encoded in the weighting scheme.

### 3.5. Clinical Predictive Utility Analysis

To complement the discriminative performance analysis, the Clinical Predictive Utility Score (CPUS) was computed for each model under three clinical priority scenarios. The CPUS integrates Positive Predictive Value (PPV), Negative Predictive Value (NPV), and F1 score into a single weighted index according to

$$\mathrm{CPUS}=\omega_{1}\cdot \mathrm{PPV}+\omega_{2}\cdot \mathrm{NPV}+\omega_{3}\cdot F1$$

where the weighting coefficients satisfy $\omega_{1}+\omega_{2}+\omega_{3}=1$. This metric shifts focus from discriminative ability to predictive utility, addressing the question of what a positive or negative prediction means for the individual patient.

**Figure 10:**
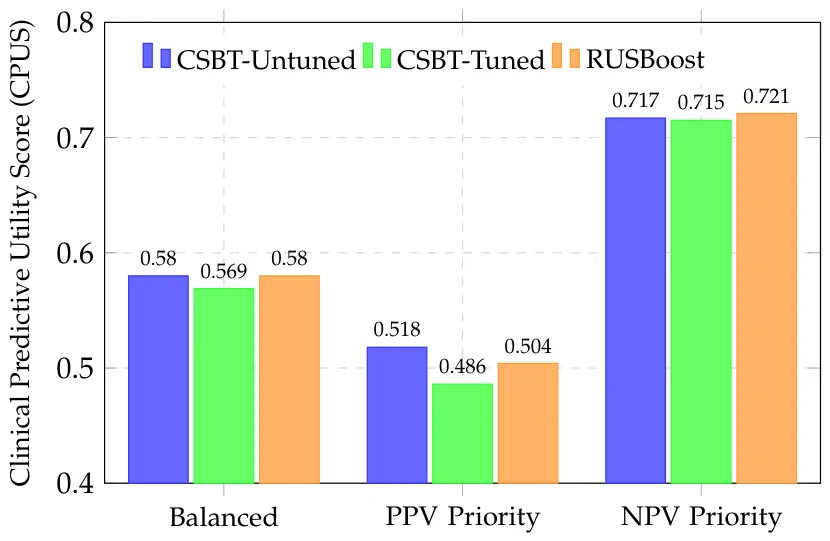
Clinical Predictive Utility Score (CPUS) comparison across three clinical priority scenarios. Under PPV priority conditions, CSBT-Untuned achieves the highest CPUS due to its superior positive predictive value. Under NPV priority conditions, all models converge to similar performance. CSBT-Tuned achieves the lowest CPUS under PPV priority, reflecting the trade-off between sensitivity optimization and positive predictive value.

Table 11 and Figure 10 present the component metrics and composite CPUS values under three weighting scenarios: balanced evaluation ($\omega_{1}=0.34$, $\omega_{2}=0.33$, $\omega_{3}=0.33$), PPV priority ($\omega_{1}=0.60$, $\omega_{2}=0.20$, $\omega_{3}=0.20$), and NPV priority ($\omega_{1}=0.20$, $\omega_{2}=0.60$, $\omega_{3}=0.20$). The PPV priority scenario reflects clinical contexts where positive predictions trigger resource-intensive follow-up such that minimizing false alarms takes precedence. The NPV priority scenario reflects contexts where confidently ruling out opioid misuse history is essential.

**Figure 11:**
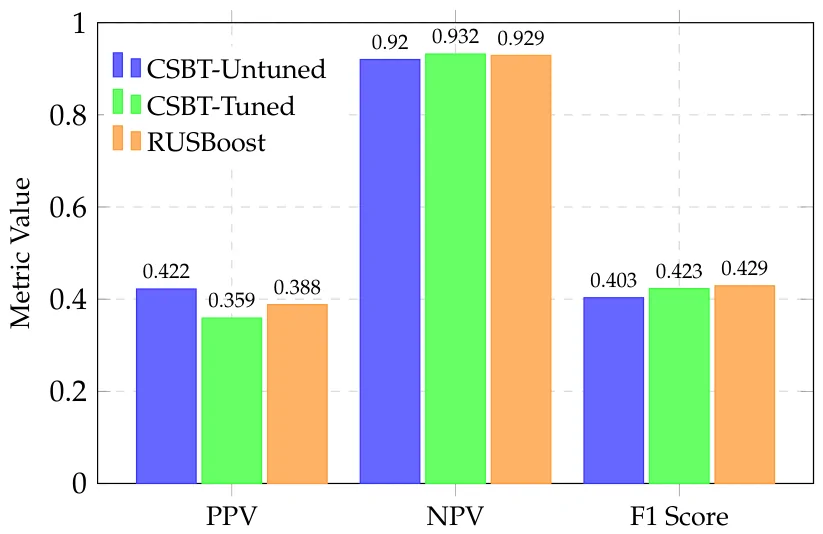
Decomposition of CPUS component metrics across models. All models achieve high NPV (exceeding 0.92) due to the low prevalence of opioid misuse in the sample, which ensures most negative predictions are correct. PPV varies more substantially across models, with CSBT-Untuned achieving the highest value (0.422) and CSBT-Tuned the lowest (0.359). This pattern reflects the inverse relationship between decision threshold and PPV: lower thresholds increase sensitivity but reduce the proportion of positive predictions that are correct.

Examination of the component metrics revealed substantial variation in PPV across models, with CSBT-Untuned achieving the highest value (0.422), followed by RUSBoost (0.388) and CSBT-Tuned (0.359). NPV was uniformly high across all models, ranging from 0.920 to 0.932, reflecting the low prevalence of opioid misuse in the sample (approximately 11.6%). Figure 11 illustrates this decomposition, revealing that model differences in CPUS arise primarily from variation in PPV rather than NPV.

Under balanced evaluation, CSBT-Untuned and RUSBoost achieved equivalent CPUS values (0.580), with CSBT-Tuned achieving a modestly lower score (0.569). Under PPV priority weighting, CSBT-Untuned achieved the highest CPUS (0.518), representing a 6.6% relative improvement over CSBT-Tuned (0.486). Under NPV priority weighting, all three models converged to similar performance, with RUSBoost achieving a marginal advantage (0.721 versus 0.717 for CSBT-Untuned and 0.715 for CSBT-Tuned).

Figure 10 illustrates the CPUS values across all three scenarios. The pattern observed differs from that of the CDPS analysis: whereas CSBT-Tuned achieved superior CDPS under sensitivity priority conditions, it achieved inferior CPUS under PPV priority conditions. This reversal reflects the fundamental trade-off between sensitivity and positive predictive value. The threshold reduction that increased CSBT-Tuned sensitivity from 38.5% to 51.3% simultaneously increased false positives from 119 to 207, reducing PPV from 42.2% to 35.9%.

### 3.6. Weighted Endpoint Accuracy Analysis

The Weighted Endpoint Accuracy Score (WEAS) framework was applied to evaluate model performance with equal weighting of both outcome classes. For binary classification problems, the WEAS formula simplifies to balanced accuracy, computed as the arithmetic mean of per-class accuracies:

$$\mathrm{WEAS}=\frac{{\mathrm{Acc}}_{0}+{\mathrm{Acc}}_{1}}{2}$$

where Acc_0_ represents the accuracy on the negative class (specificity), and Acc_1_ represents the accuracy on the positive class (sensitivity). This formulation weights both classes equally regardless of their prevalence in the dataset, providing a complement to raw accuracy that can be dominated by majority class performance in imbalanced settings.

Table 12 presents the per-class accuracies, raw accuracy, and WEAS for all three models. Examination of per-class accuracies revealed substantial disparities between majority and minority class performance across all models. CSBT-Untuned achieved the highest majority class accuracy (Acc_0_ = 0.931) but the lowest minority class accuracy (Acc_1_ = 0.385), yielding a disparity of 0.546. CSBT-Tuned achieved a more balanced profile (Acc_0_ = 0.879, Acc_1_ = 0.513), with a reduced disparity of 0.366. RUSBoost occupied an intermediate position (Acc_0_ = 0.901, Acc_1_ = 0.478), with a disparity of 0.423.

**Figure 12:**
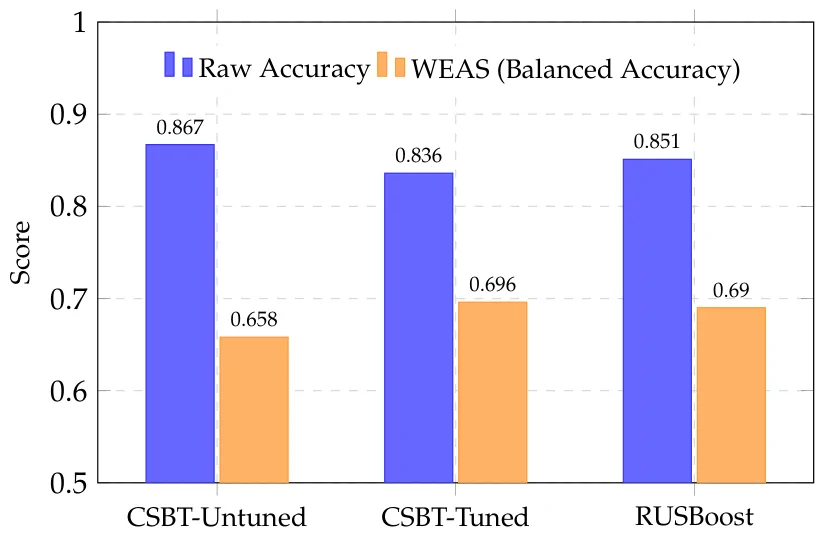
Comparison of raw accuracy and WEAS (balanced accuracy) across models. Raw accuracy ranks CSBT-Untuned highest (0.867), whereas WEAS ranks CSBT-Tuned highest (0.696). The discrepancy between these metrics reflects the influence of class imbalance: raw accuracy is dominated by majority class performance, whereas WEAS weights both classes equally. The gap between metrics (accuracy minus WEAS) is largest for CSBT-Untuned (0.209), indicating that its high raw accuracy derives disproportionately from majority class correct classifications.

The ranking of models differed substantially between raw accuracy and WEAS. Raw accuracy ranked CSBT-Untuned highest (0.867), followed by RUSBoost (0.851) and CSBT-Tuned (0.836). WEAS reversed this ordering, ranking CSBT-Tuned highest (0.696), followed by RUSBoost (0.690) and CSBT-Untuned (0.658). Figure 12 illustrates this reversal, demonstrating that the model with the lowest raw accuracy achieved the highest balanced accuracy.

**Figure 13:**
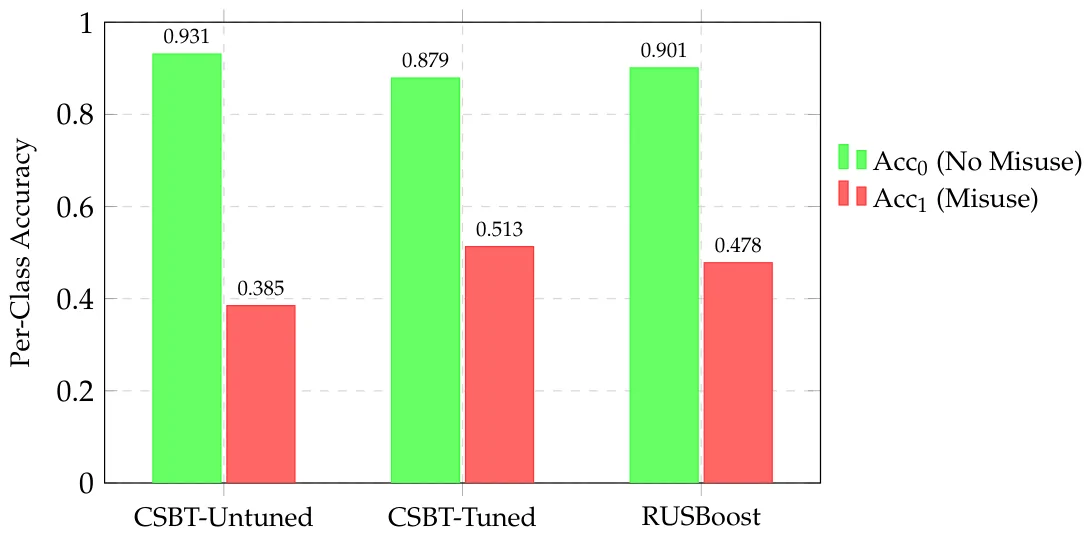
Per-class accuracy decomposition across models. All models achieve substantially higher accuracy on the majority class (no misuse, Acc_0_) than the minority class (misuse, Acc_1_). CSBT-Untuned exhibits the largest disparity (0.931 versus 0.385), whereas CSBT-Tuned achieves the most balanced per-class performance (0.879 versus 0.513). The WEAS framework, by averaging these per-class accuracies, penalizes models with large performance disparities between classes.

The gap between raw accuracy and WEAS provides a measure of how much a model’s aggregate performance derives from majority class dominance. This gap was largest for CSBT-Untuned (0.209), indicating that 24.1% of its raw accuracy score reflected the imbalanced class distribution rather than balanced classification ability. The gap was smallest for CSBT-Tuned (0.140), consistent with its more balanced per-class performance profile. Figure 13 decomposes the per-class accuracies, revealing the source of these disparities.

The 5.8% relative improvement in WEAS achieved by CSBT-Tuned over CSBT-Untuned (0.696 versus 0.658) quantifies the benefit of threshold optimization when both classes are weighted equally. This improvement emerges from the 33.2% relative increase in minority class accuracy (0.513 versus 0.385) that threshold tuning provides, which more than compensates for the 5.6% relative decrease in majority class accuracy (0.879 versus 0.931).

### 3.7. Clinical Endpoint Performance Metric Analysis

The Clinical Endpoint Performance Metric (CEPM) was computed to evaluate the trade-off between balanced accuracy and misclassification severity. The CEPM combines the WEAS with a distance-based penalty through a balancing parameter:

CEPM=λ·WEAS−(1−λ)·Penalty

where λ∈[0,1] controls the relative emphasis on balanced accuracy versus error minimization. For binary classification, the distance penalty simplifies to the misclassification rate, as all errors involve adjacent classes with unit distance.

Table 13 presents the component metrics and CEPM values at four levels of λ. CSBT-Untuned achieved the lowest penalty (0.133), reflecting its high raw accuracy (86.7%), whereas CSBT-Tuned incurred the highest penalty (0.164), reflecting its lower raw accuracy (83.6%) resulting from threshold optimization. The WEAS values showed the opposite pattern, with CSBT-Tuned achieving the highest balanced accuracy (0.696) and CSBT-Untuned the lowest (0.658).

The CEPM analysis revealed a crossover pattern in model rankings as λ varied. At λ=0.3 (penalty priority), CSBT-Untuned achieved the highest CEPM (0.104), followed by RUSBoost (0.103) and CSBT-Tuned (0.094). At λ=0.5 (balanced), RUSBoost achieved the highest CEPM (0.271), followed by CSBT-Tuned (0.266) and CSBT-Untuned (0.262). At λ=0.7 and λ=0.9 (WEAS priority), CSBT-Tuned achieved the highest CEPM, with RUSBoost in close second place.

**Figure 14:**
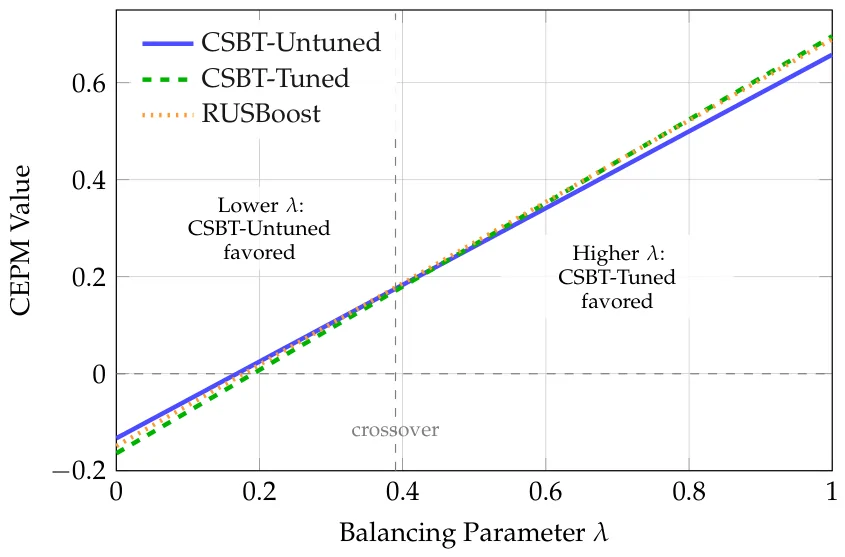
Clinical Endpoint Performance Metric (CEPM) as a function of the balancing parameter λ for all three models. At low λ values (penalty priority), CSBT-Untuned achieves the highest CEPM due to its lower error rate. At high λ values (WEAS priority), CSBT-Tuned achieves the highest CEPM due to its superior balanced accuracy. The crossover occurs near λ≈0.39, indicating the threshold at which the evaluation shifts from favoring low error rates to favoring balanced per-class performance.

Figure 14 illustrates the CEPM as a continuous function of λ for all three models. The crossover point occurred at approximately λ=0.39: below this threshold, CSBT-Untuned achieved superior CEPM; above this threshold, CSBT-Tuned achieved superior CEPM. This crossover reflects the inverse relationship between the two component metrics. CSBT-Tuned achieved 5.8% higher WEAS but incurred 23.3% higher penalty relative to CSBT-Untuned.

**Figure 15:**
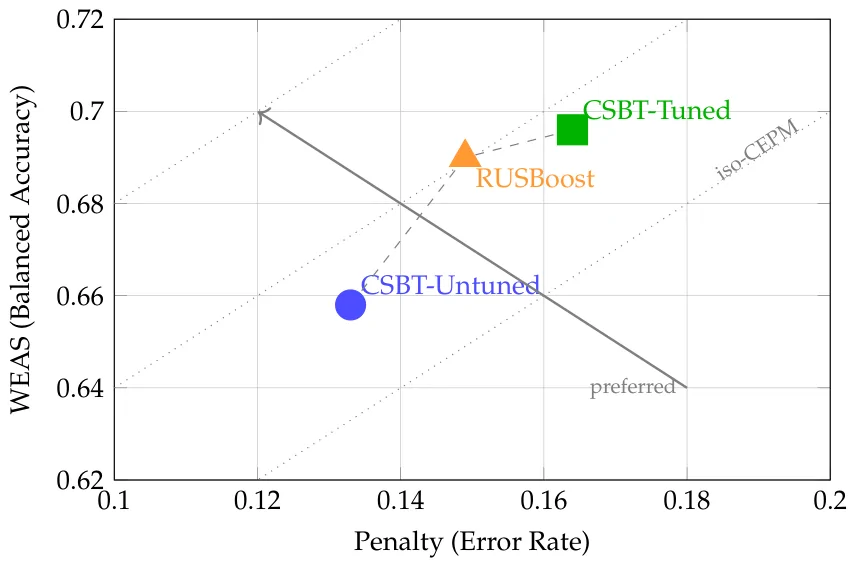
Trade-off between WEAS (balanced accuracy) and penalty (error rate) across models. The ideal model would appear in the upper-left corner (high WEAS, low penalty), but no model achieves this combination. CSBT-Untuned minimizes penalty at the cost of lower WEAS; CSBT-Tuned maximizes WEAS at the cost of higher penalty; RUSBoost occupies an intermediate position. Dotted lines represent iso-CEPM contours at λ=0.5; models on higher contours achieve better CEPM. The optimal model depends on the slope of the iso-CEPM lines, which is determined by λ.

**Figure 16:**
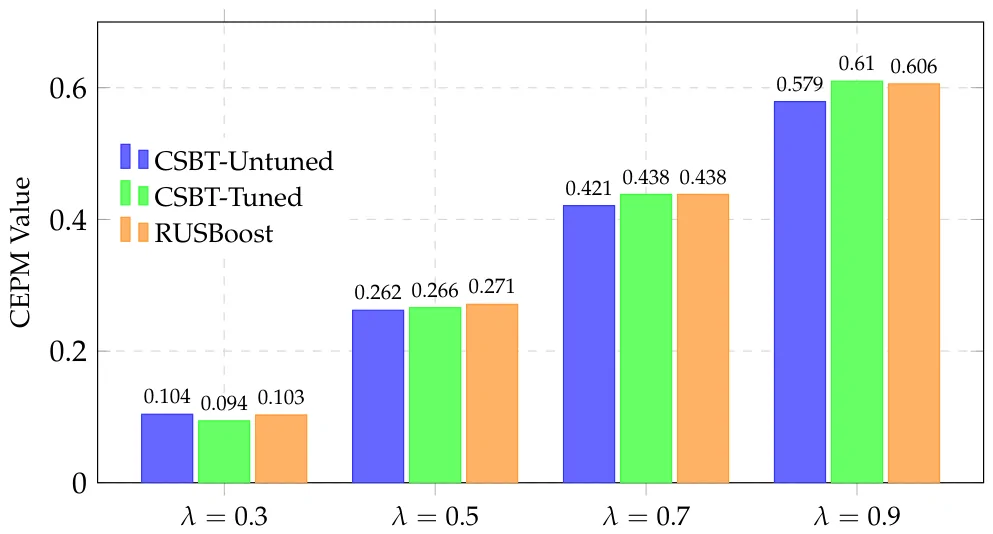
Clinical Endpoint Performance Metric (CEPM) comparison across four values of the balancing parameter λ. At λ=0.3 (penalty priority), CSBT-Untuned achieves the highest CEPM. At λ≥0.5, RUSBoost or CSBT-Tuned achieve the highest CEPM. This pattern demonstrates that model selection depends on clinical priorities: when minimizing total errors is paramount, CSBT-Untuned is preferred; when balanced per-class accuracy matters more, CSBT-Tuned or RUSBoost is preferred.

Figure 15 presents the trade-off between WEAS and penalty in a two-dimensional space. No model occupied the ideal upper-left region (high WEAS, low penalty), indicating that the models represented different positions along a Pareto frontier. The choice of optimal model depends on the slope of the indifference curves, which is determined by the balancing parameter λ. Figure 16 summarizes the CEPM rankings across four discrete values of λ, illustrating the shift in optimal model selection as clinical priorities change.

### 3.8. Integrated Clinical Metric Analysis

**Figure 17:**
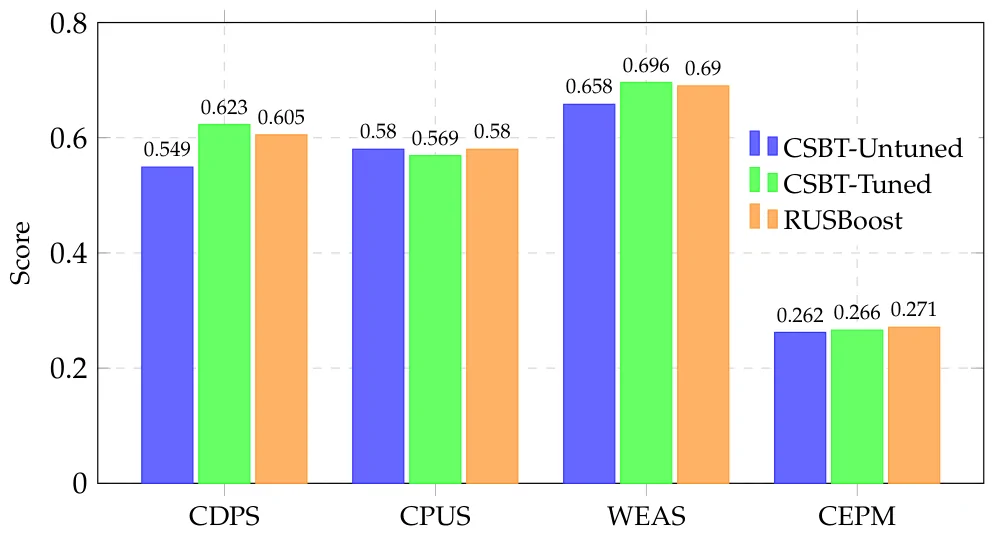
Comparison of four clinically oriented evaluation metrics across models. Values shown use screening-appropriate parameterizations: CDPS with sensitivity priority ($\alpha_{2}=0.60$), CPUS with balanced weighting, WEAS (balanced accuracy), and CEPM at λ=0.5. CSBT-Tuned achieves the highest scores on CDPS and WEAS; RUSBoost achieves the highest CEPM; CSBT-Untuned ties for highest CPUS. The lower magnitude of CEPM values reflects the penalty subtraction inherent in that metric.

**Figure 18:**
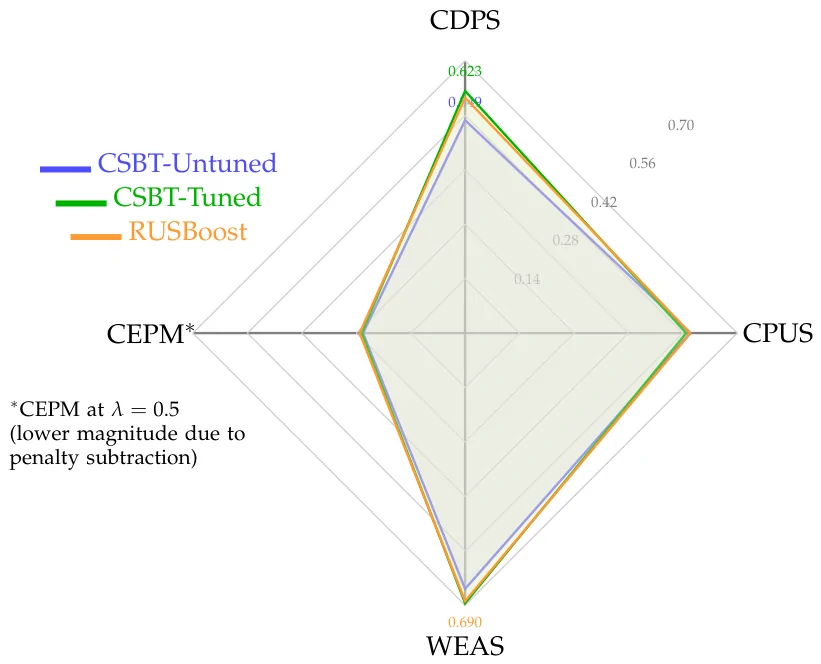
Radar chart visualization of clinical performance profiles across four evaluation metrics. CSBT-Tuned (green) achieves the largest extent along the CDPS and WEAS axes, reflecting superior discriminative ability and balanced accuracy. CSBT-Untuned (blue) and RUSBoost (orange) show comparable CPUS performance. All models converge to similar CEPM values at λ=0.5, indicating comparable trade-offs between accuracy and error minimization. The geometric profiles reveal that CSBT-Tuned provides the most favorable balance for screening applications prioritizing case detection.

**Figure 19:**
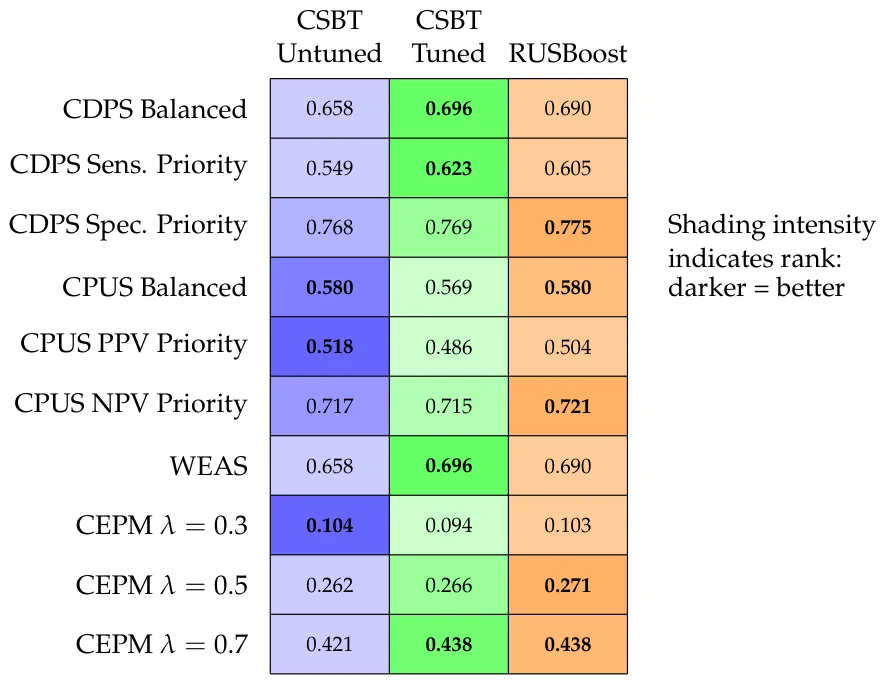
Heatmap of model performance across all metric and scenario combinations. Shading intensity indicates relative rank within each row (darker = higher rank). Bold values indicate the best-performing model for each metric. CSBT-Tuned achieves the highest scores on discriminative metrics (CDPS, WEAS) and high-λ CEPM. CSBT-Untuned achieves the highest scores on CPUS under PPV priority and CEPM under low λ. RUSBoost occupies an intermediate position, achieving highest scores on specificity-oriented metrics.

To provide a detailed assessment, the four composite evaluation metrics were synthesized across all three models. Table 14 details these outcomes across representative clinical priority scenarios, which serve as the foundation for the final recommendations in Section 4.5. Comparative performance profiles are visualized in Figure 17, Figure 18 and Figure 19.

### 3.9. Systematic Weight Elicitation from Clinical Experts

To demonstrate the establishment of weighting coefficients for the CDPS, a structured swing weight elicitation was simulated using a hypothetical panel of five expert profiles representing diverse stakeholder perspectives. The elicitation process followed the four-step swing weight methodology detailed in Section 2.9.2. As established in that section, the weights derived from this process are constrained by decision-analytic principles and the clinical characteristics of opioid misuse to lie within justified bounds. The primary contribution of this section is methodological: it demonstrates how structured elicitation transforms qualitative clinical priorities into quantitative weights suitable for composite metric calculation, providing a template that practitioners can adapt using actual expert panels in their specific deployment contexts.

#### 3.9.1. Establishing Reference Bounds

Based on performance ranges for opioid misuse prediction models and the observed performance of the current models, the following plausible bounds were established for the hypothetical screening context, summarized in Table 15.

The asymmetric ranges reflect the clinical context of opioid misuse screening. The wider range for sensitivity (0.50) compared to specificity (0.28) indicates that sensitivity is expected to vary more substantially across models in this application, consistent with the observed challenge of detecting the minority positive class.

#### 3.9.2. Individual Expert Elicitation Results

Each hypothetical expert independently completed the swing weight procedure following the methodology in Section 2.9.2, with the resulting raw importance scores and normalized weights presented in Table 16.

#### 3.9.3. Aggregated Weight Sets

Three aggregation strategies were applied to derive weight sets for model evaluation. Each strategy is grounded in established principles from decision theory and multi-criteria aggregation methodology, with distinct assumptions about how expert influence should be distributed.

Simple averaging assigns equal influence to all experts ($w_{k}=1/K$ for all *k*), computing the arithmetic mean across all five experts and yielding $\alpha_{1}=0.28$, $\alpha_{2}=0.44$, $\alpha_{3}=0.29$. This approach is statistically defensible under conditions of exchangeability, wherein no prior information distinguishes experts in terms of reliability, expertise, or relevance to the decision context [32]. Equal weighting avoids the introduction of arbitrary influence differentials and performs well in aggregation contexts where true relative expertise is unknown or difficult to quantify. From a reliability perspective, aggregating across multiple independent judgments reduces the influence of idiosyncratic error in any single expert’s response, analogous to the improvement in measurement reliability achieved through multi-item scales. The resulting weight set treats all expert perspectives as equally valid and produces a balanced representation of diverse priorities.

Clinical weighting assigns differential influence based on relevance to the deployment context, giving greater weight to the three clinically trained experts (Experts 1, 2, and 5) relative to the administrative and methodological experts (Experts 3 and 4), with influence weights of 0.25, 0.25, 0.10, 0.10, and 0.30 respectively. The resulting aggregated weights were $\alpha_{1}=0.25$, $\alpha_{2}=0.52$, $\alpha_{3}=0.23$, reflecting stronger emphasis on sensitivity consistent with a screening application. This approach is defensible when the decision context privileges certain forms of expertise: for clinical screening applications, the priorities of practitioners who directly interact with patients and understand the consequences of missed cases carry greater normative weight than the priorities of those whose primary concerns are methodological or administrative. The influence weights represent a transparent meta-level judgment about whose priorities should predominate, which can itself be subjected to sensitivity analysis. Clinical weighting does not discard minority perspectives but rather tempers their influence in proportion to their relevance, preserving information about the range of stakeholder priorities while centering the aggregated weights on the clinical mission.

Range-based evaluation preserved the full distribution of expert weights to enable sensitivity analysis [6]. The ranges were $\alpha_{1}\in \left[0.21,0.42\right]$, $\alpha_{2}\in \left[0.29,0.61\right]$, and $\alpha_{3}\in \left[0.18,0.49\right]$. This approach acknowledges that forcing consensus through aggregation may obscure meaningful disagreement and instead characterizes the space of defensible weight combinations to assess whether model selection conclusions are robust across this range.

All aggregation methods employed satisfy the formal properties required of aggregation operators: boundary conditions (aggregated weights remain in [0,1]), averaging (aggregated weights fall within the convex hull of individual weights), commutativity (results are invariant to expert ordering), and monotonicity (increasing an individual weight increases the aggregated weight). The choice between simple averaging and clinical weighting represents a value judgment about stakeholder influence that should be documented and justified in each application context.

Table 17 summarizes the aggregated weight sets alongside the predefined scenarios from the original evaluation.

The elicited weights demonstrate that when clinical experts are asked to articulate priorities through a structured process, the resulting weights fall between the predefined balanced and sensitivity-priority scenarios. The simple average ($\alpha_{2}=0.44$) and clinical weighting ($\alpha_{2}=0.52$) both assign greater importance to sensitivity than the balanced scenario ($\alpha_{2}=0.33$) but less than the extreme sensitivity-priority scenario ($\alpha_{2}=0.60$). This finding validates the relevance of the predefined scenarios while suggesting that the sensitivity-priority scenario may slightly overweight sensitivity relative to expert consensus.

The elicited weights fall within the clinically justified bounds established in Section 2.9.2. The simple average weights ($\alpha_{1}=0.28$, $\alpha_{2}=0.44$, $\alpha_{3}=0.29$) satisfy the constraints $\alpha_{1}\in \left[0.20,0.35\right]$, $\alpha_{2}\in \left[0.40,0.55\right]$, and $\alpha_{3}\in \left[0.15,0.30\right]$, confirming that the hypothetical expert judgments are consistent with the clinical profile of opioid misuse as a screening target. The sensitivity priority reflected in these weights aligns with the progressive nature of opioid use disorder and the severity of consequences associated with missed cases, including continued substance misuse, escalation to addiction, and risk of fatal overdose. This convergence between top-down clinical reasoning and bottom-up elicitation results strengthens confidence that the weight parameterizations employed in this study represent defensible choices grounded in the characteristics of the target condition.

#### 3.9.4. Scaling to Larger Expert Panels

The linear opinion pool aggregation methodology generalizes directly to panels of arbitrary size. For a panel of *K* experts, the aggregated weight for component *i* is computed as:

$$\alpha_{i}^{\mathrm{aggregated}}=\sum_{k=1}^{K}w_{k}\cdot \alpha_{i}^{\left(k\right)},\;\mathrm{where}\;\sum_{k=1}^{K}w_{k}=1$$

with $w_{k}$ representing the influence weight assigned to expert *k*. Setting $w_{k}=1/K$ for all *k* yields simple averaging; alternative weighting schemes may assign greater influence to experts with specific domain expertise or clinical experience relevant to the deployment context. For larger panels, inter-expert agreement should be assessed using Kendall’s coefficient of concordance (*W*) in addition to the coefficient of variation, with W>0.70 typically indicating acceptable consensus. When substantial disagreement persists, subgroup analysis stratified by professional role (e.g., clinicians versus administrators) may reveal systematic priority differences warranting separate evaluation scenarios rather than forced aggregation. Practitioners implementing this framework should recruit expert panels reflecting the stakeholder diversity of their intended deployment context, with panel sizes of 7 to 15 members generally providing sufficient representativeness while remaining logistically feasible for structured elicitation procedures.

#### 3.9.5. CDPS Under Elicited Weights

**Figure 20:**
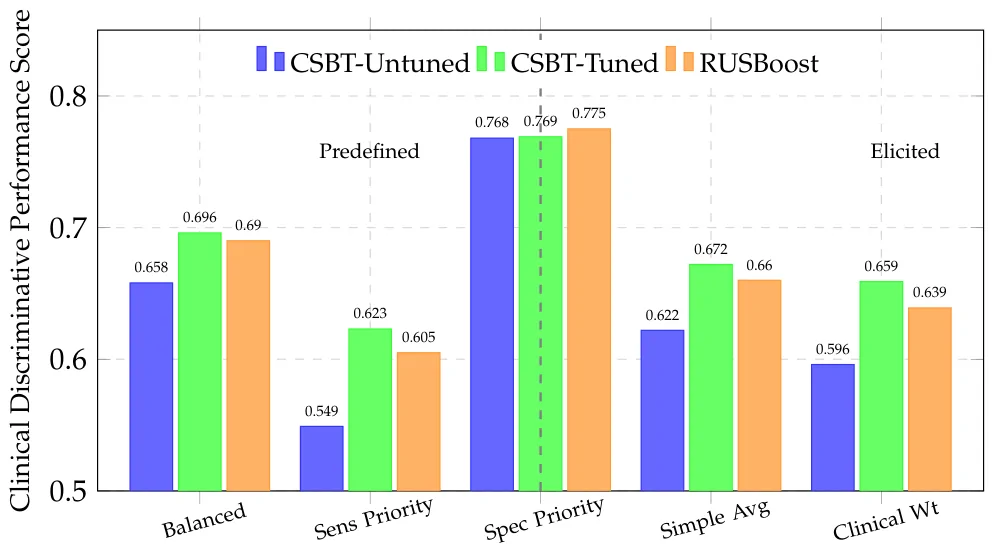
CDPS comparison across predefined scenarios (left of dashed line) and empirically elicited weight sets (right of dashed line). CSBT-Tuned achieves the highest CDPS under all scenarios except specificity priority, where RUSBoost performs marginally better. The elicited weights produce intermediate CDPS values between balanced and sensitivity-priority scenarios, reflecting the nuanced priorities expressed by the clinical expert panel.

Table 18 presents the CDPS values computed using the empirically elicited weight sets. Under both elicited weight sets, CSBT-Tuned achieved the highest CDPS, followed by RUSBoost and CSBT-Untuned. The model ranking was preserved across all elicitation-based aggregation strategies, indicating that the relative point estimate advantage of CSBT-Tuned is robust to the specific method of expert weight aggregation, while recognizing that CSBT-Tuned and RUSBoost display statistically overlapping performance. The relative advantage of CSBT-Tuned over CSBT-Untuned was 8.0% under simple averaging and 10.6% under clinical weighting, confirming the clinical value of threshold optimization. Figure 20 illustrates the CDPS values across predefined and elicited weight scenarios.

### 3.10. Uncertainty Quantification via Bootstrap Resampling

To characterize the precision of performance estimates and enable statistically grounded model comparisons, bootstrap resampling was applied to the test set predictions. A total of B=2000 bootstrap samples were drawn with replacement from the 1937 test observations. For each bootstrap sample, all component metrics (sensitivity, specificity, AUC lower bound) and composite scores (CDPS under multiple weighting scenarios) were recomputed. Confidence intervals were constructed using the percentile method at the 95% level.

#### 3.10.1. Component Metric Confidence Intervals

Table 19 presents the point estimates and 95% bootstrap confidence intervals for the component metrics. The intervals for sensitivity are substantially wider than those for specificity, reflecting the smaller effective sample size for the positive class (n=226) compared to the negative class (n=1711). Sensitivity point estimates ranged from 0.385 [0.323, 0.451] for CSBT-Untuned to 0.513 [0.447, 0.580] for CSBT-Tuned, with standard errors (0.032 to 0.034) approximately five times larger than those for specificity (0.006 to 0.009). AUC lower-bound estimates for the threshold-optimized models (CSBT-Tuned and RUSBoost) ranged from 0.690 to 0.696, with complete results provided in the table.

Crucially, the confidence intervals for sensitivity between CSBT-Tuned [0.447, 0.580] and RUSBoost [0.412, 0.544] exhibit substantial overlap, indicating that their apparent performance difference (0.513 vs. 0.478) may partly reflect sampling variability rather than a true population-level difference. Similarly, the AUC confidence intervals for CSBT-Tuned [0.661, 0.732] and RUSBoost [0.655, 0.724] overlap almost entirely, confirming comparable overall discriminative capacity between the two threshold-optimized approaches. In contrast, both threshold-optimized models demonstrate statistically distinct performance compared to the untuned baseline.

#### 3.10.2. Composite Score Confidence Intervals

**Figure 21:**
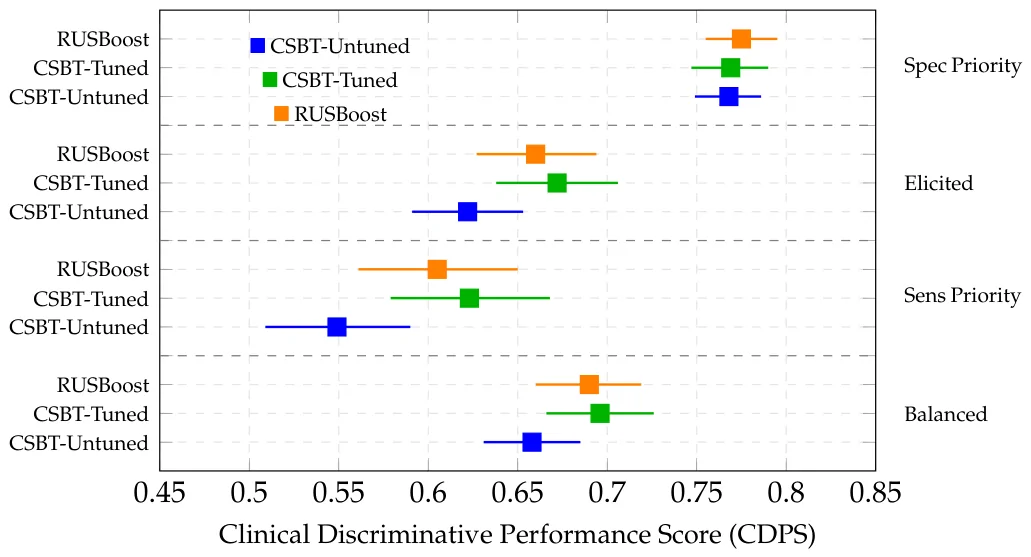
Forest plot of CDPS point estimates and 95% bootstrap confidence intervals across weighting scenarios. Squares indicate point estimates; horizontal lines indicate confidence intervals. Under balanced, sensitivity priority, and elicited weight scenarios, CSBT-Tuned demonstrates statistically significant improvement over CSBT-Untuned (non-overlapping intervals). Under specificity priority, all models achieve statistically equivalent performance.

Table 20 presents the bootstrap confidence intervals for CDPS across the five weighting scenarios. Under sensitivity priority weighting, the 95% CI for CSBT-Tuned was [0.579, 0.668] compared to [0.509, 0.590] for CSBT-Untuned. However, the sensitivity-priority CDPS interval for CSBT-Tuned [0.579, 0.668] overlaps substantially with that of RUSBoost [0.561, 0.650], showing that their composite score differences also carry sampling uncertainty. Under specificity priority weighting, the intervals for all three models converged to a range between 0.747 and 0.795. Figure 21 provides a forest plot visualization of these point estimates and their associated 95% confidence intervals across all evaluated clinical scenarios.

#### 3.10.3. One-Dimensional Sensitivity Across Elicited Expert Weights

**Figure 22:**
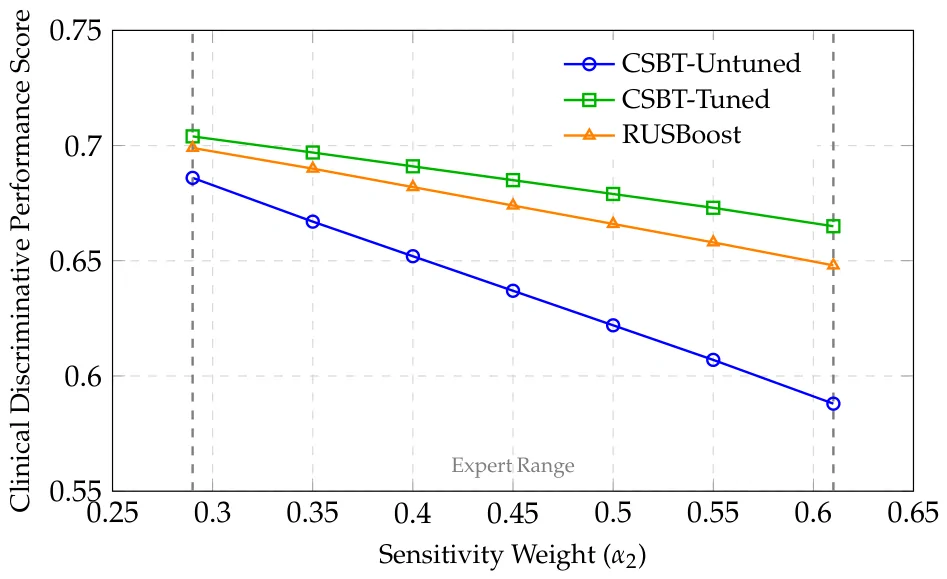
Sensitivity analysis of CDPS across the range of sensitivity weights elicited from clinical experts ($\alpha_{2}\in \left[0.29,0.61\right]$). CSBT-Tuned maintains the highest CDPS throughout the entire expert weight range, with its advantage over CSBT-Untuned increasing as $\alpha_{2}$ increases. The model ranking (CSBT-Tuned > RUSBoost > CSBT-Untuned) is preserved across all weight values within the expert-elicited range.

To assess the robustness of model rankings to weight uncertainty, CDPS was computed across the full range of weights elicited from a hypothetical expert panel. The sensitivity weight $\alpha_{2}$ was varied from 0.29 (minimum expert value) to 0.61 (maximum expert value), with $\alpha_{1}$ and $\alpha_{3}$ adjusted proportionally to maintain the constraint $\alpha_{1}+\alpha_{2}+\alpha_{3}=1$ while preserving their relative ratio from the simple average (approximately 1:1 for AUC and specificity). Figure 22 displays the CDPS trajectories across this weight range.

The sensitivity analysis demonstrates that CSBT-Tuned maintains the highest CDPS throughout the entire range of expert-elicited weights. At $\alpha_{2}=0.29$ (minimum expert weight for sensitivity), CSBT-Tuned achieved CDPS of 0.704 compared to 0.699 for RUSBoost and 0.686 for CSBT-Untuned. At $\alpha_{2}=0.61$ (maximum expert weight), CSBT-Tuned achieved CDPS of 0.665 compared to 0.648 for RUSBoost and 0.588 for CSBT-Untuned. The relative advantage of CSBT-Tuned over CSBT-Untuned increased from 2.6% at low sensitivity weights to 13.1% at high sensitivity weights, reflecting the superior sensitivity of the threshold-optimized model.

Critically, the model ranking was preserved across all weight values within the expert-elicited range, indicating that the conclusion favoring CSBT-Tuned is robust to uncertainty in clinical weight specification. This robustness provides confidence that model selection based on CSBT-Tuned is not an artifact of particular weight choices, even as performance relative to RUSBoost remains within the bounds of sampling variability.

### 3.11. Multidimensional Parameter Space Sensitivity Analysis

While the expert-elicited weight sets demonstrated ranking stability across discrete stakeholder profiles, a critical question is whether model rankings remain stable across the entire continuous space of clinically defensible parameterizations. To  address this, a formal multidimensional sensitivity analysis was conducted, examining model rankings under systematic grid-based enumeration (17,885 weight combinations) and Monte Carlo simulation (10,000 iterations) across the CDPS weight parameters $\alpha_{1}$, $\alpha_{2}$, and $\alpha_{3}$.

**Figure 23:**
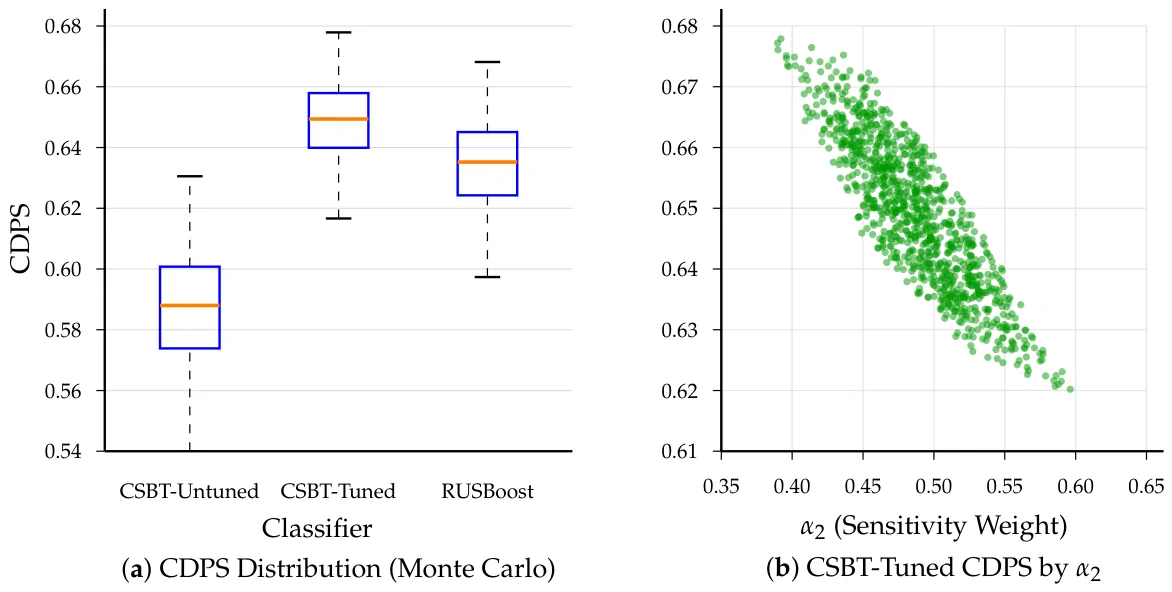
Sensitivity analysis of CDPS expert weights. (**a**) Distribution of CDPS values across 10,000 Monte Carlo samples from the theoretically justified weight space. Box plots display median, interquartile range, and range for each model. CSBT-Tuned maintains the highest CDPS across all sampled weight combinations, with no overlap between its distribution and those of the other models. (**b**) Relationship between sensitivity weight $\alpha_{2}$ and CDPS for the first-place model. All points correspond to CSBT-Tuned, confirming that model rankings are invariant to $\alpha_{2}$ within the theoretically justified range.

The analysis employed two complementary approaches: a grid-based enumeration of 17,885 weight combinations satisfying the constraint $\alpha_{1}+\alpha_{2}+\alpha_{3}=1$ within tolerance, and a Monte Carlo simulation of 10,000 randomly sampled weight vectors from the theoretically justified ranges ($\alpha_{1}\in \left[0.20,0.35\right]$, $\alpha_{2}\in \left[0.40,0.55\right]$, $\alpha_{3}\in \left[0.15,0.30\right]$). For each weight combination, CDPS was computed for all three models and rankings were determined. Figure 23 illustrates the CDPS distributions and their relationship to the sensitivity weight parameter $\alpha_{2}$.

The results demonstrate highly robust model rankings across the plausible weight space. CSBT-Tuned achieved first place in all weight combinations under both grid-based and Monte Carlo analyses, with RUSBoost consistently ranking second and CSBT-Untuned ranking third. The CDPS distributions reveal clear separation between models: CSBT-Tuned achieved a mean CDPS of 0.649±0.012 across the Monte Carlo samples, compared to 0.635±0.014 for RUSBoost and 0.587±0.018 for CSBT-Untuned. The absence of distributional overlap between CSBT-Tuned and the other models indicates that the ranking conclusion is not sensitive to the specific weight values chosen within the theoretically justified bounds.

Crossover analysis identified the weight thresholds at which model rankings would change. CSBT-Tuned is preferred over CSBT-Untuned whenever $\alpha_{2}>0.008$, a threshold far below the theoretical lower bound of 0.40. Similarly, CSBT-Tuned is preferred over RUSBoost whenever $\alpha_{2}>0.084$. Since the theoretically justified range for $\alpha_{2}$ spans [0.40,0.55], CSBT-Tuned dominates across the entire clinically defensible weight space.

When the analysis was extended to the broader expert elicitation ranges ($\alpha_{1}\in \left[0.21,0.42\right]$, $\alpha_{2}\in \left[0.29,0.61\right]$, $\alpha_{3}\in \left[0.18,0.49\right]$), which encompass the full variability observed across hypothetical expert profiles, CSBT-Tuned retained first place in all 10,000 Monte Carlo samples. This finding confirms that the model selection conclusion is robust not only to variation within theoretically justified bounds but also to the broader range of weight values that might emerge from actual expert elicitation with diverse stakeholder panels.

These results provide strong evidence that the selection of CSBT-Tuned as the preferred model for opioid misuse screening is not an artifact of the particular weight parameterization employed in the primary analysis. The model ranking conclusion is highly robust to variation in expert-elicited weights, supporting confidence in the practical applicability of this recommendation across clinical contexts that may differ in their precise prioritization of sensitivity, specificity, and overall discriminative ability.

### 3.12. Custom Penalty Matrix Analysis

Using the custom penalty matrix framework and the five clinical scenarios (S1–S5) established in Section 2.9.4, the expected costs for each classifier were calculated across the test partition. Table 21 presents the results of these calculations, demonstrating how model rankings shift as the penalty for false negatives increases. Under symmetric penalties (S1), the expected cost reduces to the total number of misclassifications, and CSBT-Untuned achieved the lowest expected cost (258), followed by RUSBoost (288) and CSBT-Tuned (317). This ordering reflects the raw accuracy ranking, as symmetric costs weight all errors equally.

As the false negative penalty increased, the ranking of models shifted. Under Scenario S2 (2:1 ratio), CSBT-Untuned retained the lowest expected cost (397), though the gap narrowed substantially. At Scenario S3 (3:1 ratio), a crossover occurred: RUSBoost achieved the lowest expected cost (524), with CSBT-Tuned (537) and CSBT-Untuned (536) achieving nearly equivalent costs. Under Scenarios S4 and S5, CSBT-Tuned consistently achieved the lowest expected cost, with values of 757 and 1307 respectively.

**Figure 24:**
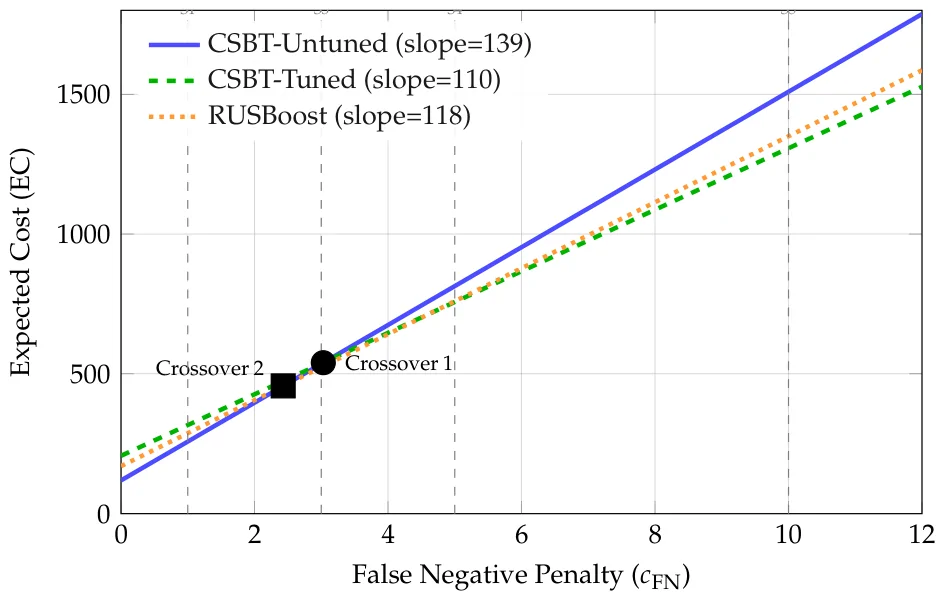
Expected cost as a function of false negative penalty $c_{\mathrm{FN}}$ with $c_{\mathrm{FP}}=1.0$. Each model’s expected cost increases linearly with $c_{\mathrm{FN}}$, with the slope determined by the number of false negatives. CSBT-Untuned achieves lowest cost at low penalty ratios but is overtaken by CSBT-Tuned at higher ratios due to its greater number of false negatives. Crossover points indicate the penalty ratios at which optimal model selection changes. Vertical dashed lines mark the penalty scenarios S1, S3, S4, and S5.

Figure 24 presents the expected cost as a continuous function of the false negative penalty $c_{\mathrm{FN}}$, with $c_{\mathrm{FP}}$ fixed at 1.0. The expected cost function for each model is linear in $c_{\mathrm{FN}}$:

$$\mathrm{EC}\left(c_{\mathrm{FN}}\right)=\mathrm{FP}+c_{\mathrm{FN}}\cdot \mathrm{FN}$$

The slopes of these lines are determined by the number of false negatives: CSBT-Untuned has the steepest slope (FN = 139), CSBT-Tuned has the shallowest slope (FN = 110), and RUSBoost is intermediate (FN = 118). The crossover points identify the cost ratios at which optimal model selection changes.

The crossover analysis identified two critical thresholds. The first crossover, between CSBT-Untuned and RUSBoost, occurred at $c_{\mathrm{FN}}=2.43$, computed as:

$$c_{\mathrm{FN}}^{*}=\frac{{\mathrm{FP}}_{\mathrm{RUS}}-{\mathrm{FP}}_{\mathrm{Unt}}}{{\mathrm{FN}}_{\mathrm{Unt}}-{\mathrm{FN}}_{\mathrm{RUS}}}=\frac{170-119}{139-118}=\frac{51}{21}=2.43$$

The second crossover, between CSBT-Untuned and CSBT-Tuned, occurred at $c_{\mathrm{FN}}=3.03$:

$$c_{\mathrm{FN}}^{*}=\frac{{\mathrm{FP}}_{\mathrm{Tun}}-{\mathrm{FP}}_{\mathrm{Unt}}}{{\mathrm{FN}}_{\mathrm{Unt}}-{\mathrm{FN}}_{\mathrm{Tun}}}=\frac{207-119}{139-110}=\frac{88}{29}=3.03$$

**Figure 25:**
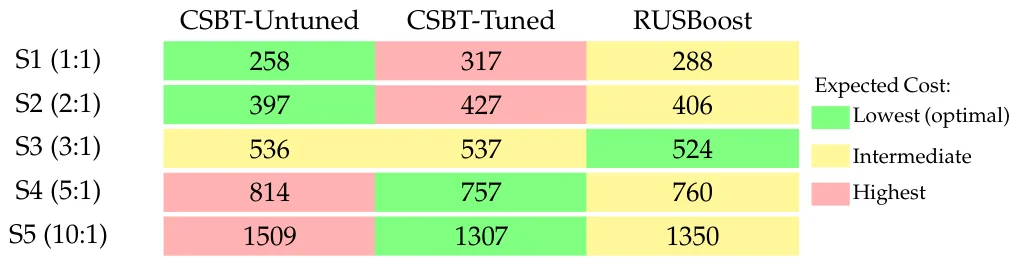
Heatmap of expected costs across penalty scenarios and models. Green cells indicate the optimal (lowest cost) model for each scenario. At low false negative penalties (S1, S2), CSBT-Untuned achieves optimal cost. At clinical screening ratios (S3), RUSBoost achieves marginal advantage. At high false negative penalties (S4, S5), CSBT-Tuned achieves optimal cost due to its lower false negative count.

These crossover values have direct clinical interpretation: when the cost of missing a case of opioid misuse exceeds approximately three times the cost of a false alarm, CSBT-Tuned becomes the cost-minimizing model choice. Figure 25 presents the expected cost values in a heatmap format, facilitating visual identification of optimal model selection across the penalty parameter space.

To quantify the cost implications of model selection, the cost savings achievable by selecting the optimal model under each penalty scenario were computed. Table 22 presents these values, expressed both in absolute terms and as percentages relative to the worst-performing model.

The cost savings analysis revealed that the penalty for suboptimal model selection was largest under extreme penalty scenarios. Under S5 (10:1 ratio), selecting CSBT-Untuned instead of CSBT-Tuned would incur 202 additional cost units, representing a 13.4% increase in expected cost. Conversely, under S1 (symmetric penalties), selecting CSBT-Tuned instead of CSBT-Untuned would incur 59 additional cost units, an 18.6% increase.

The normalized cost per prediction provides a scale-independent measure of model efficiency. Under S3 (clinical screening ratio of 3:1), the normalized expected cost ranged from 270.5 per 1000 predictions (RUSBoost) to 277.2 per 1000 predictions (CSBT-Tuned). This narrow range indicates that at clinically plausible penalty ratios near the crossover threshold, model selection has modest impact on total expected cost.

### 3.13. Utility-Weighted and Decision-Analytic Evaluation

#### 3.13.1. Expected Utility Calculations

To evaluate model performance within a decision-analytic framework, expected utility (EU) was computed for each classifier under multiple utility parameterizations. The expected utility formulation integrates the probabilities of each confusion matrix outcome with their corresponding clinical valuations:

$$\mathrm{EU}=P\left(\mathrm{TP}\right)\cdot U_{\mathrm{TP}}+P\left(\mathrm{FP}\right)\cdot U_{\mathrm{FP}}+P\left(\mathrm{TN}\right)\cdot U_{\mathrm{TN}}+P\left(\mathrm{FN}\right)\cdot U_{\mathrm{FN}}\;$$

The outcome probabilities were derived from model performance metrics and disease prevalence. At the observed test set prevalence of π=0.1167 (226 positive cases among 1937 observations), the probability calculations yielded the values presented in Table 23.

Three utility scenarios were evaluated to reflect distinct clinical value structures, presented in Table 24. Scenario U1 (moderate asymmetry) represents a clinical context where false negatives carry approximately 2.5 times the disutility of false positives. Scenario U2 (high FN aversion) assigns false negatives ten times the disutility of false positives. Scenario U3 (balanced utilities) assigns equal disutility to both error types.

**Figure 26:**
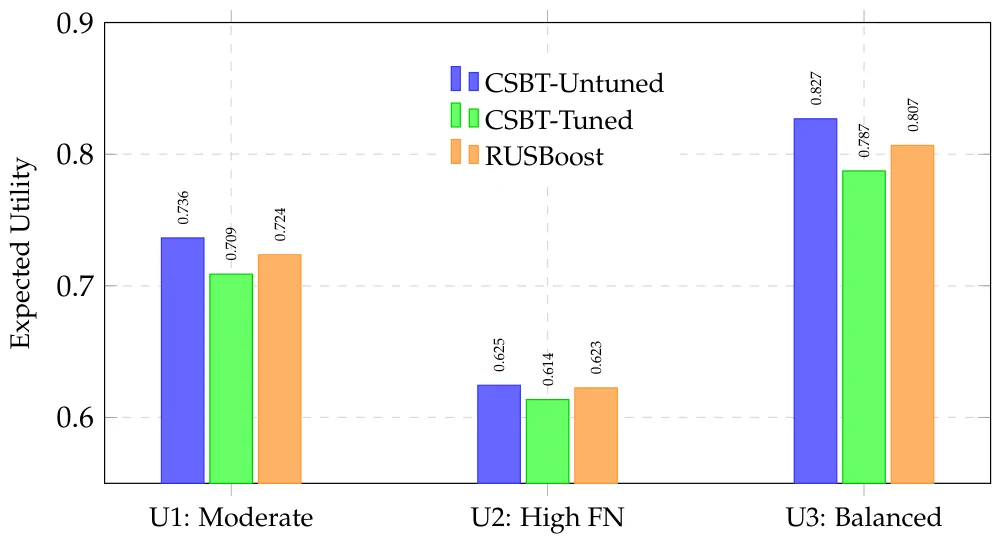
Expected utility comparison across models and utility scenarios. Scenario U1 assigns moderate false negative aversion ($U_{\mathrm{FN}}/U_{\mathrm{FP}}=2.5$); Scenario U2 assigns high false negative aversion ($U_{\mathrm{FN}}/U_{\mathrm{FP}}=10$); Scenario U3 assigns balanced error utilities. CSBT-Untuned achieves highest expected utility across all scenarios due to the dominance of true negative contributions at low prevalence, though the advantage diminishes under high false negative aversion.

Table 25 presents the expected utility calculations and the decomposition of component contributions for each model across the three clinical scenarios. Under Scenario U1 (moderate asymmetry), CSBT-Untuned achieved the highest total expected utility (0.7364), followed by RUSBoost (0.7236) and CSBT-Tuned (0.7089). This model ranking was preserved across Scenario U2 (high FN aversion) and Scenario U3 (balanced), as visualized in the comparative bar chart in Figure 26. To identify the utility parameterization at which model rankings would change, a crossover analysis was conducted; this determined that CSBT-Tuned becomes optimal only when the false negative disutility ($U_{\mathrm{FN}}$) exceeds −1.83.

The mathematical basis for this crossover analysis relies on the expected utility difference between CSBT-Untuned and CSBT-Tuned, expressed as a function of the false negative disutility $U_{\mathrm{FN}}$:

$$\Delta \mathrm{EU}={\mathrm{EU}}_{\mathrm{Unt}}-{\mathrm{EU}}_{\mathrm{Tun}}=\Delta_{\mathrm{TN}}+\Delta_{\mathrm{FP}}+\Delta_{\mathrm{FN}}$$

where the differences in component contributions depend on the utility values. Setting ΔEU=0 and solving for $U_{\mathrm{FN}}$ yields the crossover point.

#### 3.13.2. Net Benefit and Decision Curve Analysis

Net benefit was computed for each model across a range of treatment thresholds $p_{t}$ from 0.05 to 0.50. The net benefit formula relates classifier performance to the exchange rate between true positive benefit and false positive harm:

$$\mathrm{NB}\left(p_{t}\right)=\frac{\mathrm{TP}}{n}-\frac{\mathrm{FP}}{n}\cdot \frac{p_{t}}{1-p_{t}}$$

The term $p_{t}/\left(1-p_{t}\right)$ represents the odds at the treatment threshold, converting false positives to the scale of true positives based on their relative disvalue. At $p_{t}=0.10$, a decision maker values avoiding one false positive as equivalent to 0.111 true positives; at $p_{t}=0.30$, this exchange rate increases to 0.429.

Table 26 presents net benefit calculations for all three models across selected treatment thresholds, along with the default strategies of treating all patients and treating none.

At low treatment thresholds ($p_{t}\leq 0.15$), CSBT-Tuned achieved the highest net benefit among classifiers. At $p_{t}=0.10$, CSBT-Tuned’s net benefit of 0.0480 exceeded CSBT-Untuned (0.0381) by 26.0% and RUSBoost (0.0461) by 4.1%. This advantage reflects CSBT-Tuned’s superior sensitivity: when the threshold is low (indicating high tolerance for false positives), the benefit of additional true positive detections outweighs the cost of accompanying false positives.

At intermediate thresholds ($p_{t}=0.20$ to 0.30), RUSBoost achieved the highest net benefit, with values of 0.0339, 0.0266, and 0.0182 at thresholds 0.20, 0.25, and 0.30 respectively. At these thresholds, RUSBoost’s balanced sensitivity-specificity profile provided optimal performance.

**Figure 27:**
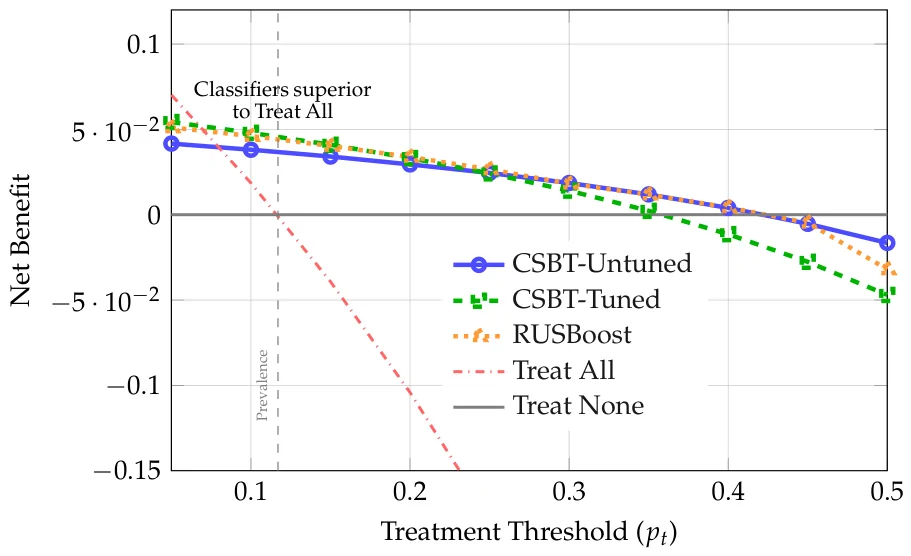
Decision curve analysis comparing net benefit across treatment thresholds. All three classifiers achieve positive net benefit and outperform the “Treat All” strategy for thresholds above approximately 0.08. CSBT-Tuned achieves highest net benefit at low thresholds ($p_{t}<0.18$); RUSBoost achieves highest net benefit at intermediate thresholds ($0.18<p_{t}<0.45$). The “Treat None” line (net benefit = 0) serves as the lower reference. The vertical dashed line indicates the observed prevalence (11.7%).

At high thresholds ($p_{t}\geq 0.40$), all classifiers approached or fell below zero net benefit, indicating that the cost of false positives exceeded the benefit of true positives. The “Treat None” strategy achieved equal or superior net benefit beyond threshold 0.40 for CSBT-Tuned and beyond threshold 0.50 for the other models. Figure 27 presents the complete decision curve analysis across the clinically relevant threshold range.

The decision curve analysis identified distinct ranges of clinical utility for each model. All three classifiers achieved positive net benefit across the threshold range 0.05 to approximately 0.45, demonstrating clinical value superior to either default strategy. The crossover between “Treat All” and the classifiers occurred at $p_{t}\approx 0.08$, below which universal intervention achieves higher net benefit than classifier-guided intervention.

The area between the classifier curve and the “Treat None” reference line quantifies the integrated net benefit across thresholds. Table 27 presents the integrated net benefit for each model over clinically relevant threshold ranges.

RUSBoost achieved the highest integrated net benefit over the full screening range (0.10 to 0.30), with an INB of 0.00650 compared to 0.00621 for CSBT-Tuned and 0.00570 for CSBT-Untuned. Over the high-sensitivity range (0.10 to 0.20), CSBT-Tuned achieved the highest INB (0.00406), consistent with its superior performance at low thresholds.

#### 3.13.3. Cost-Effectiveness Analysis

A cost-effectiveness analysis was conducted to evaluate the economic implications of classifier-guided screening strategies. The analysis adopted a healthcare system perspective, incorporating direct costs of screening, follow-up assessment, and downstream healthcare utilization, along with health outcomes measured in quality-adjusted life years (QALYs).

Table 28 presents the cost and effectiveness parameters used in the analysis. Parameter values were derived from published literature on substance use screening programs and opioid-related healthcare costs.

The total cost per patient screened was computed as:

$$C_{\mathrm{total}}=C_{\mathrm{screen}}+P\left(\mathrm{Pos}\right)\times C_{\mathrm{followup}}+P\left(\mathrm{TP}\right)\times C_{\mathrm{intervention}}+P\left(\mathrm{FN}\right)\times C_{\mathrm{untreated}}\;$$

where P(Pos)=P(TP)+P(FP) represents the probability of a positive screen.

Total QALYs per patient were computed as:

$$Q_{\mathrm{total}}=P\left(\mathrm{TP}\right)\times Q_{\mathrm{TP}}+P\left(\mathrm{FP}\right)\times Q_{\mathrm{FP}}+P\left(\mathrm{TN}\right)\times Q_{\mathrm{TN}}+P\left(\mathrm{FN}\right)\times Q_{\mathrm{FN}}$$

Table 29 presents the cost-effectiveness calculations for each classifier and comparator strategies.

All classifier-guided strategies achieved lower total costs and better health outcomes than no screening, indicating dominance of screening over non-intervention. Among classifiers, CSBT-Untuned achieved the lowest total cost ($754.88 per patient) due to its lower rate of positive screens requiring follow-up assessment. However, CSBT-Untuned also achieved the worst health outcomes (−0.00345 QALYs) due to its higher false negative rate.

The incremental cost-effectiveness ratio (ICER) for moving from CSBT-Untuned to RUSBoost was $6463 per QALY gained. The ICER for moving from RUSBoost to CSBT-Tuned was $13,840 per QALY gained. Both values fall well below standard willingness-to-pay thresholds of $50,000 to $100,000 per QALY, indicating that the additional expenditure required for higher-sensitivity screening is cost-effective.

**Figure 28:**
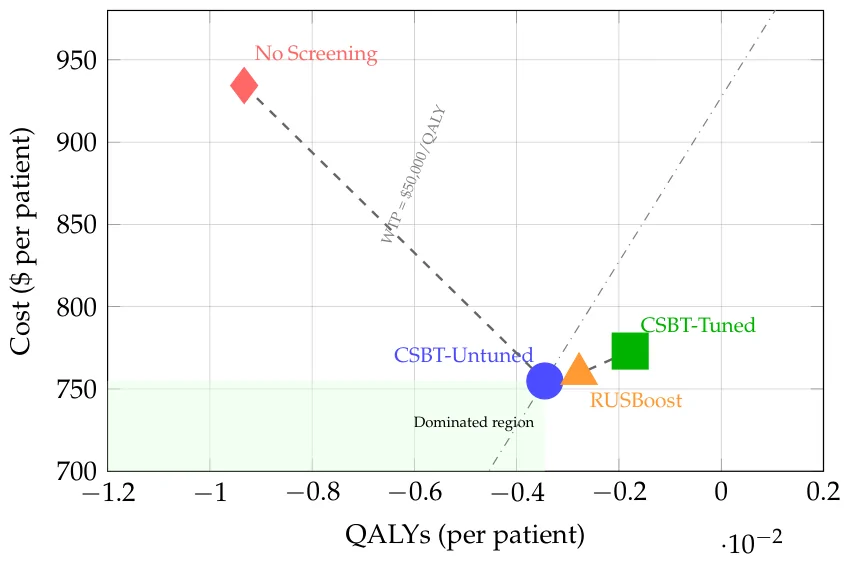
Cost-effectiveness plane showing cost and QALY outcomes for each screening strategy. The dashed line connects strategies on the cost-effectiveness frontier. Strategies below and to the right of a reference point are cost-effective relative to that reference. The “No Screening” strategy is dominated by all classifier-guided strategies (higher cost and worse outcomes). The dash–dot line represents a willingness-to-pay threshold of $50,000 per QALY; strategies below this line from any reference point are cost-effective.

Figure 28 presents the cost-effectiveness plane illustrating the positioning of each strategy.

A probabilistic sensitivity analysis was conducted using Monte Carlo simulation with 10,000 iterations to characterize uncertainty in the cost-effectiveness estimates. Parameter values were sampled from distributions reflecting plausible uncertainty: costs were sampled from gamma distributions with coefficient of variation 0.25; QALY impacts were sampled from beta distributions with coefficient of variation 0.30.

**Figure 29:**
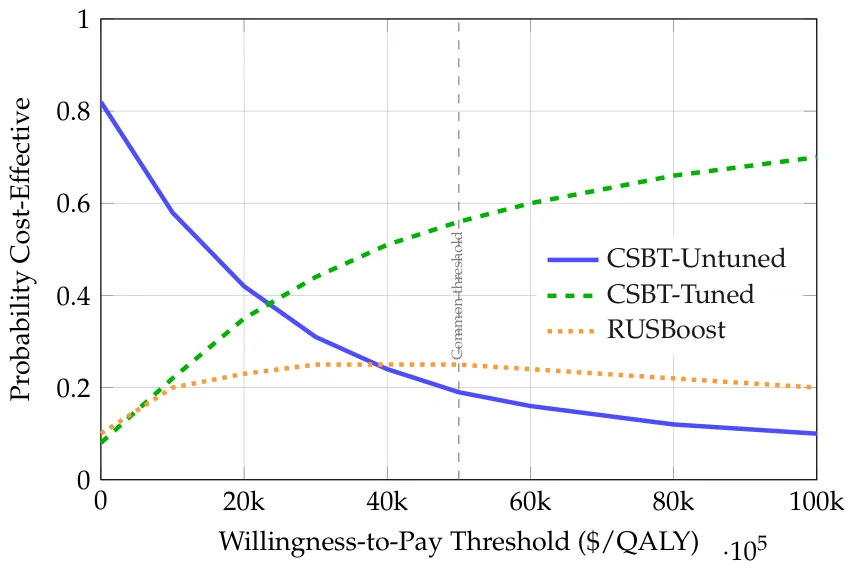
Cost-effectiveness acceptability curves from probabilistic sensitivity analysis. Each curve shows the probability that a strategy is cost-effective as a function of willingness-to-pay threshold. At low thresholds, CSBT-Untuned has highest probability of being cost-effective due to its lower cost. At thresholds above approximately $25,000/QALY, CSBT-Tuned has highest probability of being cost-effective due to its superior health outcomes. RUSBoost remains intermediate across the threshold range.

Figure 29 presents the cost-effectiveness acceptability curves showing the probability that each strategy is cost-effective across a range of willingness-to-pay thresholds. At willingness-to-pay thresholds below $15,000 per QALY, CSBT-Untuned had the highest probability of being cost-effective (58% at $10,000/QALY). At thresholds above $25,000 per QALY, CSBT-Tuned had the highest probability of being cost-effective, reaching 70% at $100,000/QALY. RUSBoost maintained an intermediate probability (20% to 25%) across most threshold values, reflecting its position between the two CSBT variants on both cost and effectiveness dimensions.

## 4. Discussion

The results of this evaluation demonstrate that aggregate performance metrics, while mathematically rigorous, are often insufficient for assessing the practical utility of predictive models in high-stakes healthcare contexts. Detailed validation through multidimensional reporting is critical for ensuring that models are clinically appropriate and ready for deployment, as consolidated reporting guidelines emphasize the need for transparent, comprehensive evidence to judge clinical readiness [33,34]. Relying on final metrics is inadequate because no individual benchmark can fully reflect the clinical consequences of classification errors; therefore, reporting a suite of complementary metrics through composite performance profiles is necessary to align model evaluation with real-world clinical utility [35,36]. Furthermore, examining internal learning behavior (specifically assessing stability and convergence through learning curve analysis) provides vital diagnostics regarding a model’s learning dynamics that aggregate statistics simply cannot reveal [22,26,37]. This study serves as a template for such reporting, providing a framework that respects both the rigor of statistical analysis and the complexity of clinical decision making.

### 4.1. The Accuracy Paradox and Clinical Model Selection

The results of this study provide a compelling illustration of the accuracy paradox in machine learning classification, i.e., a phenomenon wherein models achieving superior overall accuracy may paradoxically offer inferior practical utility for their intended application. As documented in the machine learning literature, accuracy fails to maintain reliability for imbalanced data modeling, potentially misleading model selection when minority class detection is the primary objective [38,39].

The CSBT-Untuned model’s 86.7% test accuracy initially appears superior to CSBT-Tuned’s 83.6%. However, this 3.1 percentage point accuracy advantage is achieved entirely through conservative classification that minimizes false positives at the expense of detecting true cases. With only 87 true positives against 139 false negatives, the model misses nearly two-thirds of individuals with opioid misuse history, which is a performance profile unsuitable for screening applications where identifying at-risk individuals is paramount.

In contrast, the CSBT-Tuned model’s threshold optimization explicitly prioritizes minority class detection by maximizing F1 score rather than accuracy. The resulting model is unique among the three evaluated: it is the only classifier where true positives (116) exceed false negatives (110) on the held-out test set. This distinction is not merely statistical but carries direct clinical implications. For every 226 individuals with actual opioid misuse history in the test population, CSBT-Tuned correctly identifies 116 while missing 110, whereas CSBT-Untuned identifies only 87 while missing 139. The tuned model thus detects 29 additional cases per test-set-equivalent population, which is a 33% improvement in case detection that could translate to meaningful intervention opportunities in real-world deployment.

The accuracy paradox observed here aligns with established findings that accuracy is not always the appropriate evaluation metric for classification problems involving rare events or imbalanced classes. When class distributions are skewed, as in opioid misuse prediction where positive cases constitute only 12% of observations, a model can achieve deceptively high accuracy by predominantly predicting the majority class [10,11,22]. The practical consequence is that “accurate” models may offer little value for their intended screening purpose.

These findings support a broader principle in applied machine learning: model selection criteria must align with deployment objectives. For screening applications, targeting rare but consequential outcomes (such as identifying individuals at risk for opioid misuse) metrics emphasizing minority class detection (recall, F1 score, or application-specific cost functions) provide more meaningful guidance than overall accuracy. The 3.1% accuracy reduction incurred by threshold optimization represents an acceptable trade-off for the 33% improvement in positive case detection, particularly given the severe health consequences associated with undetected opioid misuse disorders.

### 4.2. Limitations and Future Work

Several limitations warrant consideration. First, while CSBT-Tuned achieves the best minority class detection among the evaluated models, its 51.3% recall indicates that nearly half of positive cases remain undetected. This ceiling may reflect fundamental limitations in predicting complex behavioral outcomes from survey data, inherent noise in self-reported measures, or the absence of predictive features not captured in the NSDUH instrument. Second, the improvement in recall comes at the cost of reduced precision (35.9% for CSBT-Tuned versus 42.2% for CSBT-Untuned), meaning a larger proportion of positive predictions are false alarms. The acceptability of this trade-off depends on the downstream consequences of false positives in the specific deployment context.

The use of hypothetical rather than actual clinical experts represents a methodological simplification appropriate for framework demonstration; however, the validity of the evaluation approach does not depend on these specific profiles because the weight bounds are derived from decision-analytic principles and the clinical characteristics of opioid misuse, and the sensitivity analysis confirms that model selection conclusions are robust across the full range of theoretically justified weights. Future applications of this framework should employ actual multidisciplinary expert panels, and the swing weight methodology presented here provides a structured protocol for such elicitation.

Future work should also explore additional approaches to further improve minority class detection, including asymmetric cost functions with empirically calibrated cost ratios, ensemble methods combining multiple imbalance-handling strategies, and deep learning architectures capable of learning complex nonlinear relationships in high-dimensional survey data. Additionally, prospective validation studies would help establish the real-world clinical utility of these models for early identification and intervention in populations at risk for prescription opioid misuse.

### 4.3. Clinical Interpretability Through Detailed Model Diagnostics

Beyond summary statistics, examining individual confusion matrix partitions and learning curve trajectories provides direct visibility into model error structures and operating point mechanics.

While all three models achieved comparable scalar F1 scores, their underlying error distributions diverge substantially in ways that dictate clinical applicability. As established by the confusion matrix decomposition, CSBT-Tuned achieves an inverted error profile wherein positive detections exceed missed cases, whereas the untuned baseline concentrates errors as unflagged positive cases. Evaluating operating-point error distributions reveals how decision threshold calibration directly alters the clinical sensitivity-specificity trade-off, providing operational clarity that aggregate summary scores conceal.

The learning curve analysis provides complementary diagnostic information regarding model stability and data efficiency. The convergence of training and validation curves observed for the CSBT models, wherein training accuracy decreased while validation accuracy increased until the curves met at approximately 80% of training data, indicates that these approaches transitioned from an overfitting regime at small sample sizes to stable generalization at larger sample sizes. The subsequent plateau in validation accuracy beyond 40% of training data suggests that performance limitations stem from model capacity or feature informativeness rather than insufficient training observations. In contrast, the continued improvement trajectory of RUSBoost learning curves at 100% data utilization indicates potential for enhanced performance with expanded datasets, an important consideration for longitudinal studies incorporating additional survey years.

The decreasing training accuracy observed in the CSBT models warrants particular attention, as this pattern contradicts conventional expectations that training performance should increase or remain stable with additional data. This phenomenon arises from the cost-sensitive learning objective, wherein the model optimizes for weighted misclassification cost rather than raw accuracy. As training set size increases, the model encounters greater diversity in positive class presentations, requiring more conservative decision boundaries that reduce apparent training accuracy while improving generalization to the validation set. The simultaneous increase in validation accuracy confirms that this training accuracy reduction reflects improved generalization rather than degraded model quality.

The combination of confusion matrix inspection and learning curve analysis reveals information inaccessible through aggregate metrics. Specifically, the observation that CSBT-Tuned achieves a true positive count exceeding its false negative count, combined with the convergence and plateau in its learning curves, suggests a model that has achieved clinically meaningful minority class detection at a stable operating point. Neither finding would be apparent from examining accuracy, precision, recall, or F1 scores in isolation. Healthcare applications require this level of diagnostic transparency to inform deployment decisions, threshold selection, and expectations regarding model behavior across patient populations.

These findings support the broader recommendation that machine learning studies in clinical domains should routinely report complete confusion matrices and learning curves alongside aggregate performance metrics [22,28,36,40]. The convergence characteristics of learning curves indicate whether models have achieved stable performance or may benefit from additional data collection, while full confusion matrix breakdowns quantify the operational balance between diagnostic sensitivity and false alarm burden.

### 4.4. Feature Importance Interpretation and Model Selection Validity

The clinical interpretability of feature importance analyses depends critically on the validity of the underlying model from which importance values are derived. This study demonstrates that feature importance extracted from a model selected solely on aggregate performance metrics may yield misleading clinical insights, as such models may fail to capture the relationships most relevant to minority class prediction.

Had the CSBT-Untuned model been selected as the preferred classifier based on its superior overall accuracy (86.7% versus 83.6% for CSBT-Tuned), subsequent feature importance analysis would have been derived from a model that failed to detect 61.5% of individuals with documented opioid misuse history. Any clinical conclusions regarding risk factors for prescription opioid misuse drawn from such a model would be fundamentally compromised: the model’s feature importances would reflect predictors useful for classifying the majority (non-misuse) class rather than predictors that discriminate individuals at risk for the outcome of clinical interest.

The CSBT-Tuned model, by contrast, was selected based on criteria directly aligned with clinical screening objectives: it achieved the only confusion matrix configuration where true positives exceeded false negatives, and its learning curves demonstrated stable convergence without evidence of continued improvement that would suggest underfitting. Feature importance values from this model therefore reflect predictors that contribute to identifying positive cases specifically, rather than predictors that merely maximize overall classification accuracy through majority class dominance.

The prominence of lifetime alcohol use (alcever) and lifetime heroin use (herever) as top predictors aligns with clinical understanding of polysubstance use patterns and shared vulnerability factors across substance use disorders [41,42]. The importance of age category (catage) and marital status (irmarit) suggests that life stage transitions may represent periods of elevated risk warranting targeted screening. The contribution of major depressive episode (amdeyr) supports established evidence for mood disorder comorbidity in opioid misuse populations and suggests potential value in integrated mental health and substance use screening protocols [41,43,44,45,46].

These interpretations carry clinical validity precisely because they emerge from a model demonstrated to meaningfully detect the outcome of interest. Feature importance analyses from models with poor minority class performance, regardless of their aggregate accuracy, should be interpreted with substantial caution, as the identified “important” features may primarily explain majority class membership rather than the clinically relevant minority outcome [47]. This principle extends beyond the present application: any machine learning study seeking to derive clinical insights from feature importance should first establish that the source model achieves adequate detection of the outcome driving the clinical question, rather than relying on aggregate metrics that may obscure critical performance failures.

### 4.5. Clinical Evaluation Framework and Model Selection

The application of the CDPS framework to the present study illustrates a principle central to clinically oriented evaluation: the identification of an optimal model depends on the clinical context in which that model will be deployed. Whereas standard machine learning evaluation treats performance metrics as fixed quantities and generic MCDM methods apply domain-agnostic aggregation rules, the proposed framework derives its composite metrics from clinically grounded parameterizations that directly reflect the consequences of diagnostic errors in medical screening contexts. The three models evaluated in this study achieved similar aggregate performance metrics, with F1 scores ranging from 40.28% to 42.86% and accuracy ranging from 83.6% to 86.7%. Traditional evaluation approaches that rely on such aggregate metrics would provide limited guidance for model selection. The CDPS framework, by contrast, reveals meaningful distinctions that emerge only when clinical priorities are explicitly incorporated into the evaluation. The resulting model rankings across the four primary evaluation frameworks are summarized in Table 30.

The sensitivity priority weighting scheme ($\alpha_{2}=0.60$) represents the most clinically appropriate configuration for opioid misuse screening applications. In this context, the cost of failing to identify an individual with opioid misuse history substantially exceeds the cost of generating a false alarm. A missed case may result in continued substance misuse without intervention, whereas a false positive leads to additional assessment that ultimately confirms no intervention is indicated. This asymmetry, emphasized in the clinical evaluation literature, justifies weighting sensitivity more heavily than specificity for screening purposes.

Under sensitivity priority weighting, CSBT-Tuned achieved a CDPS of 0.623, representing a 13.5% relative improvement over CSBT-Untuned (0.549). This improvement quantifies the clinical value of threshold optimization: by adjusting the decision threshold from the default 0.50 to the optimized value of 0.33, the CSBT-Tuned model detects 51.3% of positive cases compared to 38.5% for CSBT-Untuned. The CDPS framework successfully formalizes this clinical gain, whereas unweighted accuracy criteria incorrectly penalize the tuned model due to majority-class dominance.

The convergence of CDPS values under specificity priority conditions (all models within 0.007) indicates that when false alarm minimization is the primary objective, the choice among these three models matters less. All three models achieve specificity exceeding 87%, and the modest differences in this metric do not translate into clinically meaningful distinctions when specificity is heavily weighted. This finding suggests that threshold optimization and class imbalance handling techniques provide their greatest value in screening contexts where sensitivity is prioritized.

The present results also demonstrate the relationship between the CDPS framework and the model selection criteria employed in this study. The CSBT-Tuned model was identified as the preferred classifier based on two criteria: superior minority class detection (the only model achieving TP > FN on the test set) and stable learning curve convergence. The CDPS analysis confirms this selection by demonstrating that CSBT-Tuned achieves superior performance under the clinical weighting scheme most appropriate for screening applications. The concordance between these two evaluation approaches—one based on confusion matrix characteristics and learning dynamics, the other based on weighted composite metrics—strengthens confidence in the model selection decision.

These findings carry implications for machine learning studies in clinical domains more broadly. Evaluation frameworks that treat all errors as interchangeable fail to capture the asymmetries inherent in medical decision making [1,22]. The CDPS and related clinically oriented metrics provide a principled approach for incorporating clinical judgment into model evaluation, ensuring that the selected model is not merely statistically optimal but clinically appropriate for its intended application.

### 4.6. Predictive Utility and the Sensitivity-PPV Trade-Off

The CPUS analysis reveals a tension that the CDPS analysis alone does not capture: the trade-off between discriminative performance and predictive utility. CSBT-Tuned was identified as the preferred model based on CDPS analysis, achieving the highest score under sensitivity priority conditions due to its superior detection of positive cases. However, the same threshold adjustment that improved sensitivity also degraded PPV, resulting in inferior CPUS performance under conditions where the reliability of positive predictions is prioritized.

This pattern has important implications for clinical deployment. A positive prediction from CSBT-Tuned is correct approximately 35.9% of the time, compared to 42.2% for CSBT-Untuned. In practical terms, approximately two-thirds of individuals flagged by CSBT-Tuned as having opioid misuse history do not, in fact, have such history. Whether this trade-off is acceptable depends on the consequences of false positive predictions in the specific deployment context.

In screening applications where positive predictions trigger low-cost, non-invasive follow-up assessments, the reduction in PPV may be acceptable in exchange for improved case detection. The additional false positives represent individuals who receive unnecessary follow-up but are ultimately reassured that no intervention is indicated. In contexts where positive predictions trigger stigmatizing labels, employment consequences, or invasive interventions, the lower PPV of CSBT-Tuned may be problematic despite its superior sensitivity.

The uniformly high NPV across all models (exceeding 0.92) reflects the influence of disease prevalence on predictive values. When prevalence is low, as in the present sample where approximately 11.6% of individuals have opioid misuse history, negative predictions are reliable regardless of model sensitivity because most individuals in the population are truly negative. This finding suggests that all three models can confidently rule out opioid misuse history when they predict its absence, with fewer than 8% of negative predictions being incorrect.

The complementary perspectives provided by CDPS and CPUS underscore the value of clinically oriented evaluation frameworks. A complete assessment of model suitability requires consideration of both discriminative ability (how well does the model separate positive from negative cases?) and predictive utility (what does a positive or negative prediction mean for the individual patient?). Models that excel on one dimension may not excel on the other, and the appropriate balance depends on the clinical context and the relative costs of different error types. For opioid misuse screening, where identifying at-risk individuals for further assessment is the primary objective and false positives lead to additional evaluation rather than immediate intervention, the CDPS results favor CSBT-Tuned. In contexts where the consequences of false positive predictions are more severe, the CPUS results suggest that CSBT-Untuned may provide superior predictive utility despite its lower sensitivity.

### 4.7. Endpoint Accuracy and Class-Balanced Evaluation

The WEAS analysis provides further evidence that aggregate performance metrics can obscure clinically meaningful distinctions between models. The complete reversal of model rankings between raw accuracy (CSBT-Untuned highest) and WEAS (CSBT-Tuned highest) demonstrates that model selection depends critically on whether the evaluation framework treats both outcome classes as equally important or allows majority class performance to dominate the assessment.

For binary classification problems such as the present opioid misuse prediction task, the WEAS reduces to balanced accuracy, which assigns equal weight to correct classification of positive and negative cases regardless of their prevalence. This property is particularly relevant in clinical screening contexts where both types of classification errors carry meaningful consequences: false negatives represent missed opportunities for early intervention, whereas false positives result in unnecessary follow-up evaluation. The WEAS framework makes explicit the implicit assumption of class equality that raw accuracy obscures through its prevalence-weighted averaging.

The gap between raw accuracy and WEAS observed in this study (ranging from 0.140 to 0.209 across models) reflects a general property of imbalanced classification: when one class substantially outnumbers the other, raw accuracy becomes insensitive to minority class performance. In the present dataset, where approximately 88% of observations belong to the negative class, a model that classified all observations as negative would achieve 88% raw accuracy while providing zero clinical utility for identifying individuals with opioid misuse history. The WEAS framework penalizes such degenerate solutions by treating per-class accuracies symmetrically.

The WEAS framework offers additional value for potential extensions of the present work to multi-class ordinal outcomes. If opioid misuse were characterized along a severity continuum (for example: no misuse, experimental use, regular misuse, severe misuse, opioid use disorder), the full WEAS formulation would enable differential weighting of endpoint accuracy versus intermediate class accuracy. Clinical decision making in such contexts often depends primarily on distinguishing the endpoints: is the individual free of problematic use, or does the individual meet criteria for severe disorder requiring intensive intervention? Errors among intermediate categories, while undesirable, may carry less immediate clinical consequence than misclassifying an individual as healthy when severe disorder is present, or vice versa.

The convergence of WEAS, CDPS under balanced weighting, and the AUC values (0.66 to 0.70 for CSBT-Tuned) reflects the mathematical relationships among these metrics when applied to binary classification. All three approaches share the property of treating both classes symmetrically, and all three identify CSBT-Tuned as the preferred model. This convergence strengthens confidence in the model selection decision by demonstrating that the conclusion is robust across multiple evaluation frameworks that share the common assumption of equal class importance.

### 4.8. Balancing Accuracy and Error Minimization

The CEPM analysis reveals a fundamental tension in classifier evaluation that neither raw accuracy nor balanced accuracy alone captures: the trade-off between minimizing total errors and achieving balanced per-class performance [2]. CSBT-Untuned achieved the lowest total error rate (13.3%) but the worst balanced accuracy (65.8%), whereas CSBT-Tuned achieved the highest balanced accuracy (69.6%) but the worst error rate (16.4%). The CEPM framework, through its balancing parameter λ, provides a principled mechanism for navigating this trade-off according to clinical priorities.

The crossover point at λ≈0.39 carries direct clinical interpretation. Below this threshold, the evaluation framework values total error minimization more heavily than balanced per-class performance. In such contexts, a model that makes fewer total errors is preferred even if those errors are concentrated among the minority class. Above this threshold, the framework values balanced per-class performance more heavily, and a model that detects minority class cases more reliably is preferred even if it makes more total errors.

For opioid misuse screening applications, the appropriate value of λ depends on the relative costs of the two component metrics. If resources for follow-up assessment are severely constrained and each positive prediction triggers substantial downstream costs, lower values of λ may be appropriate, favoring models that minimize false alarms even at the cost of reduced sensitivity. If the primary objective is early identification of individuals at risk, with follow-up assessment being relatively low-cost, higher values of λ are appropriate, favoring models that achieve balanced detection of both classes.

The adaptation of CEPM to binary classification warrants methodological comment. In the original multi-class formulation, the distance penalty differentiates between errors of varying severity: confusing healthy with severely diseased (distance 4) incurs greater penalty than confusing healthy with mildly affected (distance 1). In binary classification, this severity differentiation is absent because all errors have unit distance. The penalty therefore reduces to the overall error rate, and the CEPM becomes a weighted combination of balanced accuracy and raw accuracy. This mathematical simplification preserves the utility of the λ parameter for balancing competing objectives but loses the severity-weighting property that motivates the CEPM in multi-class contexts.

Future extensions of this work to ordinal severity outcomes, such as a graded scale from no misuse through mild, moderate, and severe misuse, would restore the full functionality of the CEPM framework. In such settings, the penalty term would differentiate between misclassifications of varying clinical severity, penalizing confusion between no misuse and severe misuse more heavily than confusion between adjacent severity grades. This property would align model evaluation with clinical decision making, where distinguishing extreme categories typically carries greater consequence than distinguishing intermediate categories.

The convergence of evaluation frameworks observed in this study, with CDPS, WEAS, and CEPM at high λ all favoring CSBT-Tuned, provides robust support for model selection when balanced per-class performance is prioritized. The divergence at low λ values, where CEPM favors CSBT-Untuned, highlights that this consensus depends on the assumption that both classes merit equal consideration. When total error minimization takes precedence, as might occur in resource-constrained settings, the model selection calculus changes accordingly.

### 4.9. Clinical Implications of Penalty Matrix and Crossover Analysis

The custom penalty matrix framework provides a principled approach to translating clinical cost considerations into quantitative model selection criteria. Our crossover analysis identified $c_{\mathrm{FN}}^{*}=3.03$ as the critical threshold at which CSBT-Tuned becomes cost-optimal relative to CSBT-Untuned. This value has direct clinical interpretation: when the clinical value of preventing one missed case of opioid misuse is worth accepting approximately three false alarms, the threshold-optimized model achieves lower total expected cost despite its higher raw error rate.

**Figure 30:**
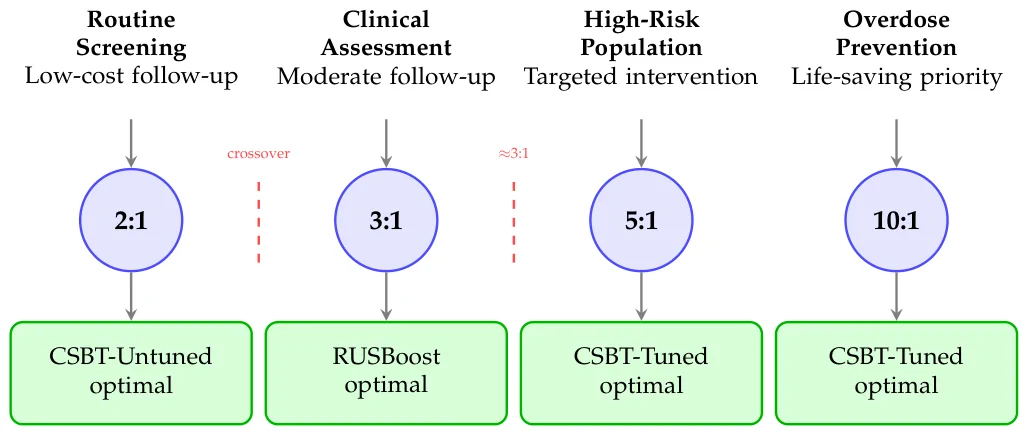
Mapping of clinical contexts to approximate penalty ratios and optimal model selection. Routine screening with low-cost follow-up procedures may warrant penalty ratios near 2:1, favoring CSBT-Untuned. Clinical assessment contexts with moderate follow-up costs correspond to ratios near 3:1, where model performance converges. High-risk populations and overdose prevention contexts justify ratios of 5:1 or higher, favoring CSBT-Tuned. The red dashed lines indicate the crossover region where optimal model selection transitions.

The clinical plausibility of this threshold depends on the specific screening context. For instance, routine screening with low-cost follow-up procedures may warrant penalty ratios near 2:1, whereas high-risk populations and overdose prevention contexts justify ratios of 5:1 or higher, making CSBT-Tuned the clearly superior choice. Figure 30 maps these clinical contexts to approximate penalty ratios based on downstream intervention characteristics.

#### Sensitivity and Practical Implementation

The narrowness of cost differences at intermediate penalty ratios, such as the 2.4% difference observed under a 3:1 ratio, suggests that in contexts where the appropriate ratio is uncertain, model selection has a limited impact on total expected cost. However, the cost differences amplify substantially at extreme penalty ratios, where selecting the wrong model could increase expected costs by over 13%. Table 31 presents the sensitivity of optimal model selection to penalty ratio uncertainty across different screening contexts.

**Figure 31:**
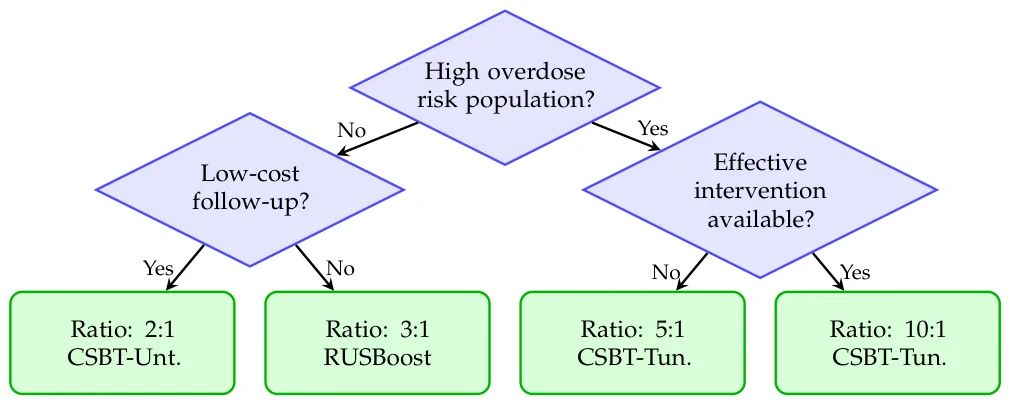
Decision flowchart for penalty ratio selection. The primary branch distinguishes high-risk from standard populations. For standard populations, follow-up cost determines whether lower (2:1) or moderate (3:1) ratios apply. For high-risk populations, intervention availability determines whether moderate-high (5:1) or high (10:1) ratios apply. The recommended model for each terminal node is based on cost-optimality analysis.

To guide practitioners, we propose the decision flowchart in Figure 31, which bases penalty selection on population risk and intervention characteristics. While the penalty matrix framework assumes linear costs and contains inherent uncertainties near crossover thresholds, it remains a transparent mechanism for ensuring that model selection trade-offs align with institutional priorities and ethical commitments.

### 4.10. Decision-Analytic Interpretation of Model Performance

#### 4.10.1. Reconciling Expected Utility and Clinical Composite Metrics

The expected utility analysis revealed an apparent paradox: under most utility parameterizations, CSBT-Untuned achieved higher expected utility than CSBT-Tuned, despite CSBT-Tuned’s superior performance on sensitivity-prioritized composite metrics such as CDPS. This divergence warrants careful interpretation, as it illuminates fundamental differences between metric-based and utility-based evaluation frameworks.

**Figure 32:**
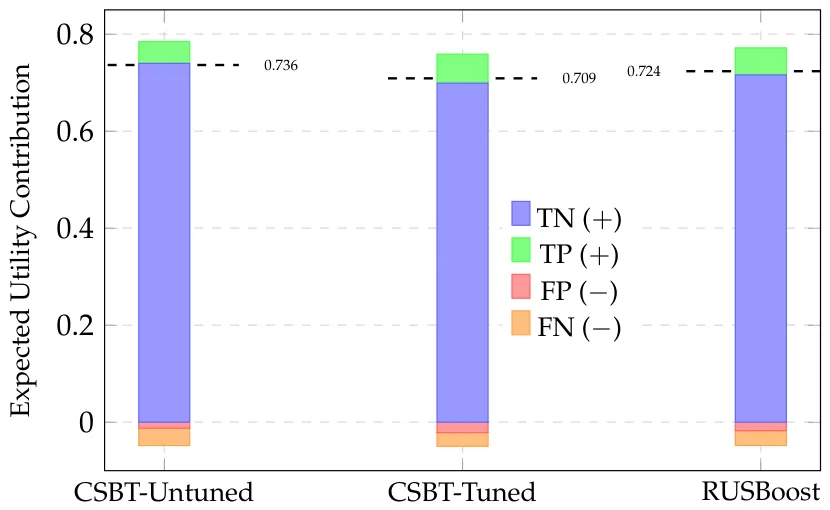
Decomposition of expected utility under Scenario U1 (moderate asymmetry). The stacked bars show the contribution of each outcome type to total expected utility. True negative contributions (blue) dominate due to high prevalence of negative cases and positive utility of correct classification. CSBT-Untuned’s advantage derives primarily from its superior TN contribution, which outweighs CSBT-Tuned’s advantages in TP contribution and reduced FN disutility. Dashed horizontal lines indicate total expected utility for each model.

The explanation lies in the interaction between prevalence and the probability weights in the expected utility calculation. At the observed prevalence of 11.7%, true negative outcomes dominate the probability distribution. For CSBT-Untuned, P(TN)=0.822 compared to P(TN)=0.777 for CSBT-Tuned. This 4.5 percentage point difference in true negative probability, when multiplied by the positive utility of correct negative classification ($U_{\mathrm{TN}}=0.80$ to 0.90), generates substantial utility advantage for CSBT-Untuned that offsets its inferior performance on the minority positive class. Figure 32 presents a decomposition of expected utility contributions that clarifies this relationship.

This finding has important implications for model selection in low-prevalence screening contexts. Composite metrics such as CDPS with sensitivity-priority weighting implicitly assume that improvements in sensitivity carry weight proportional to the specified $\alpha_{2}$ coefficient, regardless of prevalence. In contrast, expected utility calculations naturally incorporate prevalence through the probability weights, such that sensitivity improvements have diminishing marginal utility as prevalence decreases.

The practical implication is that expected utility analysis may recommend different models than composite metric analysis, even when both frameworks incorporate the same clinical priorities. This divergence is not a failure of either approach but rather reflects their different scopes: composite metrics evaluate discriminative performance independent of prevalence, while expected utility evaluates total clinical value in a specific population context.

Table 32 summarizes the model rankings under composite metrics versus expected utility.

#### 4.10.2. Clinical Interpretation of Net Benefit Results

The decision curve analysis provided actionable guidance for model selection based on the treatment threshold that characterizes a clinical context. The treatment threshold $p_{t}$ represents the probability of disease at which a clinician would be indifferent between intervening and not intervening. This threshold implicitly encodes the ratio of harms from unnecessary intervention to harms from missed disease:

$$p_{t}=\frac{{\mathrm{Harm}}_{\mathrm{FP}}}{{\mathrm{Harm}}_{\mathrm{FP}}+{\mathrm{Harm}}_{\mathrm{FN}}}\;$$

At $p_{t}=0.10$, the implied harm ratio is ${\mathrm{Harm}}_{\mathrm{FN}}/{\mathrm{Harm}}_{\mathrm{FP}}=9$. At $p_{t}=0.25$, the implied ratio is 3. At $p_{t}=0.50$, the implied ratio is 1 (equal harms).

**Figure 33:**
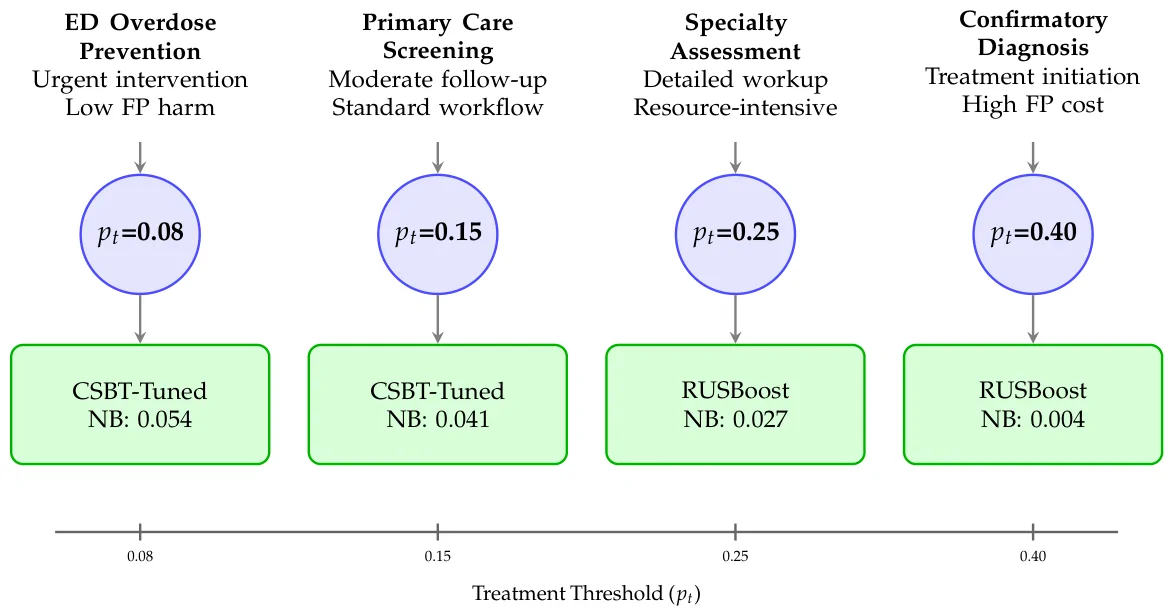
Mapping of clinical contexts to treatment thresholds and optimal models based on decision curve analysis. Emergency department and primary care settings, where missing a case carries high cost and follow-up is relatively non-invasive, correspond to low thresholds ($p_{t}<0.20$) favoring CSBT-Tuned. Specialty assessment and confirmatory diagnosis settings, where positive screens trigger resource-intensive evaluation, correspond to higher thresholds ($p_{t}>0.20$) favoring RUSBoost.

Figure 33 maps clinical contexts to approximate treatment thresholds and the corresponding optimal model.

The crossover between CSBT-Tuned and RUSBoost occurred at approximately $p_{t}=0.18$. Below this threshold, CSBT-Tuned’s superior true positive detection outweighs its higher false positive rate. Above this threshold, RUSBoost’s more balanced performance achieves higher net benefit. CSBT-Untuned achieved the lowest net benefit among classifiers across most of the threshold range, reflecting the cost of its low sensitivity even when specificity is highly valued.

The range over which each classifier achieved positive net benefit (superior to “Treat None”) provides a measure of clinical robustness. CSBT-Tuned maintained positive net benefit for $p_{t}\in \left[0.05,0.37\right]$; RUSBoost maintained positive net benefit for $p_{t}\in \left[0.05,0.47\right]$; CSBT-Untuned maintained positive net benefit for $p_{t}\in \left[0.05,0.45\right]$. RUSBoost thus demonstrated the widest range of clinical utility, though CSBT-Tuned achieved higher peak net benefit at low thresholds.

#### 4.10.3. Cost-Effectiveness Implications for Implementation

The cost-effectiveness analysis demonstrated that all classifier-guided screening strategies dominated the no-screening alternative, achieving both lower costs and better health outcomes. This finding supports the economic viability of implementing machine learning-based opioid misuse screening in healthcare settings.

The incremental analysis revealed a cost-effectiveness frontier connecting CSBT-Untuned, RUSBoost, and CSBT-Tuned in order of increasing cost and effectiveness. The ICERs along this frontier ($6463/QALY for RUSBoost relative to CSBT-Untuned; $13,840/QALY for CSBT-Tuned relative to RUSBoost) fall well below conventional willingness-to-pay thresholds, indicating that healthcare systems should be willing to incur the additional costs associated with higher-sensitivity screening.

The probabilistic sensitivity analysis revealed important uncertainty in these conclusions. At low willingness-to-pay thresholds (<$15,000/QALY), CSBT-Untuned had a 58% probability of being the most cost-effective option, reflecting its lower screening and follow-up costs. At high willingness-to-pay thresholds (>$50,000/QALY), CSBT-Tuned had a 56% probability of being most cost-effective, reflecting the value placed on avoiding false negative harms. Table 33 synthesizes the decision-analytic findings into implementation recommendations.

#### 4.10.4. Limitations of the Decision-Analytic Framework

Several limitations of the decision-analytic evaluation warrant acknowledgment. First, the utility and cost parameters were derived from literature estimates and expert judgment rather than primary data collection. The sensitivity of conclusions to parameter uncertainty was addressed through probabilistic analysis, but structural uncertainty regarding the appropriate parameter values remains.

Second, the analysis assumed a one-year time horizon and did not model long-term outcomes of early intervention versus missed detection. Opioid misuse trajectories evolve over years, and the full value of early detection may not be captured in short-term QALY estimates.

Third, the expected utility framework assumes that utilities are linear and additive across outcomes. In practice, decision makers may exhibit risk aversion or other non-linear preferences that would alter optimal model selection [48].

Fourth, the net benefit formulation assumes that the treatment threshold is known and stable across patients. In practice, treatment decisions must account for the fact that machine learning classifiers are fundamentally statistical rather than confirmatory. These tools identify conditions based on how a patient fits into broad, nationwide statistical patterns; however, there will always be specific patients who cannot be accurately described by those statistics. Relying on such a tool for a single patient is akin to prescribing treatment for condition A simply because “most people” in that statistical bucket have it, potentially ignoring alternative explanations for that specific individual’s symptoms. This inherent limitation underscores the necessity of personalized clinical judgment and the multidimensional validation described in this paper before any model is used to guide treatment for new, unseen patients.

Despite these limitations, the decision-analytic framework provides a principled approach to connecting classifier performance metrics to downstream clinical and economic outcomes. The convergence of multiple analytic approaches (expected utility, net benefit, cost-effectiveness) on the recommendation of CSBT-Tuned for high-sensitivity screening and RUSBoost for balanced applications provides robust support for these implementation recommendations.

### 4.11. Integrating Multiple Clinical Perspectives

The application of four complementary evaluation metrics to the present classification problem reveals a fundamental principle of clinically oriented model assessment: different metrics capture different aspects of clinical utility, and disagreements between metrics carry substantive meaning. A model that excels on discriminative metrics (CDPS, WEAS) may not excel on predictive utility metrics (CPUS), and vice versa. These disagreements are not failures of the metrics but rather reflections of the multi-dimensional nature of clinical performance.

Integrating CDPS, CPUS, WEAS, and CEPM demonstrates how complementary performance indices expose distinct operational trade-offs. CSBT-Tuned excels on discriminative and class-balanced dimensions (CDPS, WEAS) by rewarding minority class case detection. Conversely, CSBT-Untuned preserves higher positive predictive utility under PPV-priority constraints, and CEPM establishes an explicit crossover (λ≈0.39) where preference shifts from total error minimization to balanced class accuracy.

For opioid misuse screening applications, the integrated metric analysis supports the selection of CSBT-Tuned as the preferred model under the following conditions: (1) the primary objective is identifying individuals at risk for opioid misuse, accepting higher false positive rates in exchange for improved case detection; (2) both outcome classes are considered equally important for evaluation purposes, rather than allowing majority class performance to dominate; and (3) the clinical context can accommodate the lower positive predictive value (35.9%) associated with threshold optimization. Under these conditions, CSBT-Tuned achieves the highest point scores among viable classifiers across CDPS, WEAS, and CEPM (at λ>0.39). Although RUSBoost achieved competitive point estimates on specific composite scores, it was disqualified from final model selection due to non-convergent learning dynamics, establishing CSBT-Tuned as the top-performing viable classifier.

The exclusion of RUSBoost from model selection merits explicit discussion. Despite achieving the highest CEPM at λ=0.5 (0.271) and competitive performance across other metrics, RUSBoost exhibited learning curves that neither converged nor plateaued during training. This behavior suggests that the model’s performance on the test set may not be reproducible and that the model may be sensitive to the particular random samples encountered during training. Clinical deployment requires models with stable, predictable behavior; a model whose learning dynamics indicate ongoing instability cannot be recommended regardless of its metric performance on a single test set evaluation.

Conversely, if the clinical context prioritizes the reliability of positive predictions, such that each positive screen triggers resource-intensive follow-up, the integrated analysis supports consideration of CSBT-Untuned. This model achieves the highest CPUS under PPV-priority weighting and the highest CEPM at low λ values, reflecting its conservative classification strategy that produces fewer false alarms at the cost of reduced sensitivity.

**Figure 34:**
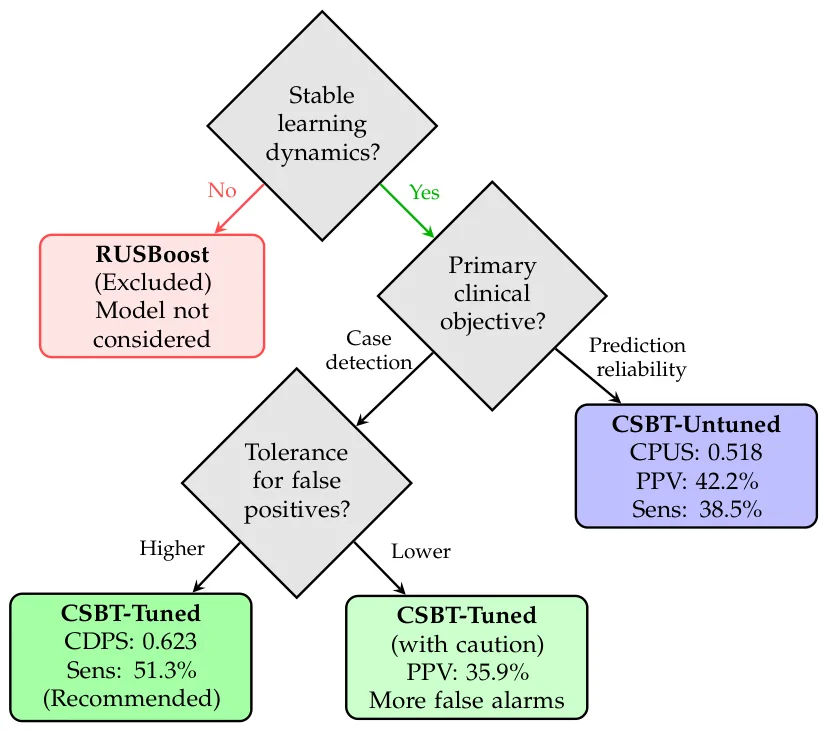
Decision framework for clinical model selection. The framework begins by assessing learning dynamics: models with non-convergent or unstable learning curves are excluded regardless of metric performance (e.g., RUSBoost achieved competitive metric values but was disqualified due to non-convergent learning curves). Among models with stable learning dynamics, the choice depends on clinical priorities. If case detection is paramount, CSBT-Tuned is preferred due to superior sensitivity (51.3%) and CDPS (0.623); tolerance for false positives determines confidence level. If prediction reliability is paramount, CSBT-Untuned is preferred due to superior PPV (42.2%), though it misses 61.5% of positive cases.

Figure 34 operationalizes these findings as a decision framework for model selection. The framework maps clinical questions to specific model recommendations between the two viable candidates: if case detection is paramount and false positives are tolerable, CSBT-Tuned is preferred; if prediction reliability is paramount and false negatives are tolerable, CSBT-Untuned is preferred.

The convergence of CDPS (under balanced weighting), WEAS, and lower-bound AUC estimates at approximately 0.66 to 0.70 for all three models deserves methodological comment. This convergence is not coincidental but reflects mathematical relationships among these metrics when applied to binary classification with AUC estimated as the average of sensitivity and specificity. All three formulations reduce to weighted combinations of sensitivity and specificity, and when weights are equal, they produce identical rankings. This convergence provides robustness to the finding that CSBT-Tuned achieves superior balanced performance: the conclusion does not depend on which specific metric is chosen, provided that metric treats both classes symmetrically.

The integrated metric framework demonstrates that comprehensive model evaluation requires explicit consideration of both clinical priorities and model stability. Reporting a single metric, whether accuracy, F1 score, or AUC, obscures the multi-dimensional nature of clinical performance and may lead to model selections that are statistically optimal but clinically suboptimal [2,49,50,51]. Furthermore, evaluating learning dynamics alongside composite metrics provides an important safeguard: non-convergent models such as RUSBoost can be identified and disqualified prior to deployment, ensuring that candidate models exhibit both statistical utility and learning stability.

### 4.12. Systematic Weight Elicitation and the Defensibility of Model Selection

The demonstration of swing weight elicitation methodology to derive CDPS weighting coefficients addresses a fundamental criticism of composite evaluation metrics: that weights are often chosen arbitrarily or, worse, adjusted post hoc to favor a preferred model. This structured elicitation demonstration, utilizing five hypothetical clinical expert profiles representing diverse stakeholder perspectives, produced documented and defensible weight selections grounded in explicit clinical reasoning rather than researcher preference.

The elicited weights revealed systematic differences in clinical priorities across stakeholder groups that would not have been apparent from predefined weighting scenarios alone. Clinicians and patient advocates consistently prioritized sensitivity, reflecting concern about missing individuals who could benefit from early intervention. The hypothetical addiction medicine profile was assigned the highest raw importance score (100) for sensitivity improvement, yielding a normalized weight of $\alpha_{2}=0.53$. The hypothetical patient advocate profile was modeled with an even stronger preference, with $\alpha_{2}=0.61$ after normalization. These priorities align with the ethical imperative in screening applications to minimize false negatives when the cost of missed cases includes preventable morbidity.

In contrast, the hypothetical hospital quality officer profile prioritized specificity ($\alpha_{3}=0.49$) to reflect simulated institutional concerns about resource allocation and the downstream costs of false positive follow-up. The epidemiologist uniquely prioritized AUC ($\alpha_{1}=0.42$) as a threshold-independent measure of overall discriminative ability, reflecting a methodological perspective that values model performance across all possible operating points. These divergent priorities are not contradictions to be resolved but rather legitimate perspectives reflecting different stakeholder roles in healthcare delivery.

The convergence of elicited weights between the predefined balanced scenario ($\alpha_{2}=0.33$) and sensitivity priority scenario ($\alpha_{2}=0.60$) carries methodological significance. The simple average of expert weights ($\alpha_{2}=0.44$) and clinically weighted average ($\alpha_{2}=0.52$) both assign greater importance to sensitivity than the balanced scenario but less than the extreme sensitivity priority scenario. This finding suggests that the predefined sensitivity priority scenario may slightly overweight sensitivity relative to expert consensus, while the balanced scenario underweights it. The elicited weights thus provide empirically grounded alternatives to predefined scenarios that may better reflect actual clinical priorities for opioid misuse screening.

The preservation of model rankings across all elicitation-based aggregation strategies strengthens confidence in the selection of CSBT-Tuned as the preferred classifier. Under simple averaging, CSBT-Tuned achieved CDPS of 0.672 compared to 0.660 for RUSBoost and 0.622 for CSBT-Untuned. Under clinical weighting, CSBT-Tuned achieved 0.659 compared to 0.639 and 0.596 respectively. The consistency of these rankings across aggregation methods indicates that the superiority of CSBT-Tuned is not an artifact of particular weight choices but reflects genuine clinical advantage for screening applications where expert priorities are systematically incorporated.

The documentation of expert rationales alongside numerical weights provides an audit trail that enhances the reproducibility and credibility of the evaluation. Future researchers can assess whether the elicitation process was appropriate for their specific clinical context, can replicate the evaluation with different expert panels, and can understand the clinical reasoning underlying particular weight selections. This transparency addresses concerns about the subjectivity of composite metrics by making the subjective elements explicit and traceable rather than hidden within arbitrary weight choices.

The disagreement among experts, rather than being a limitation, provides valuable information about the uncertainty surrounding appropriate weights. The range of sensitivity weights from 0.29 to 0.61 across the five experts spans nearly the entire plausible range, indicating genuine uncertainty about how heavily sensitivity should be weighted relative to other metrics. This disagreement motivates the sensitivity analysis reported in the results, which demonstrates that model rankings are robust across the full range of expert opinion rather than dependent on particular weight values within that range.

**Figure 35:**
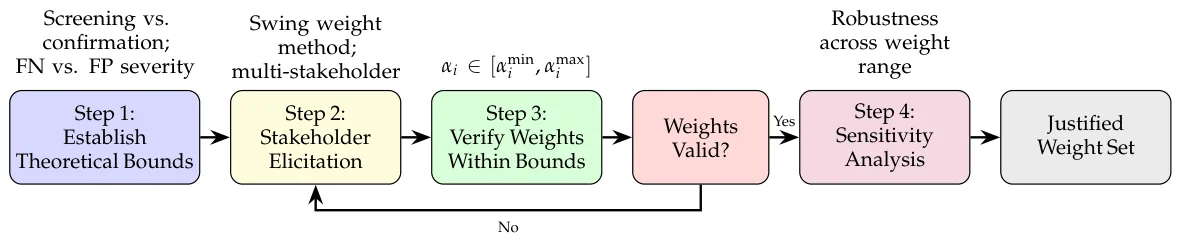
Recommended workflow for multi-source weight justification in clinical machine learning evaluation. Theoretical bounds are established based on the clinical context (Step 1), structured elicitation captures stakeholder priorities (Step 2), consistency between elicited and theoretical bounds is verified (Step 3), and sensitivity analysis assesses robustness of conclusions across plausible weights (Step 4). If elicited weights fall outside theoretical bounds, the elicitation process is revisited to reconcile stakeholder judgments with clinical constraints.

For practitioners applying this framework in other clinical contexts, we recommend the following approach to weight justification, illustrated schematically in Figure 35. First, establish theoretical bounds based on the screening-versus-confirmation distinction and the relative severity of false negative versus false positive consequences for the specific condition under study. Second, conduct structured elicitation with relevant stakeholders using swing weight or similar methodology. Third, verify that elicited weights fall within theoretical bounds; if elicited weights fall outside the justified range, the elicitation process should be revisited to reconcile stakeholder judgments with clinical constraints. Fourth, perform sensitivity analysis across the range of plausible weights to assess robustness of model selection conclusions. This multi-source justification strategy reduces dependence on any single weight derivation method while ensuring that the final weights reflect both clinical reasoning and stakeholder priorities.

### 4.13. Limitations of Weight Elicitation in the Present Study

Several limitations of the weight elicitation process warrant acknowledgment. The expert panel of five members, while representing diverse stakeholder perspectives, constitutes a small sample from which to generalize about clinical priorities. A larger panel might reveal additional perspectives or produce different aggregated weights. The selection of experts was purposive rather than random, potentially introducing selection bias if the chosen experts hold views unrepresentative of their broader professional communities.

The swing weight methodology, while structured, still requires experts to make difficult comparative judgments about the value of metric improvements. Some experts may find such comparisons counterintuitive or may interpret the elicitation questions differently, introducing measurement error into the weight estimates. The use of worst and best plausible bounds, while grounded in observed performance ranges, involves subjective judgment that affects the resulting weights.

The elicitation was conducted for a single clinical application (opioid misuse screening) and may not generalize to other screening contexts. The relative priorities of sensitivity, specificity, and AUC may differ substantially for screening applications with different prevalence rates, different consequences of false positives and false negatives, or different downstream interventions triggered by positive predictions. The weights elicited in this study should be interpreted as context-specific rather than universally applicable.

Despite these limitations, the systematic elicitation process represents a substantial improvement over arbitrary weight selection or post hoc adjustment. The documented disagreement among experts, the preservation of model rankings across aggregation methods, and the alignment of elicited weights with predefined clinical scenarios all support the validity of the weight selection process. Future work should explore larger expert panels, alternative elicitation methodologies, and cross-context validation of elicited weights to further strengthen the defensibility of composite metric evaluation.

### 4.14. Uncertainty Quantification and the Precision of Performance Claims

The bootstrap confidence intervals reported in this study transform the interpretation of model performance from point estimates conveying false precision to interval estimates that honestly represent the state of knowledge given the available data. The observation that CSBT-Tuned achieved sensitivity of 0.513 becomes more informative when accompanied by the 95% confidence interval [0.447, 0.580], indicating that the true sensitivity plausibly ranges from approximately 45% to 58% given sampling variability.

The asymmetric precision of component metrics carries important implications for model evaluation. The standard error for sensitivity (0.032 to 0.034 across models) was approximately five times larger than for specificity (0.006 to 0.009), reflecting the smaller effective sample size for the positive class (*n* = 226) compared to the negative class (*n* = 1711). This asymmetry means that sensitivity estimates carry substantially greater uncertainty than specificity estimates, a consideration that should inform the confidence placed in sensitivity-based model comparisons. Claims about sensitivity differences between models require more caution than claims about specificity differences because the former are estimated with lower precision.

The near-overlap of sensitivity confidence intervals between CSBT-Untuned [0.323, 0.451] and CSBT-Tuned [0.447, 0.580] provides statistical context for the observed point estimate difference (0.513 versus 0.385). The lower bound of the CSBT-Tuned interval (0.447) falls only 0.004 below the upper bound of the CSBT-Untuned interval (0.451), suggesting that the sensitivity improvement is statistically significant at approximately the 95% level but not with overwhelming confidence. This finding tempers the interpretation of the 33% relative improvement in sensitivity: while the point estimate suggests substantial clinical benefit from threshold optimization, the confidence intervals indicate that the true improvement could be smaller than the point estimate suggests.

The substantial overlap of sensitivity confidence intervals between CSBT-Tuned [0.447, 0.580] and RUSBoost [0.412, 0.544] indicates that the observed difference (0.513 versus 0.478) may not be statistically reliable. The true sensitivities of these two models could be equal or even reversed from the observed point estimates. This finding suggests that the preference for CSBT-Tuned over RUSBoost, while supported by point estimates, should be held with less confidence than the preference for CSBT-Tuned over CSBT-Untuned.

The propagation of uncertainty through composite scores reveals that CDPS confidence intervals inherit the uncertainty of their component metrics. Under sensitivity priority weighting, the CSBT-Tuned interval [0.579, 0.668] does not overlap with the CSBT-Untuned interval [0.509, 0.590], confirming that the CDPS improvement is statistically significant at the 95% level. This finding provides stronger evidence for the clinical value of threshold optimization than the point estimates alone, as it demonstrates that the observed CDPS difference exceeds what would be expected from sampling variability.

The convergence of CDPS confidence intervals under specificity priority weighting, where all three models achieved intervals spanning approximately 0.75 to 0.80, indicates that model selection under this scenario cannot be justified on statistical grounds. The observed differences in CDPS point estimates (0.768 to 0.775) fall well within the range of sampling variability. When specificity is heavily weighted, the choice among these three models should be based on secondary considerations such as interpretability, computational efficiency, or robustness to distributional shift rather than on CDPS values that are statistically indistinguishable.

### 4.15. Robustness of Model Rankings Across Weight Uncertainty

The sensitivity analysis across the range of expert-elicited weights demonstrates that model rankings are robust to uncertainty in weight specification. CSBT-Tuned maintained the highest CDPS throughout the entire range of sensitivity weights from 0.29 to 0.61, with its advantage over CSBT-Untuned increasing from 2.6% at low sensitivity weights to 13.1% at high sensitivity weights. This robustness provides confidence that the selection of CSBT-Tuned is not an artifact of particular weight choices but reflects genuine clinical superiority across the range of priorities expressed by clinical experts.

The increasing relative advantage of CSBT-Tuned at higher sensitivity weights aligns with clinical intuition. As screening applications place greater emphasis on case detection, the superior sensitivity of the threshold-optimized model provides greater clinical value. The 13.1% relative advantage at $\alpha_{2}=0.61$ (the maximum expert weight for sensitivity) quantifies the clinical benefit of threshold optimization for stakeholders who most heavily prioritize sensitivity, such as patient advocates and addiction medicine specialists.

The preservation of model ranking order (CSBT-Tuned, RUSBoost, CSBT-Untuned) across all weight values within the expert range indicates that disagreement among experts about appropriate weights does not translate into disagreement about which model is preferred. This concordance strengthens the model selection decision: even though experts disagree about how heavily to weight sensitivity relative to other metrics, they would all prefer CSBT-Tuned regardless of which expert’s weights were adopted.

The convergence of all models toward similar CDPS values at the lower end of the sensitivity weight range ($\alpha_{2}=0.29$) suggests that expert disagreement about model selection increases at lower sensitivity weights. When sensitivity is weighted minimally, the differences between models become smaller and may fall within the range of statistical uncertainty. This finding implies that the strength of the case for CSBT-Tuned depends on how heavily the clinical context values sensitivity: in contexts where sensitivity is paramount, CSBT-Tuned is clearly preferred; in contexts where sensitivity is less important, the model selection decision is less clear-cut.

### 4.16. Implications for Reporting and Interpretation

The uncertainty quantification methodology employed in this study supports several recommendations for reporting machine learning results in clinical applications. First, confidence intervals should accompany all point estimates for performance metrics, enabling readers to assess whether observed differences between models exceed sampling variability. Second, when comparing models, explicit statements about whether confidence intervals overlap should supplement claims about point estimate differences. Third, sensitivity analysis across plausible weight ranges should be reported for composite metrics to reveal whether conclusions are robust or sensitive to weight specification.

The pattern of results in this study illustrates how uncertainty-aware evaluation can inform model selection decisions. The statistically significant CDPS improvement of CSBT-Tuned over CSBT-Untuned under sensitivity priority weighting supports a confident selection decision. The statistically indistinguishable CDPS values under specificity priority weighting suggest that model selection in that context should be based on considerations other than CDPS. The substantial overlap of confidence intervals between CSBT-Tuned and RUSBoost across most scenarios indicates that the observed superiority of CSBT-Tuned may partially reflect sampling variability, warranting cautious interpretation of the preference ranking.

These findings also carry implications for sample size planning in future studies. The relatively wide confidence intervals for sensitivity-related metrics (reflecting the smaller positive class sample size) suggest that studies seeking to detect sensitivity differences between models require larger samples of the positive class than might be inferred from overall sample size. For rare outcomes such as opioid misuse, this consideration may motivate oversampling strategies, case-control designs, or multi-year data collection to achieve adequate precision for clinically meaningful comparisons.

### 4.17. Integration of Extended Methodological Framework

The systematic weight elicitation and uncertainty quantification extensions applied in this study address two distinct but related concerns about composite metric evaluation: the subjectivity of weight selection and the precision of performance claims. Together, these extensions strengthen the defensibility of model selection decisions by grounding weights in documented clinical judgment and by quantifying the statistical reliability of observed performance differences.

Triangulating expert swing-weight elicitation, Monte Carlo parameter sensitivity, and bootstrap resampling confirms that CSBT-Tuned remains the robust choice for screening workflows while transparently bounding claims where sampling confidence intervals overlap (e.g., relative to RUSBoost). This joint framework establishes a defensible protocol for validating clinical classifiers under realistic deployment constraints.

The methodological extensions employed in this study are not specific to the opioid misuse screening application but generalize to any classification problem where composite metrics guide model evaluation. The swing weight elicitation procedure can be applied whenever clinical experts are available to express priorities among competing performance dimensions. The bootstrap confidence interval methodology can be applied whenever a held-out validation set is available for model evaluation. The sensitivity analysis framework can be applied whenever weights carry uncertainty from expert disagreement or imprecise elicitation. Together, these tools provide a comprehensive methodology for clinically oriented model evaluation that acknowledges and addresses the subjective and statistical uncertainties inherent in performance assessment.

Future methodological development should explore the integration of these extensions with formal decision analysis frameworks that incorporate the costs and benefits of different clinical outcomes. The weights elicited from experts reflect ordinal preferences about metric importance but do not directly encode the monetary or health-related costs of different error types. A full decision-analytic framework would combine the composite metric evaluation with explicit cost modeling, enabling model selection decisions that maximize expected utility rather than weighted performance scores. Such integration would further strengthen the clinical relevance of machine learning evaluation in healthcare applications.

## 5. Conclusions

In machine learning classification, it is possible to achieve high accuracy that is nonetheless clinically useless. When applying task-specific AI tools to human lives, simple metrics are fundamentally insufficient. Impressive accuracy claims often mask algorithmic shortcuts that collapse the moment the AI encounters actual patients. As physicians who directly alter patients’ lives, clinicians cannot outsource their professional judgment to marketing materials. High aggregate metrics frequently result from improper training techniques, such as those that permit overfitting, numerous types of data leakage, or the exploitation of class imbalances. Beyond these technical risks, practitioners must remain mindful that machine learning models (similar to large language models) are fundamentally statistical tools. They are designed to work for patients who fit the training statistics, but they cannot be expected to serve as confirmatory evidence because specific individuals will always fall outside the bounds of aggregate data.

Using these tools in clinical practice is comparable to diagnosing a patient based on nationwide statistics: assuming that because a condition is most common among similar profiles, it must apply to the patient in front of the clinician. Such logic can lead to prescribing treatment for condition A even when a patient’s unique situation might be better explained by other conditions. Therefore, performance claims should not be accepted unless they are accompanied by the comprehensive technical and clinically oriented analyses described in this paper. When accepting to work with AI tools, physicians should demand a report similarly detailed to this manuscript, keeping this study as guidance for what an evidence report must look like before making the decision to use AI tools on their patients.

## Figures and Tables

**Table 1:** Hyperparameter settings for the three classification models.

Category	Parameter	CSBT-Untuned	CSBT-Tuned	RUSBoost
Ensemble	Number of Trees/Cycles	150	150	150
	Ensemble Method	Bag	Bag	RUSBoost
	Learning Rate (η)	—	—	0.1
Base Learner	Maximum Splits per Tree	100	100	50
Cost Matrix	False Positive Cost ($c_{FP}$)	1	1	—
	False Negative Cost ($c_{FN}$)	3	3	—
Decision Threshold	Threshold Value	0.50 (fixed)	Optimized	0.50 (default)
	Optimization Criterion	—	F1 Score	—
Reproducibility	Random Seed	42	42	42

**Table 2:** Preprocessing and data handling parameters applied across all modeling pipelines.

Parameter	Value
Survey Missing Codes	85, 94, 97, 98, 99
Positive Target Codes (Misuse)	1, 2, 3
Negative Target Code (No Misuse)	91
Row Missing Threshold	50%
Training Set Proportion	70%
Validation Set Proportion	15%
Test Set Proportion	15%
Numeric Imputation Method	Median
Numeric Scaling Method	Z-score Standardization
Categorical Encoding	One-hot (Reference Category Excluded)

**Table 3:** Learning curve analysis parameters for each modeling pipeline.

Parameter	CSBT-Untuned	CSBT-Tuned	RUSBoost
Training Fractions	10–100%	10–100%	10–100%
Fraction Increment	10%	10%	10%
Repetitions per Fraction	10	10	20
Stratification	Preserved	Preserved	Preserved
Metrics Recorded	Accuracy, Recall	Accuracy, Recall	Accuracy, AUC, F1, Recall

**Table 4:** Weight configurations for the Clinical Discriminative Performance Score (CDPS). Each scenario reflects different clinical priorities, with weights summing to 1.0. The balanced scenario assigns equal weight to sensitivity and specificity; the sensitivity-priority scenario emphasizes positive case detection; the specificity-priority scenario emphasizes negative case confirmation.

Scenario	$\alpha_{1}$ (AUC)	$\alpha_{2}$ (Sens)	$\alpha_{3}$ (Spec)	Clinical Context
Balanced	0.34	0.33	0.33	General evaluation
Sensitivity-priority	0.20	0.60	0.20	Screening applications
Specificity-priority	0.20	0.20	0.60	Confirmatory testing

**Table 5:** Weight configurations for the Clinical Predictive Utility Score (CPUS). Each scenario reflects different clinical priorities regarding the interpretation of positive and negative predictions. Weights $\omega_{1}$, $\omega_{2}$, and $\omega_{3}$ sum to 1.0 across all scenarios.

Scenario	$\omega_{1}$ (PPV)	$\omega_{2}$ (NPV)	$\omega_{3}$ (F1)	Clinical Context
Balanced	0.34	0.33	0.33	General evaluation
PPV-priority	0.60	0.20	0.20	Confirmatory workup
NPV-priority	0.20	0.60	0.20	Rule-out screening

**Table 6:** Weight configurations for the Clinical Endpoint Performance Metric (CEPM). The λ parameter controls the relative emphasis on balanced per-class accuracy (WEAS) versus overall error rate minimization (Penalty).

Scenario	λ	Evaluation Priority	Clinical Context
Penalty Priority	0.30	Error minimization	Resource-constrained settings
Balanced	0.50	Equal weighting	General evaluation
WEAS Priority	0.70	Balanced accuracy emphasis	Standard screening
Strong WEAS Priority	0.90	High minority class emphasis	High-risk population screening

**Table 7:** Custom penalty matrix configurations for expected cost analysis. The cost ratio indicates the relative penalty for false negatives versus false positives.

Scenario	$c_{\mathrm{FN}}$	$c_{\mathrm{FP}}$	Ratio	Clinical Context
S1: Symmetric	1.0	1.0	1:1	Equal error costs
S2: Moderate	2.0	1.0	2:1	Moderate FN concern
S3: Clinical	3.0	1.0	3:1	Standard screening
S4: High-risk	5.0	1.0	5:1	High-risk population
S5: Crisis	10.0	1.0	10:1	Overdose prevention

**Table 8:** Utility parameterizations for expected utility analysis. Positive utilities indicate benefit from correct classification; negative utilities indicate disutility from misclassification.

Scenario	$U_{\mathrm{TP}}$	$U_{\mathrm{TN}}$	$U_{\mathrm{FP}}$	$U_{\mathrm{FN}}$
U1: Moderate asymmetry	1.00	0.90	−0.20	−0.50
U2: High FN aversion	1.00	0.80	−0.10	−1.00
U3: Balanced	1.00	1.00	−0.30	−0.30

**Table 9:** Test set classification performance metrics for all three models.

Metric	CSBT-Untuned	CSBT-Tuned	RUSBoost
Accuracy (%)	86.68	83.64	85.13
Precision (%)	42.23	35.91	38.85
Recall/Sensitivity (%)	38.50	51.33	47.79
Specificity (%)	93.05	87.90	90.06
F1 Score (%)	40.28	42.26	42.86
True Positives (TP)	87	116	108
True Negatives (TN)	1592	1504	1541
False Positives (FP)	119	207	170
False Negatives (FN)	139	110	118
Decision Threshold	0.50	0.33	0.50

**Table 10:** Clinical Discriminative Performance Score (CDPS) under three clinical priority scenarios. Balanced accuracy (Sensitivity+Specificity)/2 serves as the threshold-specific AUC component to maintain consistency with operating-point performance. The CDPS is computed as $\mathrm{CDPS}=\alpha_{1}\cdot \mathrm{AUC}+\alpha_{2}\cdot \mathrm{Sensitivity}+\alpha_{3}\cdot \mathrm{Specificity}$, where $\alpha_{1}+\alpha_{2}+\alpha_{3}=1$.

Scenario/Weights	CSBT-Untun.	CSBT-Tuned	RUSBoost
Component Metrics (Test Set)			
Balanced Accuracy (AUC proxy)	0.658	0.696	0.690
Sensitivity	0.385	0.513	0.478
Specificity	0.931	0.879	0.901
Scenario A: Balanced ($\alpha_{1}=0.34$, $\alpha_{2}=0.33$, $\alpha_{3}=0.33$)			
CDPS	0.658	0.696	0.690
Scenario B: Sensitivity Priority ($\alpha_{1}=0.20$, $\alpha_{2}=0.60$, $\alpha_{3}=0.20$)			
CDPS	0.549	0.623	0.605
Scenario C: Specificity Priority ($\alpha_{1}=0.20$, $\alpha_{2}=0.20$, $\alpha_{3}=0.60$)			
CDPS	0.768	0.769	0.775

**Table 11:** Clinical Predictive Utility Score (CPUS) under three clinical priority scenarios. The CPUS is computed as $\mathrm{CPUS}=\omega_{1}\cdot \mathrm{PPV}+\omega_{2}\cdot \mathrm{NPV}+\omega_{3}\cdot F1$, where $\omega_{1}+\omega_{2}+\omega_{3}=1$. PPV priority reflects contexts where positive predictions trigger costly follow-up; NPV priority reflects contexts where ruling out disease confidently is essential.

Scenario/Weights	CSBT-Untun.	CSBT-Tuned	RUSBoost
Component Metrics (Test Set)			
Positive Predictive Value	0.422	0.359	0.388
Negative Predictive Value	0.920	0.932	0.929
F1 Score	0.403	0.423	0.429
Scenario A: Balanced ($\omega_{1}=0.34$, $\omega_{2}=0.33$, $\omega_{3}=0.33$)			
CPUS	0.580	0.569	0.580
Scenario B: PPV Priority ($\omega_{1}=0.60$, $\omega_{2}=0.20$, $\omega_{3}=0.20$)			
CPUS	0.518	0.486	0.504
Scenario C: NPV Priority ($\omega_{1}=0.20$, $\omega_{2}=0.60$, $\omega_{3}=0.20$)			
CPUS	0.717	0.715	0.721

**Table 12:** Weighted Endpoint Accuracy Score (WEAS) comparison across models. For binary classification, WEAS reduces to balanced accuracy, computed as the arithmetic mean of per-class accuracies: $\mathrm{WEAS}=({\mathrm{Acc}}_{0}+{\mathrm{Acc}}_{1})/2$. This metric weights both classes equally regardless of prevalence, revealing performance differences obscured by raw accuracy in imbalanced datasets.

Metric	CSBT-Untun.	CSBT-Tuned	RUSBoost
Per-Class Accuracies			
Acc_0_ (Specificity)	0.931	0.879	0.901
Acc_1_ (Sensitivity)	0.385	0.513	0.478
Aggregate Metrics			
Raw Accuracy	0.867	0.836	0.851
WEAS (Balanced Accuracy)	0.658	0.696	0.690
Metric Discrepancy			
Accuracy − WEAS	0.209	0.140	0.161

**Table 13:** Clinical Endpoint Performance Metric (CEPM) under varying balancing parameter λ. The CEPM is computed as CEPM=λ·WEAS−(1−λ)·Penalty. For binary classification, the penalty equals the misclassification rate (FP+FN)/N. Higher λ values emphasize balanced accuracy (WEAS); lower λ values emphasize error minimization. Bold values indicate the highest CEPM within each column.

Metric/Parameter	CSBT-Untuned	CSBT-Tuned	RUSBoost
Component Metrics			
WEAS (Balanced Accuracy)	0.658	0.696	0.690
Penalty (Error Rate)	0.133	0.164	0.149
CEPM at λ=0.3 (Penalty Priority)			
CEPM	**0.104**	0.094	0.103
CEPM at λ=0.5 (Balanced)			
CEPM	0.262	0.266	**0.271**
CEPM at λ=0.7 (WEAS Priority)			
CEPM	0.421	**0.438**	**0.438**
CEPM at λ=0.9 (Strong WEAS Priority)			
CEPM	0.579	**0.610**	0.606

**Table 14:** Detailed summary of clinically oriented evaluation metrics across all three models. CDPS emphasizes discriminative ability; CPUS emphasizes predictive utility; WEAS provides class-balanced accuracy; CEPM balances accuracy against error minimization. Bold values indicate the highest score within each row. The rightmost column indicates which clinical priority each scenario reflects.

Metric/Scenario	CSBT-Unt.	CSBT-Tun.	RUSBoost	Clinical Priority
Clinical Discriminative Performance Score (CDPS)				
Balanced	0.658	**0.696**	0.690	Equal weighting
Sensitivity Priority	0.549	**0.623**	0.605	Case detection
Specificity Priority	0.768	0.769	**0.775**	False alarm reduction
Clinical Predictive Utility Score (CPUS)				
Balanced	**0.580**	0.569	**0.580**	Equal weighting
PPV Priority	**0.518**	0.486	0.504	Positive prediction reliability
NPV Priority	0.717	0.715	**0.721**	Negative prediction reliability
Weighted Endpoint Accuracy Score (WEAS)				
Balanced Accuracy	0.658	**0.696**	0.690	Per-class equality
Clinical Endpoint Performance Metric (CEPM)				
λ=0.3	**0.104**	0.094	0.103	Error minimization
λ=0.5	0.262	0.266	**0.271**	Balanced
λ=0.7	0.421	**0.438**	**0.438**	Accuracy emphasis

**Table 15:** Reference bounds for swing weight elicitation established by clinical expert consensus.

Metric	Worst Plausible	Best Plausible	Range
AUC (lower bound)	0.55	0.85	0.30
Sensitivity	0.30	0.80	0.50
Specificity	0.70	0.98	0.28

**Table 16:** Hypothetical swing weight elicitation results for five expert profiles. Raw scores reflect the simulated value of improving each metric from worst to best plausible value, with the most important metric assigned 100. Normalized weights sum to 1.00 within each profile.

	Raw Importance Scores			Normalized Weights		
Expert (Role)	AUC	Sens	Spec	$\alpha_{1}$	$\alpha_{2}$	$\alpha_{3}$
Expert 1 (Addiction Medicine)	50	100	40	0.26	0.53	0.21
Expert 2 (Primary Care)	60	100	55	0.28	0.47	0.26
Expert 3 (Epidemiologist)	100	70	70	0.42	0.29	0.29
Expert 4 (Quality Officer)	45	60	100	0.22	0.29	0.49
Expert 5 (Patient Advocate)	35	100	30	0.21	0.61	0.18
Simple Average	—	—	—	0.28	0.44	0.29
Standard Deviation	—	—	—	0.08	0.14	0.12

**Table 17:** Comparison of predefined evaluation scenarios with empirically elicited weight sets. Elicited weights derive from systematic swing weight methodology applied to a five-member clinical expert panel.

Weight Source	$\alpha_{1}$ (AUC)	$\alpha_{2}$ (Sens)	$\alpha_{3}$ (Spec)
Predefined Scenarios			
Balanced	0.34	0.33	0.33
Sensitivity Priority	0.20	0.60	0.20
Specificity Priority	0.20	0.20	0.60
Elicited Weights			
Simple Average (All Experts)	0.28	0.44	0.29
Clinical Weighting	0.25	0.52	0.23
Expert Range (Min)	0.21	0.29	0.18
Expert Range (Max)	0.42	0.61	0.49

**Table 18:** Clinical Discriminative Performance Score (CDPS) computed using empirically elicited weight sets from the expert panel.

Weight Set	CSBT-Untuned	CSBT-Tuned	RUSBoost
Simple Average	0.622	0.672	0.660
Clinical Weighting	0.596	0.659	0.639

**Table 19:** Bootstrap confidence intervals (95%) for component performance metrics. Point estimates represent observed test set values; confidence intervals were constructed from B=2000 bootstrap samples using the percentile method.

Metric	CSBT-Untuned	CSBT-Tuned	RUSBoost
Sensitivity	0.385	0.513	0.478
95% CI	[0.323, 0.451]	[0.447, 0.580]	[0.412, 0.544]
SE	0.032	0.034	0.033
Specificity	0.931	0.879	0.901
95% CI	[0.917, 0.943]	[0.861, 0.896]	[0.885, 0.915]
SE	0.006	0.009	0.008
AUC (lower bound)	0.658	0.696	0.690
95% CI	[0.625, 0.691]	[0.661, 0.732]	[0.655, 0.724]
SE	0.017	0.018	0.018

**Table 20:** Bootstrap confidence intervals (95%) for CDPS under multiple weighting scenarios. Intervals were constructed from B=2000 bootstrap samples, with component metrics recomputed for each sample before applying the CDPS formula.

Scenario	CSBT-Untuned	CSBT-Tuned	RUSBoost
Balanced ($\alpha_{1}=0.34$, $\alpha_{2}=0.33$, $\alpha_{3}=0.33$)			
Point Estimate	0.658	0.696	0.690
95% CI	[0.631, 0.685]	[0.666, 0.726]	[0.660, 0.719]
Sensitivity Priority ($\alpha_{1}=0.20$, $\alpha_{2}=0.60$, $\alpha_{3}=0.20$)			
Point Estimate	0.549	0.623	0.605
95% CI	[0.509, 0.590]	[0.579, 0.668]	[0.561, 0.650]
Specificity Priority ($\alpha_{1}=0.20$, $\alpha_{2}=0.20$, $\alpha_{3}=0.60$)			
Point Estimate	0.768	0.769	0.775
95% CI	[0.749, 0.786]	[0.747, 0.790]	[0.755, 0.795]
Simple Average (Elicited) ($\alpha_{1}=0.28$, $\alpha_{2}=0.44$, $\alpha_{3}=0.29$)			
Point Estimate	0.622	0.672	0.660
95% CI	[0.591, 0.653]	[0.638, 0.706]	[0.627, 0.694]
Clinical Weighting (Elicited) ($\alpha_{1}=0.25$, $\alpha_{2}=0.52$, $\alpha_{3}=0.23$)			
Point Estimate	0.596	0.659	0.639
95% CI	[0.560, 0.632]	[0.621, 0.698]	[0.600, 0.678]

**Table 21:** Expected cost (EC) under custom penalty matrices for all three models. EC is computed as $\mathrm{EC}=c_{\mathrm{FP}}\cdot \mathrm{FP}+c_{\mathrm{FN}}\cdot \mathrm{FN}$. Bold values indicate the lowest (optimal) expected cost within each scenario. The crossover scenario identifies the cost ratio at which model rankings change.

Scenario	CSBT-Untuned	CSBT-Tuned	RUSBoost
Confusion Matrix Components (Test Set)			
False Positives (FP)	119	207	170
False Negatives (FN)	139	110	118
Expected Cost by Penalty Scenario			
S1: Symmetric (1:1)	**258**	317	288
S2: Moderate (2:1)	**397**	427	406
S3: Clinical (3:1)	536	**537**	**524**
S4: High-risk (5:1)	814	**757**	760
S5: Crisis (10:1)	1509	**1307**	1350
Normalized Expected Cost (per 1000 predictions)			
S1: Symmetric	**133.2**	163.7	148.7
S3: Clinical (3:1)	276.7	**277.2**	**270.5**
S5: Crisis (10:1)	779.0	**674.8**	697.0

**Table 22:** Cost savings from optimal model selection under each penalty scenario. Absolute savings represent the difference in expected cost between the worst and best model. Percentage savings are computed relative to the worst-performing model. The penalty for suboptimal selection column indicates the additional cost incurred by choosing the wrong model.

Scenario	Best Model	Worst Model	Savings	% Savings
S1: Symmetric (1:1)	CSBT-Unt. (258)	CSBT-Tun. (317)	59	18.6%
S2: Moderate (2:1)	CSBT-Unt. (397)	CSBT-Tun. (427)	30	7.0%
S3: Clinical (3:1)	RUSBoost (524)	CSBT-Tun. (537)	13	2.4%
S4: High-risk (5:1)	CSBT-Tun. (757)	CSBT-Unt. (814)	57	7.0%
S5: Crisis (10:1)	CSBT-Tun. (1307)	CSBT-Unt. (1509)	202	13.4%

**Table 23:** Outcome probabilities for each classifier derived from test set performance and prevalence. Probabilities are computed as: P(TP)=π×Sens, P(FN)=π×(1−Sens), P(TN)=(1−π)×Spec, P(FP)=(1−π)×(1−Spec). All probabilities sum to 1.000 within rounding error.

Outcome Probability	CSBT-Untuned	CSBT-Tuned	RUSBoost
P(TP)	0.0449	0.0599	0.0558
P(FN)	0.0718	0.0568	0.0609
P(TN)	0.8219	0.7765	0.7955
P(FP)	0.0614	0.1069	0.0878
Sum	1.0000	1.0001	1.0000

**Table 24:** Utility parameterizations for decision-analytic evaluation. Each scenario assigns utility values to the four confusion matrix outcomes reflecting different clinical priorities. $U_{\mathrm{TP}}$ and $U_{\mathrm{TN}}$ represent benefits of correct classification; $U_{\mathrm{FP}}$ and $U_{\mathrm{FN}}$ represent disutilities of misclassification. All values are on an arbitrary utility scale where higher values indicate more favorable outcomes.

Scenario	$U_{\mathrm{TP}}$	$U_{\mathrm{TN}}$	$U_{\mathrm{FP}}$	$U_{\mathrm{FN}}$
U1: Moderate asymmetry	1.00	0.90	−0.20	−0.50
U2: High FN aversion	1.00	0.80	−0.10	−1.00
U3: Balanced utilities	1.00	1.00	−0.30	−0.30

**Table 25:** Expected utility (EU) calculations under three utility scenarios. Component contributions show the utility contribution from each outcome type. Total EU represents the sum across all outcomes. Bold values indicate the highest (optimal) expected utility within each scenario.

Component/Scenario	CSBT-Untuned	CSBT-Tuned	RUSBoost
Scenario U1: Moderate Asymmetry			
TP contribution	0.0449	0.0599	0.0558
TN contribution	0.7397	0.6988	0.7159
FP contribution	−0.0123	−0.0214	−0.0176
FN contribution	−0.0359	−0.0284	−0.0305
**Total EU**	**0.7364**	0.7089	0.7236
Scenario U2: High FN Aversion			
TP contribution	0.0449	0.0599	0.0558
TN contribution	0.6575	0.6212	0.6364
FP contribution	−0.0061	−0.0107	−0.0088
FN contribution	−0.0718	−0.0568	−0.0609
**Total EU**	**0.6245**	0.6136	0.6225
Scenario U3: Balanced Utilities			
TP contribution	0.0449	0.0599	0.0558
TN contribution	0.8219	0.7765	0.7955
FP contribution	−0.0184	−0.0321	−0.0263
FN contribution	−0.0215	−0.0170	−0.0183
**Total EU**	**0.8269**	0.7873	0.8067

**Table 26:** Net benefit at selected treatment thresholds for all models and default strategies. Net benefit is computed as $\mathrm{NB}=\mathrm{TP}/n-\mathrm{FP}/n\times p_{t}/\left(1-p_{t}\right)$, where n=1937. The “Treat All” strategy assumes all patients receive intervention; the “Treat None” strategy assumes no patients receive intervention. Bold values indicate the highest net benefit among classifiers at each threshold.

Threshold ($p_{t}$)	CSBT-Unt.	CSBT-Tun.	RUSBoost	Treat All	Treat None
0.05	0.0417	**0.0543**	0.0512	0.0702	0.000
0.10	0.0381	**0.0480**	0.0461	0.0186	0.000
0.15	0.0341	**0.0410**	0.0403	−0.0392	0.000
0.20	0.0295	0.0332	**0.0339**	−0.1041	0.000
0.25	0.0245	0.0242	**0.0266**	−0.1778	0.000
0.30	0.0186	0.0141	**0.0182**	−0.2619	0.000
0.40	0.0040	−0.0114	**0.0043**	−0.4722	0.000
0.50	−0.0165	−0.0470	**−0.0320**	−0.7667	0.000

**Table 27:** Integrated net benefit (INB) computed as the area under the net benefit curve over specified threshold ranges. Higher values indicate greater clinical utility aggregated across the threshold range. The threshold range 0.10 to 0.30 represents typical screening contexts; 0.10 to 0.20 represents high-sensitivity screening; 0.20 to 0.30 represents moderate-specificity screening.

Threshold Range	CSBT-Untuned	CSBT-Tuned	RUSBoost
0.10 to 0.30 (full screening)	0.00570	0.00621	**0.00650**
0.10 to 0.20 (high-sensitivity)	0.00338	**0.00406**	0.00400
0.20 to 0.30 (moderate-specificity)	0.00232	0.00215	**0.00250**

**Table 28:** Cost and effectiveness parameters for cost-effectiveness analysis. Cost values are in 2024 US dollars. QALY impacts represent the expected change in quality-adjusted life years associated with each outcome type. Parameters reflect a healthcare system perspective with a one-year time horizon.

Parameter	Value	Source/Basis
Direct Costs		
Screening administration	$50	Per-patient cost
Follow-up assessment (if positive)	$200	Confirmatory evaluation
Early intervention program	$1500	Per-enrolled patient
Untreated opioid misuse (annual)	$8000	Healthcare utilization
QALY Impacts		
True positive (early intervention)	+0.05	Treatment benefit
False positive (unnecessary workup)	−0.005	Anxiety, inconvenience
True negative	0.00	No change (baseline)
False negative (missed case)	−0.08	Continued morbidity

**Table 29:** Cost-effectiveness analysis results. Total cost includes screening, follow-up, intervention, and downstream healthcare costs. Total QALYs represent net health impact per patient screened. The incremental cost-effectiveness ratio (ICER) compares each strategy to the next less effective alternative on the cost-effectiveness frontier.

Strategy	Cost ($/Patient)	QALYs	ΔCost	ICER ($/QALY)
No Screening	934.40	−0.00933	—	—
CSBT-Untuned	754.88	−0.00345	−179.52	Dominant
RUSBoost	759.21	−0.00278	+4.33	6463
CSBT-Tuned	773.05	−0.00178	+13.84	13,840

**Table 30:** Model rankings (1st, 2nd, 3rd) across clinically oriented evaluation metrics under screening-appropriate parameterizations. Rankings are based on CDPS with sensitivity priority, CPUS with balanced weighting, WEAS (balanced accuracy), and CEPM at λ=0.5. Note: RUSBoost was excluded from final model selection due to non-convergent learning curves, despite achieving competitive metric values. Among viable models, CSBT-Tuned achieves superior performance on discriminative metrics.

Metric	1st Place	2nd Place	3rd Place
CDPS (Sensitivity Priority)	CSBT-Tuned	RUSBoost	CSBT-Untuned
CPUS (Balanced)	CSBT-Untuned/RUSBoost	—	CSBT-Tuned
WEAS	CSBT-Tuned	RUSBoost	CSBT-Untuned
CEPM (λ=0.5)	RUSBoost	CSBT-Tuned	CSBT-Untuned
Win Count	2.0 (CSBT-Tuned)	1.5 (RUSBoost)	0.5 (CSBT-Untuned)

**Table 31:** Sensitivity analysis of expected cost to penalty ratio uncertainty.

Context	Baseline	Range	Cost Range	Stability
Routine screening	2:1	1.5–2.5	328–397	Stable (CSBT-Unt.)
Clinical assessment	3:1	2.5–4.0	457–551	Unstable
High-risk population	5:1	4.0–6.0	551–757	Stable (CSBT-Tun.)
Crisis intervention	10:1	7.0–15.0	977–1527	Stable (CSBT-Tun.)

**Table 32:** Comparison of model rankings under composite metric versus expected utility frameworks. Rankings are shown as 1st, 2nd, 3rd. The composite metrics use sensitivity-priority weighting; expected utility uses Scenario U1 (moderate asymmetry). The frameworks agree on last-place ranking (CSBT-Untuned by CDPS, CSBT-Tuned by EU) but differ on first place.

Evaluation Framework	CSBT-Unt.	CSBT-Tun.	RUSBoost
Composite Metrics (Sensitivity Priority)			
CDPS	3rd	1st	2nd
CPUS	1st	3rd	2nd
Expected Utility			
U1: Moderate asymmetry	1st	3rd	2nd
U2: High FN aversion	1st	3rd	2nd
U3: Balanced	1st	3rd	2nd

**Table 33:** Implementation recommendations based on decision-analytic evaluation. Each row corresponds to a clinical context characterized by its treatment threshold, willingness-to-pay, and primary objective. The recommended model and supporting evidence are provided for each context.

Clinical Context	$p_{t}$	WTP	Model	Evidence
Universal screening	0.10	High	CSBT-Tuned	Highest NB at $p_{t}$ = 0.10
Resource-limited	0.15	Low	RUSBoost	Balanced cost/effect
Targeted high-risk	0.08	High	CSBT-Tuned	Highest sensitivity
Confirmatory workup	0.30	Medium	RUSBoost	Positive NB at high $p_{t}$
Budget-constrained	Any	Very low	CSBT-Untuned	Lowest total cost

## Data Availability

The data presented in this study are available in the Substance Abuse and Mental Health Data Archive (SAMHDA) at https://www.datafiles.samhsa.gov/ (accessed on 10 September 2026). These data were derived from the following resources available in the public domain: National Survey on Drug Use and Health (NSDUH) for the years 2015–2019.

## Abbreviations

   The following abbreviations are used in this manuscript:

AI	Artificial Intelligence
AUC	Area Under the Receiver Operating Characteristic Curve
CDPS	Clinical Discriminative Performance Score
CEPM	Clinical Endpoint Performance Metric
CPUS	Clinical Predictive Utility Score
CSBT	Cost-Sensitive Bagged Trees
CV	Coefficient of Variation
DCA	Decision Curve Analysis
EU	Expected Utility
FN	False Negative
FP	False Positive
ICER	Incremental Cost-Effectiveness Ratio
INB	Integrated Net Benefit
MCDM	Multi-Criteria Decision Making
ML	Machine Learning
NPV	Negative Predictive Value
NSDUH	National Survey on Drug Use and Health
PPV	Positive Predictive Value
PR	Precision–Recall
QALY	Quality-Adjusted Life Year
ROC	Receiver Operating Characteristic
RUSBoost	Random Under-Sampling Boosting
TN	True Negative
TP	True Positive
WEAS	Weighted Endpoint Accuracy Score

## Appendix A. Pseudocode for Classification Pipelines

This appendix presents the pseudocode implementations for the three classification pipelines described in the main text: Cost-Sensitive Bagged Trees without threshold tuning (CSBT-Untuned), Cost-Sensitive Bagged Trees with threshold tuning (CSBT-Tuned), and RUSBoost. Additionally, shared preprocessing and evaluation functions common to all pipelines are provided. The following helper functions are shared across all three pipelines.

**Algorithm A1** Preprocessing Functions
1: **function** FitPreprocessor($D_{\mathrm{train}}$) 2: **for** each feature $x_{j}$ in $D_{\mathrm{train}}$ **do** 3: **if** $x_{j}$ is numeric **then** 4: median_*j*_ $\leftarrow \mathrm{median}\left(x_{j}\right)$ 5: Impute missing values with median_*j*_ 6: $\mu_{j}\leftarrow \mathrm{mean}\left(x_{j}\right)$; $\sigma_{j}\leftarrow \mathrm{std}\left(x_{j}\right)$ 7: **else** 8: Store unique category levels including “Missing” 9: **end if** 10: **end for** 11: **return** preprocessor parameters 12: **end function** ** ** 13: **function** TransformData(D, preprocessor) 14: **for** each numeric feature $x_{j}$ **do** 15: Impute missing with stored median_*j*_ 16: $z_{j}\leftarrow \left(x_{j}-\mu_{j}\right)/\sigma_{j}$ ▹ Z-score standardization 17: **end for** 18: **for** each categorical feature $x_{j}$ **do** 19: A pply one-hot encoding using stored levels 20: Drop first category as reference 21: **end for** 22: **return** transformed feature matrix *X* 23: **end function**

**Algorithm A2** CSBT-Untuned Pipeline
**Require:** Dataset D, target variable pnrnmrec, cost matrix C **Ensure:** Trained model, performance metrics, predictions ** // Phase 1: Data Preparation** 1: Load dataset D from CSV file 2: Replace survey missing codes {85,94,97,98,99} with NaN 3: Remove duplicate observations 4: Create binary target *Y*: positive ←{1,2,3}, negative ←{91} 5: Remove leakage variables {pnrnmyr,pnrnmevr,pnrnm30d,pnrnm30fq} **// Phase 2: Stratified Partitioning** 6: $\left(D_{\mathrm{train}},D_{\mathrm{temp}}\right)\leftarrow \mathrm{StratifiedSplit}\left(D,0.70\right)$ 7: $\left(D_{\mathrm{val}},D_{\mathrm{test}}\right)\leftarrow \mathrm{StratifiedSplit}\left(D_{\mathrm{temp}},0.50\right)$ **// Phase 3: Preprocessing (Training Set Only)** 8: Remove rows with >50% missing values from $D_{\mathrm{train}}$ 9: Fit preprocessor: compute $\mu_{j}$, $\sigma_{j}$, median_*j*_ for numeric features 10: Store category levels for categorical features 11: Transform all partitions using fitted parameters 12: Remove zero-variance features; fill residual NaN with 0 **// Phase 4: Model Training** 13: Define cost matrix: $C=\left(\begin{array}{cc} 0 & 1\\ 3 & 0 \end{array}\right)$ 14: Initialize tree template with MaxNumSplits =100 15: **for** b=1 to B=150 **do** 16: Draw bootstrap sample $S_{b}$ from $D_{\mathrm{train}}$ 17: Train cost-sensitive decision tree $h_{b}$ on $S_{b}$ using C 18: Add $h_{b}$ to ensemble 19: **end for** **// Phase 5: Evaluation** 20: ${\hat{Y}}_{\mathrm{train}}\leftarrow \mathrm{Predict}\left(\mathrm{Ensemble},X_{\mathrm{train}}\right)$ with threshold τ=0.50 21: ${\hat{Y}}_{\mathrm{val}}\leftarrow \mathrm{Predict}\left(\mathrm{Ensemble},X_{\mathrm{val}}\right)$ with threshold τ=0.50 22: ${\hat{Y}}_{\mathrm{test}}\leftarrow \mathrm{Predict}\left(\mathrm{Ensemble},X_{\mathrm{test}}\right)$ with threshold τ=0.50 23: Compute metrics: Accuracy, Precision, Recall, Specificity, F1, AUC **// Phase 6: Generate Outputs** 24: Generate ROC curves, PR curve, confusion matrices 25: Compute feature importance via predictor importance 26: Generate learning curves over training fractions {0.10,0.20,…,1.00} 27: Save model, metrics, and predictions

**Algorithm A3** CSBT-Tuned Pipeline
**Require:** Dataset D, target variable pnrnmrec, cost matrix C **Ensure:** Trained model, optimized threshold $\tau^{*}$, performance metrics ** // Phases 1–4: Identical to Algorithm A2** 1: Execute data preparation, partitioning, preprocessing, and model training **// Phase 5: Threshold Optimization on Validation Set** 2: Obtain predicted probabilities ${\hat{p}}_{\mathrm{val}}\leftarrow \mathrm{PredictProba}\left(\mathrm{Ensemble},X_{\mathrm{val}}\right)$ 3: Initialize $\tau^{*}\leftarrow 0.50$, $F_{1}^{*}\leftarrow 0$ 4: **for** τ∈{0.05,0.06,…,0.95} **do** 5: ${\hat{Y}}_{\mathrm{val}}\left(\tau \right)\leftarrow I\left({\hat{p}}_{\mathrm{val}}\geq \tau \right)$ 6: Compute $F_{1}\left(\tau \right)$ on validation set 7: **if** $F_{1}\left(\tau \right)>F_{1}^{*}$ **then** 8: $\tau^{*}\leftarrow \tau$ 9: $F_{1}^{*}\leftarrow F_{1}\left(\tau \right)$ 10: **end if** 11: **end for** 12: **return** optimal threshold $\tau^{*}$ **// Phase 6: Dual Evaluation** 13: **for** partition ∈{train,val,test} **do** 14: Evaluate at default threshold τ=0.50 15: Evaluate at optimized threshold $\tau =\tau^{*}$ 16: Record both sets of metrics 17: **end for** **// Phase 7: Generate Outputs** 18: Generate threshold tuning curve (F1, Recall, Precision vs. τ) 19: Generate confusion matrices for both thresholds 20: Generate ROC curves, PR curve, learning curves 21: Save model, $\tau^{*}$, and all metrics

**Algorithm A4** RUSBoost Pipeline
**Require:** Dataset D, target variable pnrnmrec, learning rate η **Ensure:** Trained ensemble, performance metrics ** // Phases 1–3: Identical to Algorithm A2** 1: Execute data preparation, partitioning, and preprocessing **// Phase 4: RUSBoost Training** 2: Initialize instance weights: $D_{1}\left(i\right)=1/n$ for all *i* 3: Set hyperparameters: T=150, MaxNumSplits=50, η=0.1 4: **for** t=1 to *T* **do** 5: $S_{\mathrm{minority}}\leftarrow$ all instances where $y_{i}=1$ 6: $S_{\mathrm{majority}}\leftarrow$ random undersample of instances where $y_{i}=0$ 7: $S_{t}\leftarrow S_{\mathrm{minority}}\cup S_{\mathrm{majority}}$ ▹ Balanced subset 8: Train weak learner $h_{t}$ on $S_{t}$ with weights $D_{t}$ 9: Compute weighted error: $\epsilon_{t}=\sum_{i:h_{t}\left(x_{i}\right)\neq y_{i}}D_{t}\left(i\right)$ 10: Compute learner weight: $\alpha_{t}=\frac{\eta }{2}\ln\left(\frac{1-\epsilon_{t}}{\epsilon_{t}}\right)$ 11: Update weights: $D_{t+1}\left(i\right)\propto D_{t}\left(i\right)\cdot \exp\left(-\alpha_{t}y_{i}h_{t}\left(x_{i}\right)\right)$ 12: Normalize $D_{t+1}$ 13: **end for** 14: Final classifier: $H\left(x\right)=\mathrm{sign}\left(\sum_{t=1}^{T}\alpha_{t}h_{t}\left(x\right)\right)$ **// Phases 5–6: Evaluation and Outputs** 15: Evaluate on train, validation, and test sets 16: Generate ROC curves, PR curve, confusion matrices 17: Generate learning curves with extended metrics (Accuracy, AUC, F1, Recall) 18: Compute and visualize feature importance 19: Save model and all results

**Algorithm A5** Binary Classification Metrics
1: **function** ComputeBinaryMetrics($Y_{\mathrm{true}}$, $Y_{\mathrm{pred}}$, scores) 2: $\mathrm{TP}\leftarrow \sum I(Y_{\mathrm{true}}=1\wedge Y_{\mathrm{pred}}=1)$ 3: $\mathrm{TN}\leftarrow \sum I(Y_{\mathrm{true}}=0\wedge Y_{\mathrm{pred}}=0)$ 4: $\mathrm{FP}\leftarrow \sum I(Y_{\mathrm{true}}=0\wedge Y_{\mathrm{pred}}=1)$ 5: $\mathrm{FN}\leftarrow \sum I(Y_{\mathrm{true}}=1\wedge Y_{\mathrm{pred}}=0)$ 6: Accuracy ←(TP+TN)/(TP+TN+FP+FN) 7: Precision ←TP/(TP+FP) 8: Recall ←TP/(TP+FN) 9: Specificity ←TN/(TN+FP) 10: $F_{1}\leftarrow 2\cdot \mathrm{Precision}\cdot \mathrm{Recall}/\left(\mathrm{Precision}+\mathrm{Recall}\right)$ 11: AUC $\leftarrow \mathrm{AreaUnderROC}(Y_{\mathrm{true}},\mathrm{scores})$ 12: **return** metrics structure 13: **end function**

**Algorithm A6** Stratified Learning Curve Generation
1: **function** GenerateLearningCurve($X_{\mathrm{train}}$, $Y_{\mathrm{train}}$, $X_{\mathrm{val}}$, $Y_{\mathrm{val}}$, model_type) 2: fractions ←{0.10,0.20,…,1.00} 3: numRepeats ←10 (or 20 for RUSBoost) 4: **for** each fraction *f* in fractions **do** 5: **for** r=1 to numRepeats **do** 6: $I_{0}\leftarrow$ shuffled indices where Y=0 7: $I_{1}\leftarrow$ shuffled indices where Y=1 8: $n_{0}\leftarrow \left\lfloor f\cdot \right|I_{0}\left|\right\rfloor$; $n_{1}\leftarrow \left\lfloor f\cdot \right|I_{1}\left|\right\rfloor$ 9: subset $\leftarrow I_{0}\left[1:n_{0}\right]\cup I_{1}\left[1:n_{1}\right]$ 10: Train temporary model on subset 11: Record train and validation metrics 12: **end for** 13: Store mean and std of metrics for fraction *f* 14: **end for** 15: **return** learning curve results table 16: **end function**

## References

[1] Toma M., Yusupov D. (2026). Perspectives on the Limits and Clinical Alignment of Medical AI from Population Statistics to Individual Care. Bioengineering.

[2] Ghanem M., Ghaith A.K., El-Hajj V.G., Bhandarkar A., de Giorgio A., Elmi-Terander A., Bydon M. (2023). Limitations in Evaluating Machine Learning Models for Imbalanced Binary Outcome Classification in Spine Surgery: A Systematic Review. Brain Sci..

[3] Tarek Z., Shams M.Y., Towfek S.K., Alkahtani H.K., Ibrahim A., Abdelhamid A.A., Eid M.M., Khodadadi N., Abualigah L., Khafaga D.S. (2023). An Optimized Model Based on Deep Learning and Gated Recurrent Unit for COVID-19 Death Prediction. Biomimetics.

[4] Alkhammash E.H., Hadjouni M., Elshewey A.M. (2022). A Hybrid Ensemble Stacking Model for Gender Voice Recognition Approach. Electronics.

[5] Elshewey A.M., Shams M.Y., Tarek Z., Megahed M., El-kenawy E.S.M., El-dosuky M.A. (2023). Weight Prediction Using the Hybrid Stacked-LSTM Food Selection Model. Comput. Syst. Sci. Eng..

[6] Peterson E.D., DeLong E.R., Masoudi F.A., O’Brien S.M., Peterson P.N., Rumsfeld J.S., Shahian D.M., Shaw R.E. (2010). ACCF/AHA 2010 Position Statement on Composite Measures for Healthcare Performance Assessment. J. Am. Coll. Cardiol..

[7] Elshewey A.M. (2025). Enhancing crop yield prediction based on dove optimization algorithm and gradient boosting model. Signal Image Video Process..

[8] Elshewey A.M., Jamjoom M.M., Alkhammash E.H. (2025). An enhanced CNN with ResNet50 and LSTM deep learning forecasting model for climate change decision making. Sci. Rep..

[9] Elshewey A.M. (2026). DNN-XGBoost framework for CO_2_ emissions prediction based on binary whale optimization and grey wolf optimization algorithms. Sustain. Comput. Inform. Syst..

[10] Cartus A.R., Samuels E.A., Cerdá M., Marshall B.D.L. (2023). Outcome class imbalance and rare events: An underappreciated complication for overdose risk prediction modeling. Addiction.

[11] Han D.H., Lee S., Seo D.C. (2020). Using machine learning to predict opioid misuse among U.S. adolescents. Prev. Med..

[12] Lo-Ciganic W.H., Huang J.L., Zhang H.H., Weiss J.C., Wu Y., Kwoh C.K., Donohue J.M., Cochran G., Gordon A.J., Malone D.C. (2019). Evaluation of Machine-Learning Algorithms for Predicting Opioid Overdose Risk Among Medicare Beneficiaries with Opioid Prescriptions. JAMA Netw. Open.

[13] Bolshakova M., Bluthenthal R., Sussman S. (2019). Opioid use and misuse: Health impact, prevalence, correlates and interventions. Psychol. Health.

[14] Florence C., Luo F., Rice K. (2021). The economic burden of opioid use disorder and fatal opioid overdose in the United States, 2017. Drug Alcohol Depend..

[15] Klimas J., Gorfinkel L., Fairbairn N., Amato L., Ahamad K., Nolan S., Simel D.L., Wood E. (2019). Strategies to Identify Patient Risks of Prescription Opioid Addiction When Initiating Opioids for Pain: A Systematic Review. JAMA Netw. Open.

[16] Valdes E.G., Reist C., Aamar R., Hallisey B., Stanton E.S., Williams L., Andel R., Gorman J. (2023). Use of Predictive Analytics to Identify Unhealthy Opioid Use and Guide Intervention. Psychiatr. Serv..

[17] Dowell D., Ragan K.R., Jones C.M., Baldwin G.T., Chou R. (2022). CDC Clinical Practice Guideline for Prescribing Opioids for Pain—United States, 2022. MMWR Recomm. Rep..

[18] Picco L., Jung M., Cangadis-Douglass H., Lam T., Nielsen S. (2023). Identifying Prescription-Opioid-Related Risks Using Prescription Drug Monitoring Programs’ Algorithms and Clinical Screening Tools. Pharmacy.

[19] Chhablani C., Shahid U., Parde N., Muslmani S., Hu H., Thorpe D., Afshar M., Karnik N., Chhabra N. (2026). Machine learning models to detect opioid misuse in emergency department patients at triage. Am. J. Emerg. Med..

[20] Sanchez-Pinto L.N., Bennett T.D. (2022). Evaluation of Machine Learning Models for Clinical Prediction Problems. Pediatr. Crit. Care Med..

[21] Park C., Son M., Kim J., Kim B., Ahn Y., Kwon N. (2025). TOPSIS and AHP-Based Multi-Criteria Decision-Making Approach for Evaluating Redevelopment in Old Residential Projects. Sustainability.

[22] Shin D., Chung J., Toma M. (2026). Learning dynamics vs. aggregate metrics: Clinical applicability of machine learning survival prediction in eye cancer. Acad. Biol..

[23] Seiffert C., Khoshgoftaar T.M., Van Hulse J., Napolitano A. (2010). RUSBoost: A Hybrid Approach to Alleviating Class Imbalance. IEEE Trans. Syst. Man Cybern. Part Syst. Hum..

[24] Toma M. (2025). AI-Assisted Medical Diagnostics: A Clinical Guide to Next-Generation Diagnostics.

[25] Toma M. (2026). Diagnosing AI: Evaluation of AI in Clinical Practice.

[26] Viering T., Loog M. (2023). The Shape of Learning Curves: A Review. IEEE Trans. Pattern Anal. Mach. Intell..

[27] Montesinos López O.A., Montesinos López A., Crossa J. (2022). Overfitting, Model Tuning, and Evaluation of Prediction Performance. Multivariate Statistical Machine Learning Methods for Genomic Prediction.

[28] Varoquaux G., Colliot O. (2023). Evaluating Machine Learning Models and Their Diagnostic Value. Machine Learning for Brain Disorders.

[29] Kaneko H. (2023). Interpretation of Machine Learning Models for Data Sets with Many Features Using Feature Importance. ACS Omega.

[30] Carvalho D.V., Pereira E.M., Cardoso J.S. (2019). Machine Learning Interpretability: A Survey on Methods and Metrics. Electronics.

[31] Daly J., Sher M., Remyes D., Garcia J., Toma M. (2026). A cost-effectiveness analysis of AI-assisted OCT classification for age-related macular degeneration. J. Hosp. Manag. Health Policy.

[32] Clemen R.T., Reilly T. (2013). Making Hard Decisions with DecisionTools.

[33] Klement W., El Emam K. (2023). Consolidated Reporting Guidelines for Prognostic and Diagnostic Machine Learning Modeling Studies: Development and Validation. J. Med. Internet Res..

[34] Yusuf M., Atal I., Li J., Smith P., Ravaud P., Fergie M., Callaghan M., Selfe J. (2020). Reporting quality of studies using machine learning models for medical diagnosis: A systematic review. BMJ Open.

[35] Stevens L.M., Mortazavi B.J., Deo R.C., Curtis L., Kao D.P. (2020). Recommendations for Reporting Machine Learning Analyses in Clinical Research. Circ. Cardiovasc. Qual. Outcomes.

[36] Hicks S.A., Strümke I., Thambawita V., Hammou M., Riegler M.A., Halvorsen P., Parasa S. (2022). On evaluation metrics for medical applications of artificial intelligence. Sci. Rep..

[37] Riley R.D., Collins G.S. (2023). Stability of clinical prediction models developed using statistical or machine learning methods. Biom. J..

[38] He H., Garcia E. (2009). Learning from Imbalanced Data. IEEE Trans. Knowl. Data Eng..

[39] Valverde-Albacete F.J., Peláez-Moreno C. (2014). 100% Classification Accuracy Considered Harmful: The Normalized Information Transfer Factor Explains the Accuracy Paradox. PLoS ONE.

[40] Collins G.S., Moons K.G.M., Dhiman P., Riley R.D., Beam A.L., Van Calster B., Ghassemi M., Liu X., Reitsma J.B., van Smeden M. (2024). TRIPOD + AI statement: Updated guidance for reporting clinical prediction models that use regression or machine learning methods. BMJ.

[41] Pan Y., Feaster D.J., Odom G., Brandt L., Hu M.C., Weiss R.D., Rotrosen J., Saxon A.J., Luo S.X., Balise R.R. (2022). Specific polysubstance use patterns predict relapse among patients entering opioid use disorder treatment. Drug Alcohol Depend. Rep..

[42] Klymchuk V., Gorbunova V., Ivanchuk I. (2024). Prevalence of alcohol use and depressive/anxiety symptoms among patients of opioid agonist treatment programs in Ukraine. Acad. Ment. Health Well-Being.

[43] Han B., Compton W.M., Blanco C., Crane E., Lee J., Jones C.M. (2017). Prescription Opioid Use, Misuse, and Use Disorders in U.S. Adults: 2015 National Survey on Drug Use and Health. Ann. Intern. Med..

[44] Tormohlen K.N., Mojtabai R., Seiwell A., McGinty E.E., Stuart E.A., Tobin K.E., Troiani V. (2021). Co-Occurring Opioid Use and Depressive Disorders: Patient Characteristics and Co-Occurring Health Conditions. J. Dual Diagn..

[45] Jang B.J., Schuler M.S., Evans-Polce R.J., Patrick M.E. (2018). Marital Status as a Partial Mediator of the Associations Between Young Adult Substance Use and Subsequent Substance Use Disorder: Application of Causal Inference Methods. J. Stud. Alcohol Drugs.

[46] Murray-Krezan C., Dopp A., Tarhuni L., Carmody M.D., Becker K., Anderson J., Komaromy M., Meredith L.S., Watkins K.E., Wagner K. (2023). Screening for opioid use disorder and co-occurring depression and post-traumatic stress disorder in primary care in New Mexico. Addict. Sci. Clin. Pract..

[47] Lee Y., Baruzzo G., Kim J., Seo J., Camillo B.D. (2025). Validity of Feature Importance in Low-Performing Machine Learning for Tabular Biomedical Data. IEEE Access.

[48] Alqudah A.M., Moussavi Z. (2026). CUES: A Multiplicative Composite Metric for Evaluating Clinical Prediction Models Theory, Inference, and Properties. Mathematics.

[49] Van Calster B., Collins G.S., Vickers A.J., Wynants L., Kerr K.F., Barreñada L., Varoquaux G., Singh K., Moons K.G., Hernandez-Boussard T. (2025). Evaluation of performance measures in predictive artificial intelligence models to support medical decisions: Overview and guidance. Lancet Digit. Health.

[50] Huang C., Li S.X., Caraballo C., Masoudi F.A., Rumsfeld J.S., Spertus J.A., Normand S.L.T., Mortazavi B.J., Krumholz H.M. (2021). Performance Metrics for the Comparative Analysis of Clinical Risk Prediction Models Employing Machine Learning. Circ. Cardiovasc. Qual. Outcomes.

[51] Halligan S., Altman D.G., Mallett S. (2015). Disadvantages of using the area under the receiver operating characteristic curve to assess imaging tests: A discussion and proposal for an alternative approach. Eur. Radiol..

